# Dedifferentiation‐derived neural stem cells exhibit perturbed temporal progression

**DOI:** 10.15252/embr.202255837

**Published:** 2023-04-11

**Authors:** Kellie Veen, Phuong‐Khanh Nguyen, Francesca Froldi, Qian Dong, Edel Alvarez‐Ochoa, Kieran F Harvey, John PD McMullen, Owen Marshall, Patricia R Jusuf, Louise Y Cheng

**Affiliations:** ^1^ Peter MacCallum Cancer Centre Melbourne VIC Australia; ^2^ Sir Peter MacCallum Department of Oncology The University of Melbourne Melbourne VIC Australia; ^3^ School of Biosciences The University of Melbourne Melbourne VIC Australia; ^4^ Department of Anatomy and Developmental Biology Monash University Clayton VIC Australia; ^5^ Menzies Institute for Medical Research Medical Science Precinct Hobart TAS Australia; ^6^ Department of Anatomy and Physiology The University of Melbourne Melbourne VIC Australia

**Keywords:** dedifferentiation, *Drosophila*, neuroblast, temporal transcription factors, terminal differentiation, Development, Neuroscience, Stem Cells & Regenerative Medicine

## Abstract

Dedifferentiation is the reversion of mature cells to a stem cell‐like fate, whereby gene expression programs are altered and genes associated with multipotency are (re)expressed. Misexpression of multipotency factors and pathways causes the formation of ectopic neural stem cells (NSCs). Whether dedifferentiated NSCs faithfully produce the correct number and types of progeny, or undergo timely terminal differentiation, has not been assessed. Here, we show that ectopic NSCs induced via bHLH transcription factor Deadpan (Dpn) expression fail to undergo appropriate temporal progression by constantly expressing mid‐temporal transcription factor(tTF), Sloppy‐paired 1/2 (Slp). Consequently, this resulted in impaired terminal differenation and generated an excess of Twin of eyeless (Toy)‐positive neurons at the expense of Reversed polarity (Repo)‐positive glial cells. Preference for a mid‐temporal fate in these ectopic NSCs is concordant with an enriched binding of Dpn at mid‐tTF loci and a depletion of Dpn binding at early‐ and late‐tTF loci. Retriggering the temporal series via manipulation of the temporal series or cell cycle is sufficient to reinstate neuronal diversity and timely termination.

## Graphical abstract

Dedifferentiated neural stem cells show aberrant expression of temporal transcription factors, resulting in incorrect types of neurons produced and impaired terminal differentiation. This has important ramifications for the utilisation of dedifferentiated stem cells for regenerative purposes.

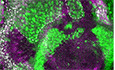

## Introduction

The central nervous system (CNS) is the cognitive control centre of the body and is generated by neural stem cells (NSCs) during development. *Drosophila* NSCs, called neuroblasts (NBs), exhibit key properties of mammalian NSCs such as self‐renewal, temporal control of proliferation and production of different neural subtypes that will form the adult brain. Thus, NBs serve as a powerful model to study the mechanisms that underlie the determination of overall brain size and neuronal diversity (Harding & White, 2018).

Molecular differences such as the mode of division and the temporal identity exist between different NB populations located in different parts of the brain. NBs within the ventral nerve cord (VNC) and the central brain (CB) of the larval CNS undergo Type I divisions, whereby the NB divides asymmetrically to generate a NB and a smaller ganglion mother cell (GMC). The GMC then divides to produce two daughter cells, which terminally differentiate into neurons or glia. A subset of NBs in the larval CB undergo Type II division, in which the NB's asymmetric division generates a NB and an intermediate neural progenitor (INP). Intermediate neural progenitors mature prior to dividing asymmetrically to produce another progenitor and a GMC. Within the optic lobes (OLs), the medulla outer proliferation centre (OPC) NBs are formed from a pseudostratified neuroepithelium through a differentiation wave characterised by proneural factor expression, termed the “proneural wave” (Egger *et al*, 2007; Yasugi *et al*, 2008). These NBs undergo asymmetric division and are broadly thought to behave similarly to Type I NBs.

In addition to positional identity, NBs can also be temporally defined by the expression pattern of sequential markers, known as the temporal series or temporal transcription factors (tTFs). The tTFs confer temporal identity to neurons born within each tTF window, and their cross‐regulation ensures unidirectional progression through the temporal series (Li *et al*, 2013; Suzuki *et al*, 2013). Type I, II and medulla NBs all express unique sets of tTFs; however, in all the regions of the CNS, tTFs play conserved roles to control neuronal diversity and proliferative potential of NBs (Maurange *et al*, 2008; Bayraktar & Doe, 2013; Li *et al*, 2013; Suzuki *et al*, 2013; Eroglu *et al*, 2014; Liu *et al*, 2015; Ren *et al*, 2017; Syed *et al*, 2017; Abdusselamoglu *et al*, 2019; Pahl *et al*, 2019; Konstantinides *et al*, 2022; Zhu *et al*, 2022).

One of the key regulatory mechanisms of brain size and neuronal diversity is the control of neuronal cell fate maintenance. We and others have shown that a number of transcription factors including Longitudinals Lacking (Lola), Midlife crisis and Nervous fingers 1 (Nerfin‐1) prevent neuron‐to‐NB reversion by repressing the expression of NB and cell‐cycle genes in post‐mitotic neurons in the OLs (Carney *et al*, 2013; Southall *et al*, 2014; Froldi *et al*, 2015, 2019; Xu *et al*, 2017; Vissers *et al*, 2018). Additionally, signalling pathways such as Notch can influence the differentiation status of neurons as expression of hyperactivated Notch can induce neuronal dedifferentiation and the formation of ectopic NBs (Xu *et al*, 2017; Vissers *et al*, 2018). However, the molecular mechanisms by which these factors regulate neuronal fate is not well‐understood. This is important for defining the logic by which NSCs mature into neurons and what prevents them from reverting into NSCs. Such knowledge is necessary for understanding normal CNS development, as well as in disease settings such as cancer. Furthermore, it could potentially be harnessed to promote regeneration. In this study, we identified the basic helix–loop–helix (bHLH) transcription factor, Deadpan (Dpn), as a mediator of dedifferentiation downstream of Nerfin‐1 in the OL. Overexpression of Dpn caused the formation of supernumerary NBs in deep neuronal layers of the medulla. The dedifferentiated NBs acquired and maintained an Eyeless (Ey)/Sloppy paired (Slp)‐positive mid‐temporal identity throughout larval neurogenesis, delaying the onset of the late‐tTF Tailess (Tll)‐positive fate. As a result, excessive Twin of eyeless (Toy)‐positive progeny was made at the expense of Tll‐positive neurons and Reversed polarity (Repo) glial cells. Furthermore, ectopic NBs generated via Dpn overexpression underwent slower cell cycle progression, and failed to terminally differentiate in a timely fashion. Mechanistically, our DamID analysis showed that Dpn binds preferentially to the mid‐temporal tTFs, where we observed a depletion of Dpn binding at early‐ and late‐tTF loci. Furthermore, retriggering the progression of the tTFs via Dichaete (D) was able to rescue both neuronal diversity and terminal differentiation. Similarly, promoting NB progression through G1/S phase of the cell cycle was able to restore temporal progression, neuronal diversity and terminal differentiation. Finally, we show that dedifferentiation induced by inhibition of Lola, Nerfin‐1, as well as hyperactivation of Notch in the OL similarly caused stalled temporal progression.

Together, our findings suggest that cell cycle regulators and temporal transcription factors are key determinants of proliferation and termination profiles of dedifferentiated NBs. To utilise dedifferentiated NSCs for regenerative purposes, we will have to recreate the correct temporal profiles and ensure cell cycle progression occurs appropriately.

## Results

### Deadpan overexpression induces ectopic neuroblasts in the optic lobe medulla region

**Figure 1:**
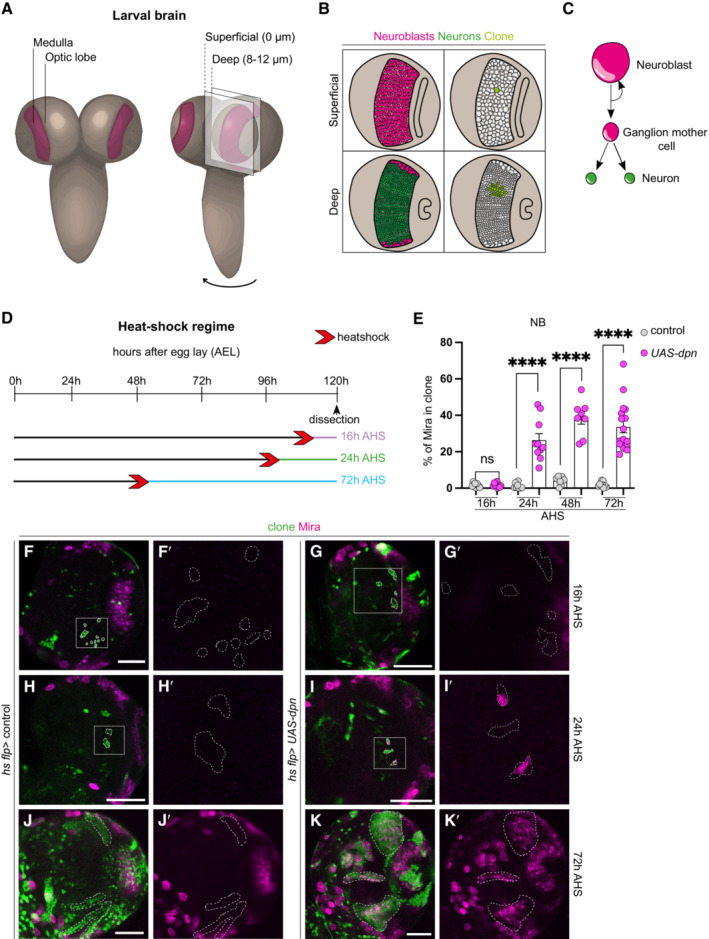
Dpn overexpression causes reversion of neurons to NBs in the deep layers of the developing CNS ASchematic representation of the larval central nervous system (CNS). Medulla (depicted in maroon) is located with the optic lobes (OLs) of the CNS. Cross section and cell composition at superficial surface (0 μm) and deep layer (8–12 μm) of the OLs are depicted in (B).B(left) Superficial surface is occupied by neuroblasts (NBs, pink), and deep layers are occupied by neurons (green). (right) Cross section through a *hs flp* clone (light green) showing the clone consists of a single NB in the superficial layer and its neuronal progeny in the deep layer.CSchematic representation of NB divisions: NBs undergo asymmetric division to give rise to a Ganglion mother cell (GMC) that produces two neurons.DSchematic depicting the heat shock regimes used in (E–K). Clones were induced via heat shock (red arrows), and dissected at 16 h (purple), 24 h (green), and 72 h (blue) after heat shock.EQuantification of % Mira^+^ cells in control and *UAS‐dpn* clones (calculated as the ratio of Mira^+^ cell volume as a percentage of total clone volume). Sixteen hours (Control *n* = 9, m = 1.584 ± 0.3779, *UAS‐dpn n* = 9, m = 1.657 ± 0.37), 24 h (Control *n* = 9, m = 1.150 ± 0.4781, *UAS‐dpn n* = 10, m = 26.37 ± 3.629), 48 h (Control *n* = 7, m = 3.971 ± 0.9249, *UAS‐dpn n* = 8, m = 38.56 ± 3.446), 72 h (Control *n* = 9, m = 1.874 ± 0.4897, *UAS‐dpn n* = 17, m = 33.62 ± 3.243).F–K′(F′, G′, H′, I′) are the magnified images of the square region outlined in (F, G, H, I). (F–K) are merged images, (F′–K′) are single channel images of (F–K) depicting the presence of NBs (Mira^+^). Clones (green) are outlined by dotted lines. All images in this figure, and all following figures are single confocal sections. (F–F′ and G–G′) No Mira^+^ NBs are recovered in deep sections of control or *UAS‐dpn* clones at 16 h after clone induction. Quantified in (E). (H–H′ and I–I′) At 24 h after clone induction, Mira^+^ NBs are recovered in *UAS‐dpn* but not control clones. Quantified in (E). (J–J′ and K–K′) At 72 h after clone induction, numerous Mira^+^ NBs are recovered in *UAS‐dpn* but not in control clones. Quantified in (E). Data information: Data are represented as mean ± SEM. *P*‐values were obtained using unpaired *t*‐test, and Welch's correction was applied in case of unequal variances. ****P < 0.0001. Scale bars: 50 μm.

**Figure EV1:**
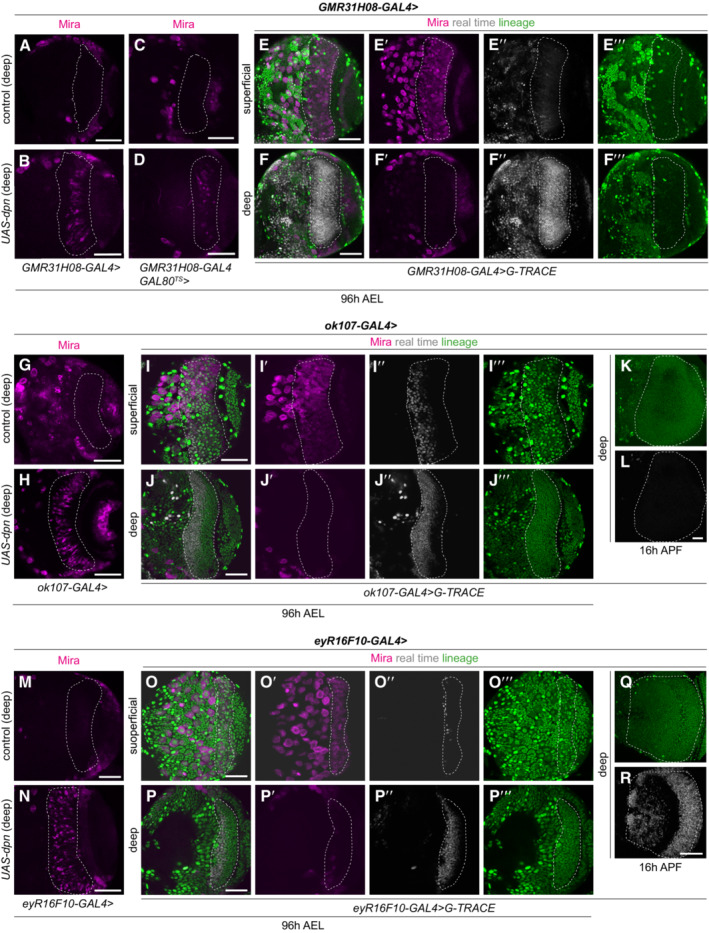
Lineage analysis of drivers utilised to induce dedifferentiation A–RRepresentative images of the deep medulla neuronal layer or neuroblast (NB) superficial layer in the larval optic lobe, in which *UAS‐dpn* and control are expressed using *GMR31H08‐GAL4*, *ok107‐GAL4*, and *eyR16F10‐GAL4* (outlined) and stained with the neuroblast (NB) marker, Miranda (Mira, magenta). (A–D) *GMR31H08‐GAL4* and *GMR31H08‐GAL4 GAL80* ^*TS*^ driving *UAS‐dpn* induces ectopic Mira^+^ cells in deep medulla layers compared to control. (E–F‴) *GMR31H08‐GAL4* is expressed in real time (grey) mainly in medulla neurons within the deep layer compared to NBs in the superficial layer. (G, H) *ok107‐GAL4* driving *UAS‐dpn* induces ectopic Mira^+^ cells in deep medulla layers compared to control. (I–L) *ok107‐GAL4* is expressed in real time (grey) in medulla neurons within the deep layer and NBs in the superficial layer 96 h AEL, however, its expression is not maintained at 16 h APF, therefore, this driver is unsuitable for examination of NB termination. (M, N) *eyR16F10‐GAL4* driving *UAS‐dpn* induces ectopic Mira^+^ cells in deep medulla layers compared to control. (O–R) *eyR16F10‐GAL4* is expressed in real time (grey) in medulla neurons within the deep layer and few NBs in the superficial layer 96 h AEL and is expressed at 16 h APF and is therefore a suitable driver for examination of NB termination. Scale bars: 50 μm.

We and others have previously shown that the Nerfin‐1 transcription factor maintains neuronal differentiation in Type I, II and medulla neuronal lineages (Froldi *et al*, 2015; Xu *et al*, 2017; Vissers *et al*, 2018). In the absence of Nerfin‐1, neurons undergo dedifferentiation to acquire a NB‐like fate, by turning on bona‐fide NB markers such as Deadpan (Dpn) and Miranda (Mira; Froldi *et al*, 2015; Xu *et al*, 2017; Vissers *et al*, 2018). In the OLs, symmetrically dividing neuroepithelial cells differentiate into medulla NBs located at the superficial CNS surface (0 μm, Fig 1A and B, pink), which in turn generates medulla neurons that occupy the deep layers of the CNS at around 8–12 μm from the superficial surface (Fig 1A–C, green; Egger *et al*, 2007; Yasugi *et al*, 2008). The pan‐neural bHLH transcription factor, Dpn, lies downstream of Notch signalling (San‐Juán & Baonza, 2011; San‐Juán *et al*, 2012) and was identified to be a Nerfin‐1 target gene in our previous DamID analyses (Vissers *et al*, 2018). To assess whether Dpn lies functionally downstream of Nerfin‐1, we tested whether *UAS‐dpn* caused neuronal dedifferentiation. Using three different neuronal/NB drivers (See Fig EV1 and methods for detailed expression analysis of the *GAL4s*) – *GMR31H08‐GAL4* (Jenett *et al*, 2012), *ey* ^*OK107*^ *‐GAL4* (Morante *et al*, 2011) and *eyR16F10‐GAL4* (Jenett *et al*, 2012), we found that Dpn overexpression in the medulla region of the brain induced the formation of ectopic NBs in deep sections of the CNS (Fig EV1A and B, G and H, and M and N, Vissers *et al*, 2018). Further confirmation of dedifferentiation was made through temporally restricted expression of Dpn during L3 using *GAL80ts;GMR31H08‐GAL4*. In this scenario, transgene expression in mature neurons also induced dedifferentiation (Fig EV1C and D). Finally, we confirmed that Dpn overexpression is capable of inducing neuronal dedifferentiation using clonal analysis (Fig 1D, via heat shock‐induced *actin‐Gal4 flp‐out*). Ectopic NBs (Mira^+^) were first recovered in clones 24 h after heat shock (AHS) in deep sections of the CNS (Fig 1F–I′, quantified in Fig 1E). This demonstrates that wild‐type NBs first generated mature neurons (indicated by the lack of ectopic NBs at 16 h AHS), which then underwent dedifferentiation to give rise to ectopic NBs by 24 h AHS. These ectopic NBs failed to express the neuronal marker Elav (Appendix Fig S1B–B″), and actively divided, as indicated by the M‐phase marker phospho‐Histone H3 (pH3, Appendix Fig S1A–A″). By 72 h AHS, clones that overexpressed Dpn possessed multiple Mira^+^ cells (Fig 1J and K).

As Dpn was identified to be a target of Nerfin‐1, we next investigated the epistatic relationship between Dpn and Nerfin‐1. We induced clones expressing Nerfin‐1 RNAi and compared the level of dedifferentiation with that of Nerfin‐1 RNAi clones additionally expressing Dpn RNAi. We found that Dpn RNAi expression significantly reduced the number of ectopic NBs induced by Nerfin‐1 knockdown, suggesting that Dpn lies downstream of Nerfin‐1 to mediate neuronal cell fate maintenance (Appendix Fig S1C–F).

### Ectopic neuroblasts generated via Dpn overexpression express mid‐temporal transcription factors

**Figure 2:**
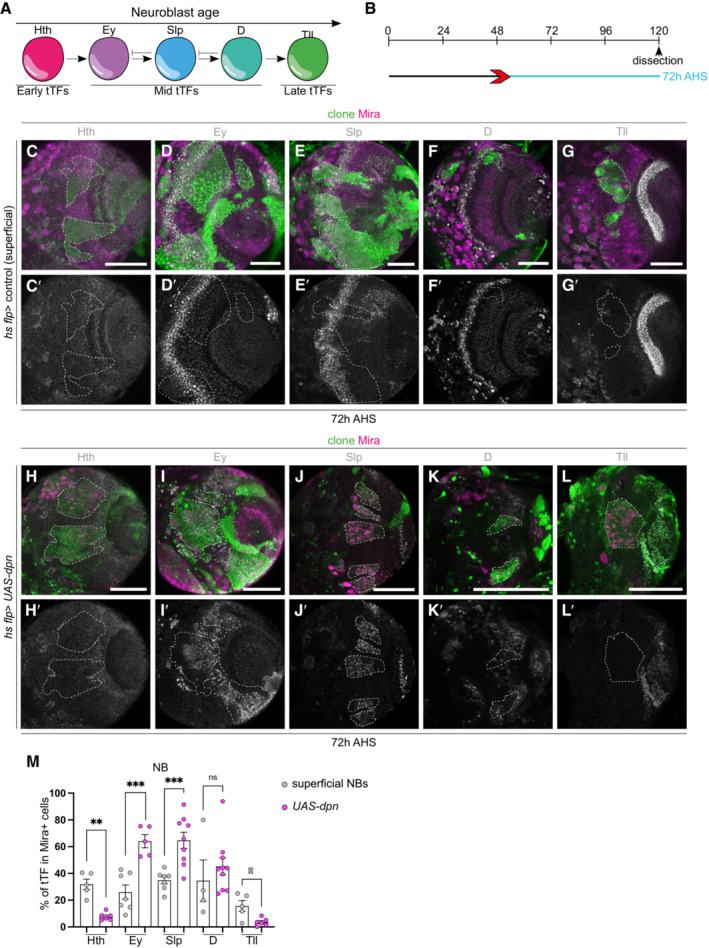
Ectopic NBs generated via *dpn* overexpression express mid‐temporal transcription factors ASchematic representation of temporal series in neuroblasts (NBs). NBs express temporal transcription factors (tTFs; Hth, Ey, Slp, D, Tll) as they age. These can be categorised into early, mid, and late tTFs.BSchematic depicting the heat shock regimes used in (C–L′). Clones were induced via heat shock (red arrows) and dissected at 72 h (blue) after heat shock.C–L′Representative images of the deep medulla neuronal layer or NB superficial layer in the larval optic lobe, in which *UAS‐dpn* or control are driven in clones by *hs flp* (marked by GFP and outlined) and stained with the stem cell marker, Miranda (Mira, magenta), and various temporal transcription factors (tTFs) (grey). (C–G′) In superficial sections of control clones, Mira^+^ superficial NBs express the tTF: Hth, Ey, Slp, D, Tll in concentric patterns. Quantified in (M). (H–L′) In deep sections of the NB clone induced via *UAS‐dpn*, Mira^+^ NBs do not express early tTF Hth or late‐tTF Tll, but are positive for mid‐tTFs Ey, Slp and D. Quantified in (M).MQuantification of volume of cells that express a specific tTF as % of total Mira^+^ NB volume within a clone. Hth (Control *n* = 5, m = 31.86 ± 3.89, *UAS‐dpn n* = 6, m = 7.779 ± 1.212), Ey (Control *n* = 7, m = 26.05 ± 5.302, *UAS‐dpn n* = 5, m = 64.08 ± 4.936), Slp (Control *n* = 7, m = 34.87 ± 2.753, *UAS‐dpn n* = 9, m = 64.72 ± 6.045), D (Control *n* = 4, m = 34.57 ± 15.49, *UAS‐dpn n* = 10, m = 45.2 ± 6.339), Tll (Control, *n* = 5, m = 15.65 ± 4.036, *UAS‐dpn n* = 6, m = 3.584, ±1.87). Data information: Data are represented as mean ± SEM. *P*‐values were obtained performing unpaired *t*‐test, and Welch's correction was applied in case of unequal variances. ***P < 0.001, **P < 0.005, *P < 0.05. Scale bars: 50 μm.

NBs contribute to the sequential generation of different neuron types by changing the expression of temporal transcription factors (tTFs) in a defined sequence (Apitz & Salecker, 2014). In the OLs, medulla NBs sequentially express a series of tTFs including the early factor Homothorax (Hth), mid‐tTFs Eyeless (Ey), Sloppy paired 1/2 (Slp) and Dichaete (D), and late‐tTF Tailless (Tll; Li *et al*, 2013; Suzuki *et al*, 2013). The tTFs contribute to the progression of the temporal series by activating the next tTF while repressing the previous tTF (Fig 2A). Medulla NBs continuously transit through the temporal cascade as they age; therefore, at any given time, different NBs in the superficial layer of the medulla would express Hth, Ey, Slp, D, Tll or a combination of these tTFs (Fig 2C–G′). As expected, 72 h after clone induction (Fig 2B), about 20–30% the superficial NBs were positive for either Hth, Ey, Slp, D or Tll (or a combination of these factors, Fig 2M). Surprisingly, the ectopic NBs in *UAS‐dpn* clones mostly expressed the mid‐tTFs Ey and Slp (Fig 2I–J′ and M), while some ectopic NBs expressed D and very few expressed the early‐tTF Hth or the late‐tTF Tll (Fig 2H–H′, K–L′ and M).

**Figure 3:**
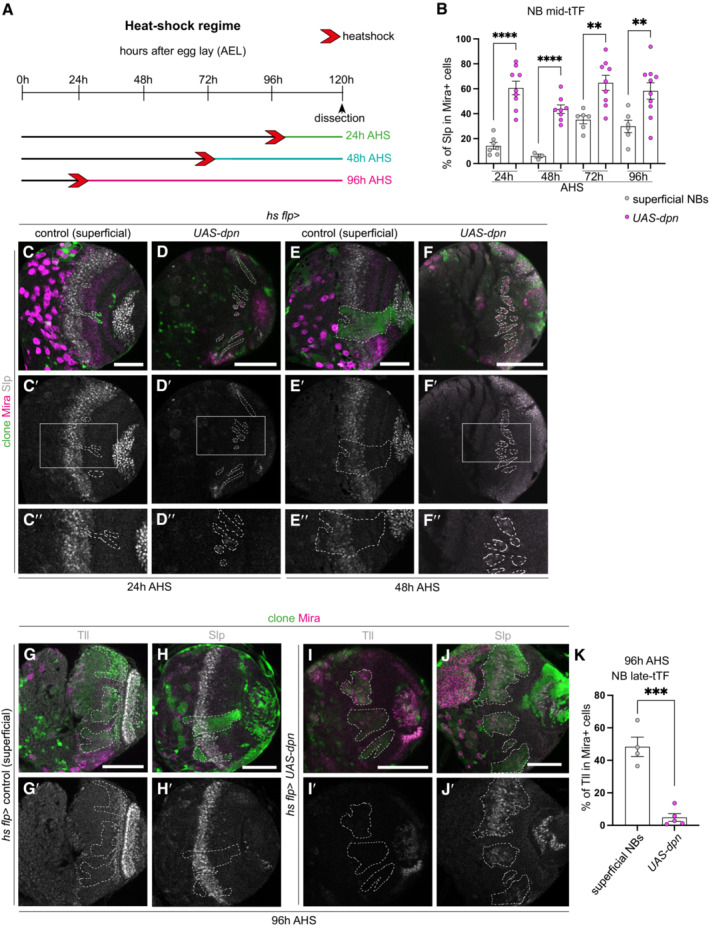
Ectopic NBs generated via *dpn* overexpression immediately adopt a mid‐temporal identity ASchematic depicting the heat shock regimes used in (B–F″) and (G–K′). Clones were induced via heat shock (red arrows) and dissected at 24 h (green), 48 h (turquoise), or 96 h (pink) after heat shock.BQuantification of % of Slp^+^ cells in Mira^+^ cells in control and *UAS‐dpn* clones (expressed as the ratio between Slp^+^ volume and total Mira^+^ volume within a clone). 24 h (Control *n* = 7, m = 14.25 ± 2.696, *UAS‐dpn n* = 9, m = 60.66 ± 5.384), 48 h (Control *n* = 3, m = 6.096 ± 1.509, *UAS‐dpn n* = 8, m = 43.64 ± 3.398), 72 h (Control *n* = 6, m = 35.13 ± 3.242, *UAS‐dpn n* = 9, m = 64.72 ± 6.045), 96 h (Control *n* = 6, m = 29.85 ± 4.951, *UAS‐dpn n* = 10, m = 58.2 ± 6.603).C–F″Representative images of the deep medulla neuronal layer in the larval optic lobe, in which *UAS‐dpn* or control are driven by *hs flp* (marked by GFP and outlined) and dissected and stained with the stem cell marker, Miranda (Mira, magenta), and Sloppy‐paired (Slp, grey). At 24 and 48 h after clone induction, Mira^+^ NBs express mid tTF Slp compared to superficial NB control, quantified in (B). (C″, D″, E″, F″) are the magnified images of the square region outlined in (C′, D′, E′, F′), respectively.G–J′At 96 h after clone induction, Mira^+^ NBs express mid tTF Slp and do not express late tTF Tll, compared to control, quantified in (B and K).KQuantification of % of Tll^+^ cells in Mira^+^ cells in control and *UAS‐dpn* clones (expressed as the ratio between Tll^+^ volume and total Mira^+^ volume within a clone). Control *n* = 4, m = 48.34 ± 5.956, *UAS‐dpn n* = 5, m = 4.805 ± 2.375. Data information: Data are represented as mean ± SEM. *P*‐values were obtained performing unpaired *t*‐test, and Welch's correction was applied in case of unequal variances. ****P < 0.0001, ***P < 0.001, **P < 0.005, *P < 0.05. Scale bars: 50 μm.

As NBs age, they are expected to undergo sequential transition through the sequence of Hth → Ey → Slp → D → Tll tTFs (Li *et al*, 2013; Suzuki *et al*, 2013). To assess whether ectopic NBs induced via Dpn overexpression also underwent sequential expression of these transcription factors, we induced *UAS‐dpn* clones, and examined their temporal profile at 24, 48, 72 and 96 h AHS (Fig 3A–F″, H–H′ and J–J′). At all time points examined, we found that between 40 and 70% of the dedifferentiated NBs expressed Slp (used as a proxy for midtemporal identity as it is specifically expressed in the NBs and not in neurons; Fig 3B). In addition, only 5% of the ectopic NBs generated via Dpn overexpression were positive for the late‐tTF Tll at 96 h AHS, compared to ~50% of control NBs at the same time (Fig 3G–K). Together, this indicates that ectopic NBs induced by Dpn overexpression adopt a mid‐temporal identity shortly after clone induction and maintain this identity throughout larval development.

### Neuroblasts generated via Dpn overexpression exhibit delayed terminal differentiation

**Figure 4:**
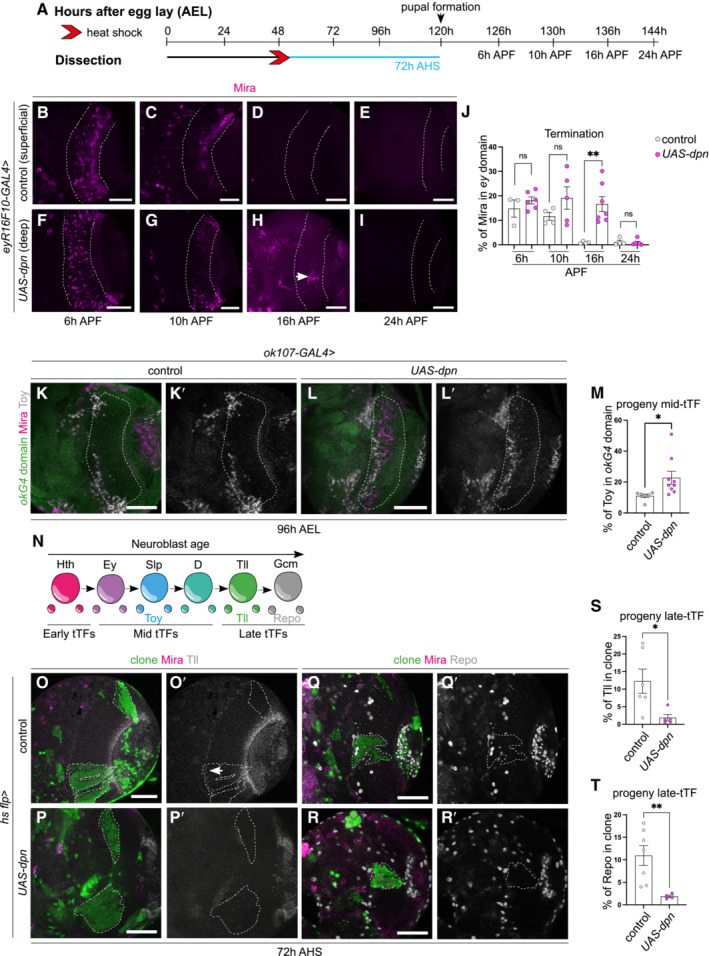
NBs generated via Dpn overexpression delays the timing of their terminal differentiation and generates Toy^+^ progeny at the expense of Tll^+^ neurons and Repo^+^ glial cells ASchematic depicting the timeline of experiments performed in (B–L′). Larval brains are dissected at 96 h, pupal brains are dissected at 126 h (6 h APF), 130 h (10 h APF), 136 h (16 h APF), and 144 h AEL (24 h APF). Heat shock regime for (Q–T′). Clones were induced by heat shock (red) at 48‐h AEL and dissected 72 h (blue) after heat shock.B–M(B–I, K–L′) Representative images of the superficial medulla neuroblast (NB) layer or deep medulla neuronal layer in the larval or pupal optic lobe, in which *UAS‐dpn* or control (*UAS‐luciferase*) are driven by *eyR16F10‐GAL4*, or *ok107‐GAL4* (outlined) and stained with the stem cell marker, Miranda (Mira, magenta), and mid‐temporal progeny marker, Toy‐GFP (grey). (B–E) Mira^+^ NBs are recovered in superficial sections of control under *eyR16F10‐GAL4* expression at 6 h APF and 10 h APF but not from16 h APF onwards, quantified in (J). (F–I) Mira^+^ NBs are recovered in deep sections of *UAS‐dpn* driven by *eyR16F10‐GAL4* at 6 h APF, 10 h APF, and 16 h APF but not at 24 h APF, quantified in (J). Arrow in (H) points to the NBs. (J) Quantification of % Mira^+^ cells within *eyR16F10‐GAL4* expression domain in control and *UAS‐dpn* (calculated as the ratio of Mira^+^ cell volume as a percentage of total *eyR16F10‐GAL4* domain volume). Six hours APF (Control, *n* = 3, m = 14.82 ± 3.521, *UAS‐dpn*, *n* = 6, m = 18.13 ± 1.46), 10 h APF (Control *n* = 4, m = 11.65 ± 1.605, *UAS‐dpn n* = 5, m = 19.11 ± 4.609), 16 h APF (Control *n* = 4, m = 0.8897 ± 0.239, *UAS‐dpn n* = 7, m = 16.64 ± 3.065), 24 h APF (Control *n* = 4, m = 1.174, ±0.7226, *UAS‐dpn n* = 6, m = 0.8518 ± 0.4849). (K–L′) More Toy+ progeny (grey) are present within the *ok107‐GAL4* domain (green, outlined) in *UAS‐dpn* compared to control. Quantified in (M). (M) Quantification of % Toy^+^ progeny within *ok107‐GAL4* expression domain in control and *UAS‐dpn* (calculated as the ratio of Toy^+^ cell volume as a percentage of total *ok107‐GAL4* domain volume). Control *n* = 6, m = 11.03 ± 1.169, *UAS‐dpn n* = 9, m = 22.78 ± 4.174.NSchematic representation of temporal series in NBs and their progeny. NBs express temporal transcription factors (tTFs; Hth, Ey, Slp, D, Tll) as they age. Slp^+^ NBs create Toy^+^ progeny; Tll^+^ NBs create Tll^+^ progeny; Gcm^+^ NBs create Repo^+^ progeny.O–R′Representative images of the deep medulla neuronal layer in the larval optic lobe, in which heat shock‐induced *UAS‐dpn* or control (*UAS‐luciferase*) clones are stained with the stem cell marker, Mira (magenta), late neuronal marker Tll (grey) or glial marker Repo (grey). Arrow points towards the small band of Tll^+^ neurons in control clones. (O–P′) At 72 h after clone induction, there is less Tll expression within *UAS‐dpn* clones than control clone, quantified in (S). (Q–R′) At 72 h after clone induction, there is less Repo expression within *UAS‐dpn* clones than control clones, quantified in (T).S, TQuantification of % Tll or Repo^+^ cells within control and *UAS‐dpn* clones (calculated as the ratio of Tll^+^ or Repo^+^ cell volume as a percentage of total clone volume). Tll (Control *n* = 6, m = 12.26 ± 3.449, *UAS‐dpn n* = 5, m = 1.881 ± 0.9293), Repo (Control *n* = 7, m = 10.94 ± 2.203, *UAS‐dpn n* = 4, m = 1.89 ± 0.2754). Data information: Data are represented as mean ± SEM. *P*‐values were obtained by unpaired *t*‐test, and Welch's correction was applied in case of unequal variances. **P < 0.005, *P < 0.05. Scale bars: 50 μm.

**Figure EV2:**
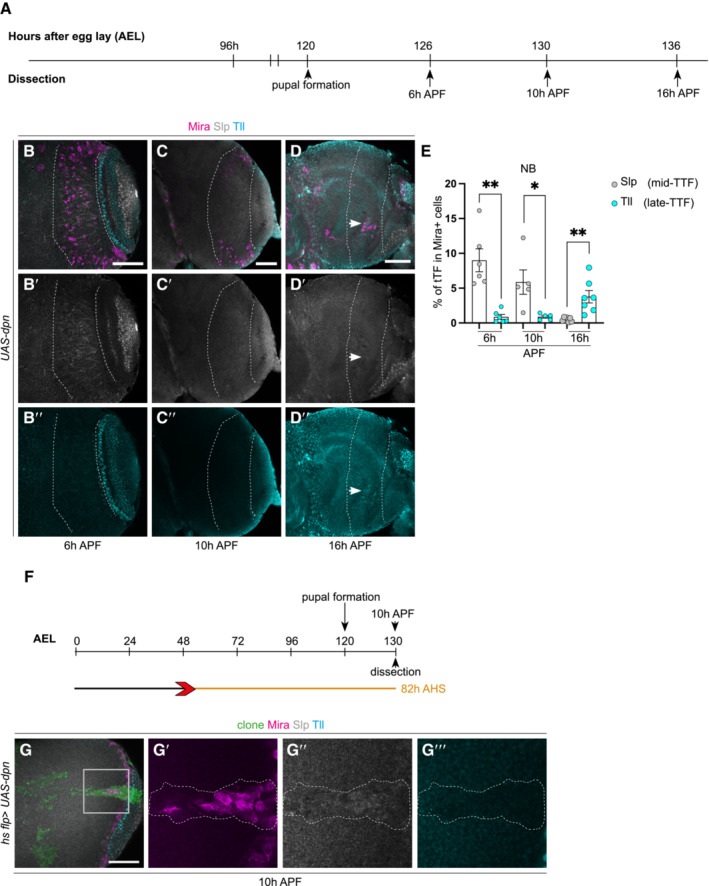
Slp and Tll expression during terminal differentiation in ectopic NBs generated via Dpn overexpression ATime‐line depicting age of ectopic neuroblasts (NBs). Pupal formation occurs at 120 h AEL; pupae are dissected at 126 h (6 h APF), 130 h (10 h APF), and 136 h (16 h APF).B–D″Representative images of the deep medulla neuronal layer in the pupal optic lobe, in which *UAS‐dpn* is driven by *eyR16F10‐GAL4* (outlined) and stained with the stem cell marker, Miranda (Mira, magenta), mid‐tTF, Sloppy‐paired (Slp, grey), and late tTF, Tailless (Tll, cyan). Dedifferentiated NBs express Slp and do not express Tll at 6 h APF and 10 h APF; however, they by 16 h APF they do not express Slp and instead express Tll. Quantified in (E).EQuantification of % Slp^+^ or Tll^+^ cells in Mira‐positive cells within the *eyR16F10‐GAL4* expression domain in *UAS‐dpn* (calculated as the ratio of Slp/Tll^+^ cell volume and Mira^+^ volume within *eyR16F10‐GAL4* domain). Six hours APF (Slp *n* = 6, m = 8.999 ± 1.64, Tll *n* = 6, m = 0.8718, ±0.3451). Ten hours APF (Slp *n* = 5, m = 5.876, ±1.752, Tll *n* = 5, m = 0.89 ± 0.1915). Sixteen hours APF (Slp *n* = 7, m = 0.4699, ±0.116, Tll *n* = 7, m = 3.767 ± 0.8893).FHeat shock regime for (G–G‴). Clones were induced by heat shock (red) at 48 h AEL and dissected at 126 h AEL (6 h APF), corresponding to 82 h (blue) hours after heat shock.G′–G‴Are magnified images of (G). At 82 h after clone induction (10 h APF), Mira^+^ NBs express Slp and not Tll. Data information: Data are represented as mean ± SEM. *P*‐values were obtained performing unpaired *t*‐test, and Welch's correction was applied in case of unequal variances. **P < 0.005, *P < 0.05. Scale bars: 50 μm.

Medulla NBs progress through the series of temporal factors until the oldest medulla NBs express Glial cells missing (Gcm) and transition from neurogenesis to gliogenesis and exit the cell cycle (Li *et al*, 2013; preprint: Zhu *et al*, 2021). Medulla NB cell cycle exit occurred around 16 h after pupal formation (APF) in control OLs (29°C, Fig 4A–D and J, no Mira^+^ NBs are recovered at this time point). When Dpn was overexpressed with *eyR16F10‐GAL4* (Jenett *et al*, 2012), which strongly drives gene expression in the pupal CNS (Fig EV1Q and R), significantly more NBs were recovered at 16 h, indicating that NBs termination was delayed (Fig 4H and J). *UAS‐dpn* NBs maintained the expression of the mid‐tTF Slp until at least 10 h APF (Fig EV2A–C″, E and F–G‴), but prior to their terminal differentiation they began to reduce Slp expression and expressed the late‐tTF Tll (Fig EV2D and E). These data suggest that ectopic NBs generated via Dpn overexpression exhibit a prolonged mid‐temporal identity before eventually switching on late‐tTFs, and undergoing terminal differentiation and exiting the cell cycle, as no NBs were detected by 24 h APF (Fig 4E–J).

### Dpn overexpression induces neuroblasts to generate Toy^+^ progeny at the expense of Tll^+^ neurons and Repo^+^ glial cells

Hth, Ey, Slp, D and Tll are each required for the sequential generation of different medulla neurons through the production of Brain‐specific homeobox (Bsh), Drifter (Dfr), Toy/Sox102F, Ets at 65A (Ets65a) and Tll, respectively (Morante & Desplan, 2008; Hasegawa *et al*, 2011; Li *et al*, 2013; Suzuki *et al*, 2013; Fig 4N). We next assessed the neuronal subtypes made by Dpn‐induced ectopic NBs. To better assess the cumulative effect of the neurons that are made throughout CNS development, *ey* ^*OK107*^ *‐GAL4* was used to drive the expression of Dpn. About 50% more Toy^+^ progeny were made by Dpn‐induced ectopic NBs than by control NBs (Fig 4K–M). Consistent with this, *UAS‐dpn* clones produced very few Tll^+^ neurons or Repo^+^ glial cells (Fig 4O–T). This indicates that Dpn overexpression in the medulla stalls the temporal progression of NBs, causing them to produce an excess of Toy^+^ progeny at the expense of Tll^+^ neurons and Repo^+^ glial cells.

### Genome‐wide profiling of Dpn binding reveals a bias towards mid‐temporal identity genes in medulla neuroblasts

**Figure 5:**
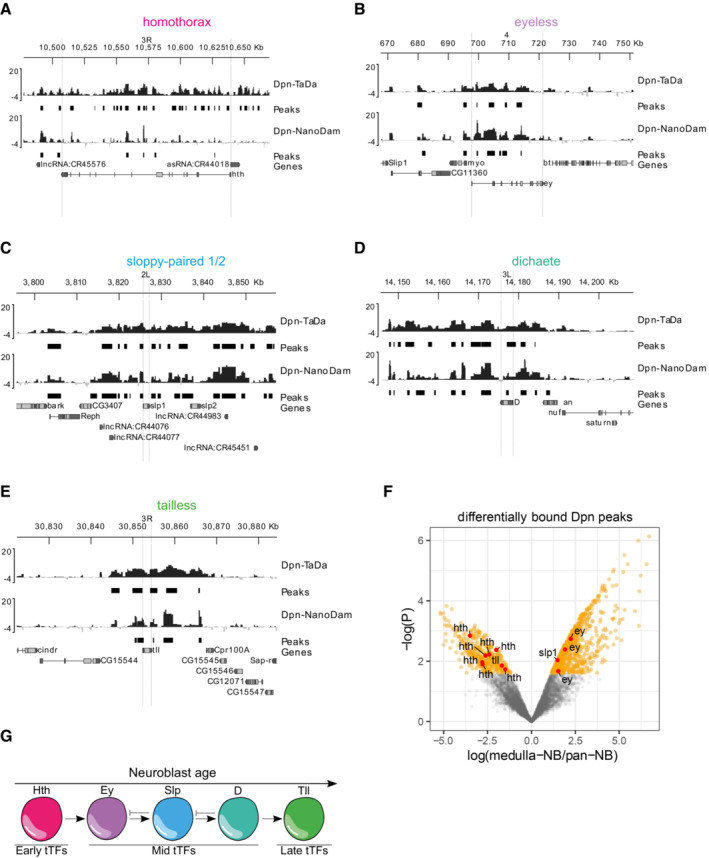
Dpn binds to tTFs with preference to mid‐tTFs A–EMean Targeted DamID Dpn (pan‐neuroblast, NB) and Dpn‐NanoDam (medulla specific) binding profiles (log2(Dam‐fusion/Dam) are shown for the optic lobe temporal TFs (A) homothorax (B) eyeless (C) sloppy‐paired 1/2 (D) dichaete and (E) tailess. Peaks with FDR < 0.01 are shown.FVolcano plot of differentially‐bound Dpn peaks in the medulla show that Dpn preferentially bind preferentially to mid‐tTFs Slp1/2 and Ey, but not to early or late tTFs Hth or Tll.GSchematic representation of temporal series in NBs. NBs express temporal transcription factors (tTFs; Hth, Ey, Slp, D, Tll) as they age.

**Figure EV3:**
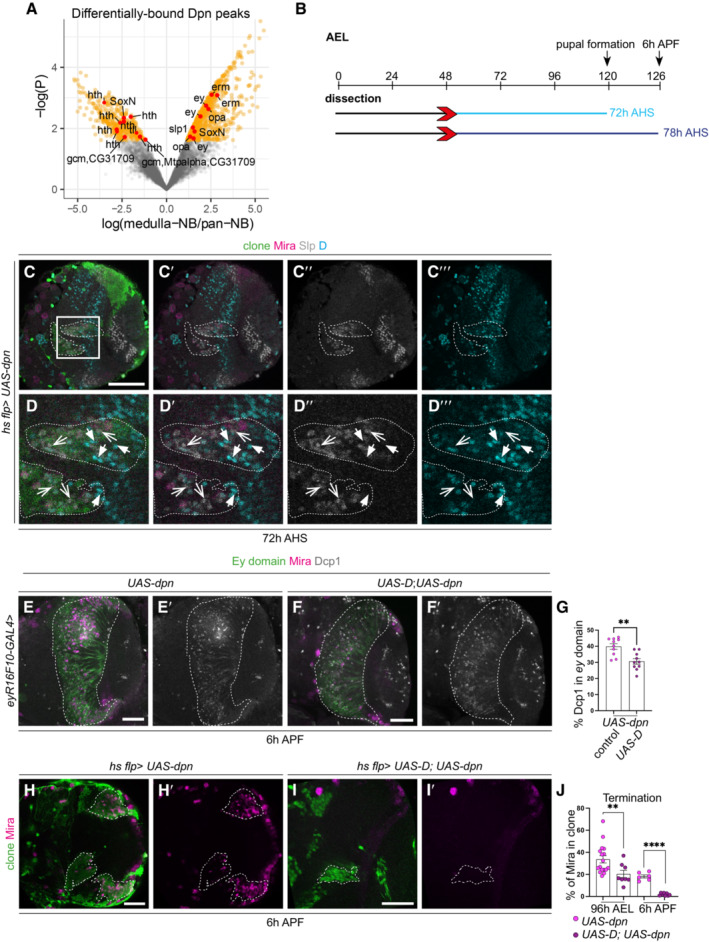
Slp and D are expressed in complementary patterns and *UAS‐D* promotes premature termination of *UAS‐dpn* ectopic NBs AVolcano plot of differentially bound by Dpn in the medulla. The temporal transcription factors (tTFs) depicted here include the more comprehensive network of transcription factors from Zhu *et al* (2022).BSchematic depicting the heat shock regimes used in (C–D‴ and H–I′). Clones were induced via heat shock (red arrows) at 48 h and dissected 72 h (light blue) or 78 h (6 h APF, dark blue) after heat shock.C–D‴Representative images of the deep medulla neuronal layer in the larval optic lobe, where *UAS‐dpn* generated by *hs flp*, express complementary patterns of Slp and D expression. (D–D‴) are magnified of (C–C‴). At 72 h after clone induction, Mira^+^neuroblasts (NBs) express Slp or D. Open arrow heads represent NBs that express Slp and not D. Closed arrow heads represent NBs that express D and not Slp. Miranda (Mira, magenta), D (cyan), Slp (grey).E–F′Representative images of the deep medulla neuronal layer in the larval optic lobe, where less cell death as detected by Death caspase 1 (Dcp1, grey) is found in ectopic NBs (Mira (magenta)) induced via *UAS‐D*; *UAS‐dpn* compared to *UAS‐dpn* (driven by *eyR16F10‐GAL4*). Quantified in (G).GQuantification of % Dcp1^+^ cells within the *eyR16F10‐GAL4* expression domain in the deep section of *UAS‐dpn* or *UAS‐D*; *UAS‐dpn* (calculated as the ratio of Dcp1^+^ cell volume within *eyR16F10‐GAL4* domain). *UAS‐dpn n* = 10, m = 39.87 ± 1.720, *UAS‐D*; *UAS‐dpn n* = 10, m = 30.59 ± 1.715.H–I′Representative images of the deep medulla neuronal layer in the larval optic lobe, where *UAS‐dpn* or *UAS‐D; UAS‐dpn* clones are induced via *hs flp*. NBs are marked by stem cell marker, Mira (magenta). At 96 h AEL and 6 h APF, NBs underwent premature terminal differentiation upon overexpression of (D), quantified in (J).JQuantification of % Mira^+^ cells in control *UAS‐dpn* and *UAS‐D; UAS‐dpn* clones (calculated as the ratio of Mira^+^ cell volume as a percentage of total clone volume). 72 h (*UAS‐dpn n* = 17, m = 33.62 ± 3.243, *UAS‐D; UAS‐dpn n* = 8, m = 20.38 ± 3.307). 78 h (*UAS‐dpn n* = 6, m = 18.3 ± 1.46, *UAS‐D; UAS‐dpn n* = 8, m = 1.859 ± 0.4215). Data information: Data are represented as mean ± SEM. *P*‐values were obtained performing unpaired *t*‐test, and Welch correction was applied in case of unequal variances. ****P < 0.0001, **P < 0.005. Scale bars: 50 μm.

**Figure EV4:**
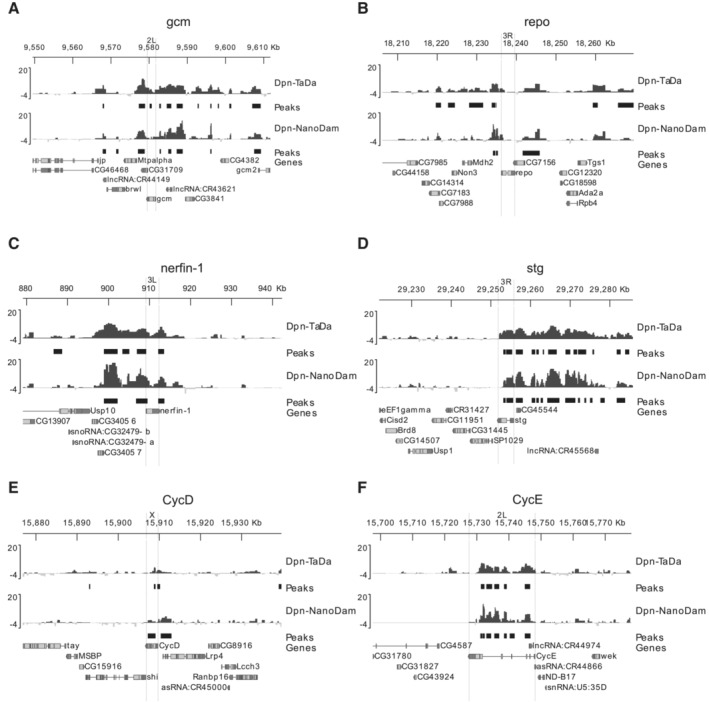
Dpn binds to tTFs and cell cycle loci A–FMean Targeted DamID and Dpn‐NanoDam Dpn binding profiles (log2(Dam‐fusion/Dam)) are shown for the optic lobe temporal TFs (A) gcm, (B) repo, (C) nerfin‐1; and for the cell cycle genes (D) stg, (E) CycD and (F) CycE. peaks with FDR < 0.01 are shown.

To understand why NBs dedifferentiated from medulla neurons specifically adopt a mid‐temporal fate, we profiled the genome‐wide binding of Dpn in 3^rd^ instar larval NBs using Targeted DamID (TaDa; Southall *et al*, 2013; Marshall *et al*, 2016). Using the NB‐specific *wor‐GAL4* driver line, and a Dpn‐Dam profiling line generated via FlyORF‐TaDa (Aughey *et al*, 2021), we profiled the binding sites of Dpn in all larval NBs. We then compared this to the binding of Dpn profiled only in medulla NBs, obtained using the NanoDam technique (Tang *et al*, 2022), a BAC‐recombineered Dpn‐GFP line under endogenous control, and the medulla‐specific *eyR16F10‐GAL4* driver line. We found that although Dpn bound in proximity to all five temporal cascade TFs in all NBs (Fig 5A–E and G), the binding of Dpn was lost or significantly reduced at *hth* and *tll* in medulla NBs, and significantly increased at *ey* and *slp1* (Fig 5F). Extending this analysis to the recently described complete medulla temporal patterning TF network (Zhu *et al*, 2022) showed a significant enrichment of Dpn binding at almost all mid‐temporal identity TFs, and a significant depletion at early and late tTFs (Figs EV3A and EV4A–C). Collectively, our genomics and genetic data suggest that the mid‐temporal identity tTFs are direct targets of Dpn in the medulla and are activated upon Dpn overexpression.

### Dichaete triggers the re‐initiation of the temporal series

**Figure 6:**
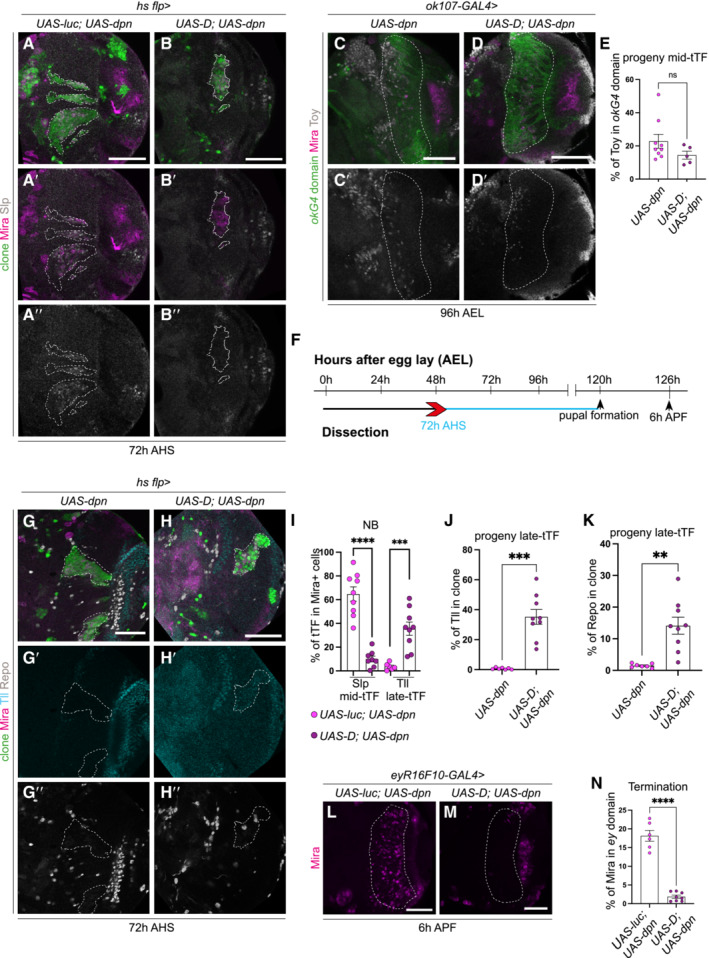
Dichaete triggers the re‐initiation of the temporal series A–H″(A–B″ and G–H″) Representative images of deep medulla neuronal layer in the larval optic lobe, in which *UAS‐luc*; *UAS‐dpn* and *UAS‐D; UAS‐dpn* are expressed in clones by *hs flp*, and stained with the stem cell marker, Miranda (Mira, magenta), and/or Slp, Tll or Repo (grey). (A–B″) At 72 h after clone induction, Mira^+^neuroblasts (NBs) within *UAS‐luc; UAS‐dpn* clones express Slp; Mira^+^ NBs within *UAS‐D; UAS‐dpn* clones do not express Slp, quantified in (I). (C–D′) Representative images of deep medulla neuronal layer in the larval optic lobe, in which *UAS‐dpn* and *UAS‐D; UAS‐dpn* are expressed in the *ok107‐GAL4* expression domain, and stained with the stem cell marker, Mira (magenta), and mid‐temporal progeny marker, Toy‐GFP (grey). Fewer Toy^+^ cells were recovered within the *ok107‐GAL4* domain (outlined) in *UAS‐D; UAS‐dpn* compared to *UAS‐dpn*, quantified in (E). (E) Quantification of % Toy^+^ cells within *ok107‐GAL4* expression domain in *UAS‐dpn* and *UAS‐D; UAS‐dpn* (calculated as the ratio of Toy^+^ cell volume as a percentage of total *ok107‐GAL4* domain volume). *UAS‐dpn n* = 9, m = 22.78 ± 4.174, *UAS‐D; UAS‐dpn n* = 5, m = 14.42 ± 2.394. (F) Timeline depicting the age of ectopic neuroblasts. Pupal formation occurs at 120 h AEL. Pupae are dissected at 126‐h AEL (6‐h APF). Heat shock regime for (A–B″) and (G–H″). Clones were induced by heat shock (red) at 48 h AEL and dissected at 72‐h (blue) after heat shock. (G–H″) At 72 h after clone induction, more Tll^+^ and Repo^+^ cells were recovered within *UAS‐D; UAS‐dpn* clones compared to *UAS‐dpn* clones, quantified in (I–K).IQuantification of % of Slp^+^ or Tll^+^ cells in Mira^+^ cells *UAS‐luc; UAS‐dpn* clones compared to *UAS‐D; UAS‐dpn* clones (expressed as the ratio between tTF^+^ volume and total Mira^+^ volume within a clone). Slp (*UAS‐luc; UAS‐dpn n* = 9, m = 64.72 ± 6.045, *UAS‐D; UAS‐dpn n* = 8, m = 9.99 ± 2.463), Tll (*UAS‐luc; UAS‐dpn n* = 6, m = 3.584 ± 1.187, *UAS‐D; UAS‐dpn n* = 9, m = 35.53 ± 5.596).J, KQuantification of % Tll^+^ or Repo^+^ cells within *UAS‐dpn* clones compared with *UAS‐D; UAS‐dpn* clones (calculated as the ratio of Tll^+^ or Repo^+^ cell volume as a percentage of total clone volume). Tll (*UAS‐dpn n* = 7, m = 0.7198 ± 0.2389, *UAS‐D; UAS‐dpn n* = 9, m = 35.22 ± 4.917), Repo (*UAS‐dpn n* = 7, m = 1.488 ± 0.2368, *UAS‐D; UAS‐dpn n* = 9, m = 14.13 ± 2.672).L, MMira^+^ NBs are present in deep sections of *UAS‐dpn* driven by *eyR16F10‐GAL4* at 126 h but are absent in *UAS‐D; UAS‐dpn*, quantified in (N).NQuantification of % Mira cells within *eyR16F10‐GAL4* expression domain in *UAS‐dpn* and *UAS‐D; UAS‐dpn* (calculated as the ratio of Mira^+^ cell volume as a percentage of total *eyR16F10‐GAL4* domain volume). *UAS‐dpn n* = 6, m = 18.13 ± 1.46, *UAS‐D; UAS‐dpn n* = 8, m = 1.859 ± 0.4215. Data information: Data are represented as mean ± SEM. *P*‐values were obtained via unpaired *t*‐test, and Welch's correction was applied in cases of unequal variances. ****P < 0.0001, ***P < 0.001, **P < 0.005. Scale bars: 50 μm.

The mid‐temporal identity tTFs form a mutually exclusive regulatory network, where the expression of one inhibits the others (Li *et al*, 2013; Suzuki *et al*, 2013; Zhu *et al*, 2022). Consistent with this, we found that D and Slp were expressed in a complementary pattern in *UAS‐Dpn* clones, where high expression of D correlated with low expression of Slp (Fig EV3B–D‴, closed arrows), and vice versa (Fig EV3B–D‴, open arrows). Next, we investigated whether inhibiting Slp expression via overexpression of D was sufficient to promote temporal progression in stalled Dpn‐overexpressing NBs. Overexpression of D in *UAS‐dpn* clones caused a significant reduction in Slp expression, and a corresponding increase in Tll expression in NBs, indicating that D can promote NB temporal progression towards a late temporal identity (Fig 6A–B″, F and I). This also induced an increase in the production of Tll^+^ neurons and Repo^+^ glial cells (Fig 6G–H″, J and K), indicating that D promoted NB temporal progression towards a late temporal fate. While a reduction in Toy^+^ progeny was also observed (Fig 6C–E′), this reduction was not significant, as neurons produced by D^+^ NBs can also express Toy (Bertet *et al*, 2014). Consistent with the model that D causes premature termination of the neurogenesis programme, we found that D overexpression in *UAS‐dpn* NBs did not increase cell death (Fig EV3E–G), but an earlier cessation of neurogenesis, as indicated by a reduction in Mira^+^ cells at 6 h APF (*eyR16F10‐GAL4* (Fig 6L–N), flp‐out clones (Fig EV3H–J)).

### Temporal progression of dedifferentiated neuroblasts is regulated by the cell cycle

**Figure 7:**
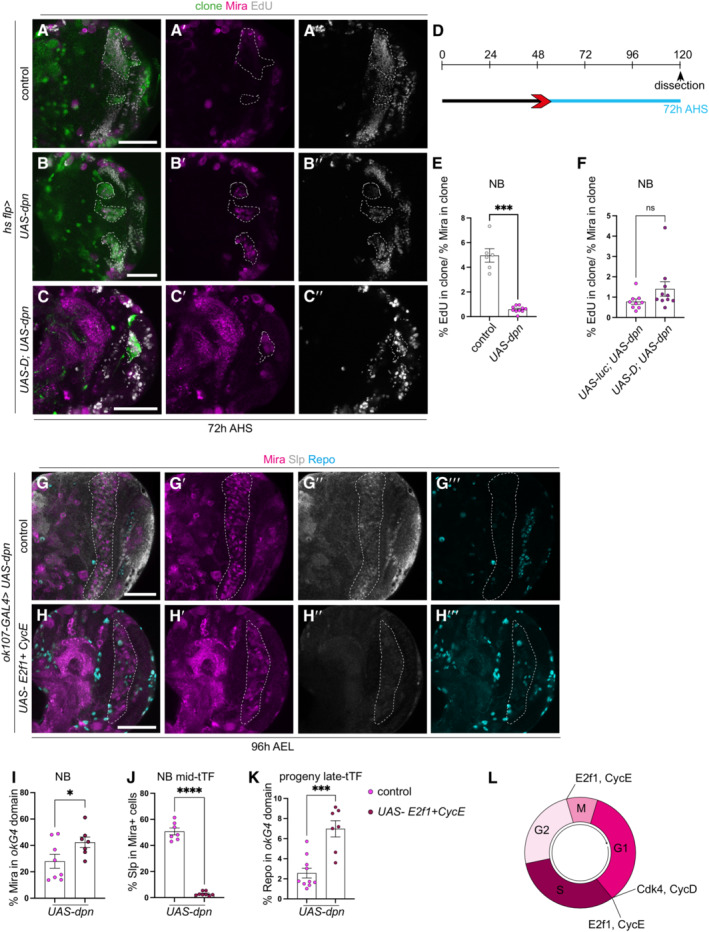
Temporal progression of dedifferentiated NBs is regulated by the cell cycle A–C″Representative images of the deep medulla neuronal layer in the larval optic lobe, in which control (*UAS‐luc*) and *UAS‐dpn* and *UAS‐D*; *UAS‐dpn* are expressed in *hs flp* clones (72 h after clone induction). Miranda (Mira, magenta) and EdU (grey). Despite increased number of Mira^+^ cells in *UAS‐dpn* compared to control, each neuroblast (NB) underwent slower cell cycle progression, as indicated by EdU^+^ incorporation. Quantified in (E). This slow cell cycle speed was not rescued in *UAS‐D; UAS‐dpn* clones. Quantified with *UAS‐luc*; *UAS‐dpn* in (F).DHeat shock regime for (A–C″). Clones were induced by heat shock (red) at 48‐h AEL and dissected 72‐h (blue) after heat shock.EQuantification of % of EdU‐positive cells in control (*UAS‐luc*) and *UAS‐dpn* clones (expressed as the amount of EdU^+^ cells per Mira^+^ volume within the clone). Control *n* = 6, m = 4.96 ± 0.5433, *UAS‐dpn n* = 10, m = 0.5927 ± 0.08484.FQuantification of % of EdU‐positive cells in *UAS‐luc; UAS‐dpn* and *UAS‐D; UAS‐dpn* clones (expressed as the amount of EdU^+^ cells per Mira^+^ volume within the clone). *UAS‐luc; UAS‐dpn n* = 9, m = 0.7807 ± 0.1311, *UAS‐D; UAS‐dpn n* = 10, m = 0.4866 ± 0.3579.G–H‴Representative images of the deep medulla neuronal layer in the larval optic lobe, where *UAS‐ E2f1; UAS‐ CycE*; *UAS‐dpn* or *UAS‐dpn* are expressed in the *ok107‐GAL4* domain (outlined), and stained with the stem cell marker, Mira (magenta), mid‐tTF, Slp (grey) and late progeny marker, Repo (Cyan). Quantified in (I–K). *UAS‐dpn* NBs express Slp and do not create Repo^+^ progeny, whereas *UAS‐ E2f1*; *UAS‐ CycE*; *UAS‐dpn* NBs do not express Slp and create Repo^+^ progeny.IQuantification of % Mira^+^ cells within *ok107‐GAL4* expression domain in *UAS‐dpn* control and *UAS‐ E2f1*; *UAS‐ CycE*; *UAS‐dpn* (calculated as the ratio of Mira^+^ cell volume as a percentage of total *ok107‐GAL4* volume). *UAS‐dpn* control *n* = 8, m = 27.99 ± 5.288, *UAS‐ E2f1*; *UAS‐ CycE*; *UAS‐dpn n* = 7, m = 42.37 ± 3.999.JQuantification of % Slp^+^ cells in Mira‐positive cells within the *ok107‐GAL4* expression domain in *UAS‐dpn*; *UAS luc* and *UAS‐E2f1*; *UAS‐CycE*; *UAS‐dpn* (calculated as the ratio of Slp^+^ cellular volume as a percentage of total Mira^+^ volume within *ok107‐GAL4* domain). *UAS‐dpn* control *n* = 7, m = 50.76 ± 2.582, *UAS‐E2f1*; *UAS‐CycE*; *UAS‐dpn n* = 8, m = 2.907 ± 0.5755.KQuantification of % Repo^+^ progeny in within the *ok107‐GAL4* expression domain in *UAS‐dpn*; *UAS luc* and *UAS‐E2f1*; *UAS‐CycE*; *UAS‐dpn* (calculated as the ratio of Repo^+^ cellular volume within *ok107‐GAL4* domain). *UAS‐dpn* control *n* = 10, m = 2.567 ± 0.491, *UAS‐E2f1*; *UAS‐CycE*; *UAS‐dpn n* = 7, m = 6.977 ± 0.8049.LSchematic representation of the cell cycle. Cdk4/CycD and E2f/CycE play roles in G1/S phase transition. E2f/ CycE play a role in G1/S and G2/M phase transitions. Data information: Data are represented as mean ± SEM. *P*‐values were obtained performing unpaired *t*‐test, and Welch's correction was applied in case of unequal variances. ****P < 0.0001, ***P < 0.001, **P < 0.005, *P < 0.05. Scale bars: 50 μm.

Cell division is essential for neurogenesis, and the progression through the cell cycle is important for temporal transition in Type I NBs (van den Ameele & Brand, 2019). We performed an EdU pulse chase experiment, which showed that *UAS‐dpn* clones produced significantly fewer progeny compared with control clones (the number of EdU^+^ cells was normalised to the number of NBs), suggesting that the pace of the cell cycle was slower in dedifferentiated NBs compared with wild‐type NBs (Fig 7A–B″, D and E). In addition, our DamID analysis showed that the cell cycle genes *cyclin D* (*cycD*), cyclin E (*cycE*) and *string* (*stg*) are direct targets of Dpn (Fig EV4D–F). Therefore, it is possible that Dpn can directly inhibit cell cycle progression, which in turn prevents temporal transition. To test this hypothesis, we overexpressed the key cell cycle regulators E2F transcription factor 1 (E2f1) and CycE, which can promote cell cycle progression at both the G2/M and G1/S check points (Fig 7L). This manipulation induced a small increase in Mira^+^ NBs (Fig 7G–I) and was able to promote temporal progression in Dpn‐expressing NBs, as indicated by fewer Slp^+^ NBs and a significant increase in Repo^+^ glial cells (Fig 7G–H‴, J and K).

Interestingly, overexpression of CycD and Cyclin‐dependent kinase 4 (Cdk4), which promotes G1/S progression (Fig 7L), had an even stronger effect on temporal transition, resulting in premature neurogenesis (Appendix Fig S2B–D) to gliogenesis (Appendix Fig S2E–G) at 96h AEL. As both D and cell cycle genes can promote temporal progression in *UAS‐dpn* clones, we next assessed whether *UAS‐D* also affected cell cycle progression in *UAS‐dpn* clones. By performing an EdU pulse chase assay, we found that D overexpression did not significantly increase the rate of neuronal production in dedifferentiated NBs (Fig 7C–C″ and F). Together, this data suggest that in the OL, cell cycle progression lies upstream of the temporal series, to promote the generation of neurons.

### Notch, Lola and Nerfin‐1‐mediated dedifferentiation also causes temporal series progression defects

**Figure 8:**
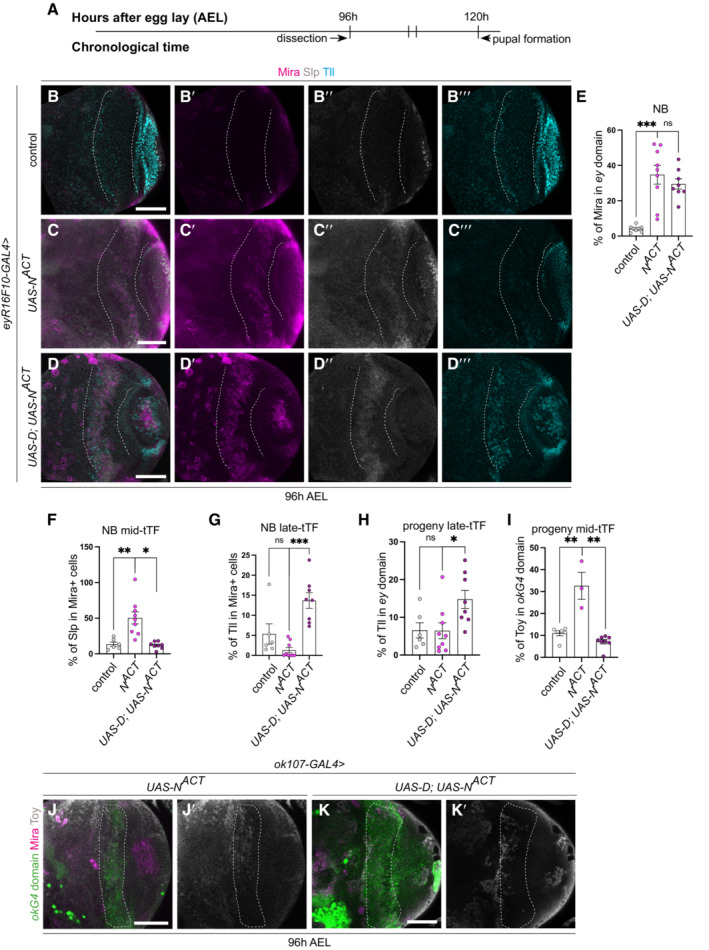
Dichaete re‐initiates the temporal series in dedifferentiated NBs caused by Notch activation ATimeline depicting age of ectopic neuroblasts (NBs). Larvae are dissected at 96 h AEL.B–D‴Representative images of the deep medulla neuronal layer in the larval optic lobe, in which control (*UAS‐luc*), *UAS‐N* ^*ACT*^, and *UAS‐D; UAS‐N* ^*ACT*^ are driven by *eyR16F10‐GAL4* (outlined). Miranda (Mira, magenta), Sloppy paired (Slp, grey), Tailless (Tll, cyan). *UAS‐N* ^*ACT*^ NBs express Slp and not Tll. *UAS‐D; UAS‐N* ^*ACT*^ NBs express Slp and Tll, quantified in (E–H).EQuantification of % Mira‐positive cells within *eyR16F10‐GAL4* expression domain in control, *UAS‐N* ^*ACT*^, and *UAS‐D; UAS‐N* ^*ACT*^ (calculated as the ratio of Mira^+^ cell volume as a percentage of total volume of *eyR16F10‐GAL4* domain). Control *n* = 6, m = 4.077, ±2.103, *UAS‐N* ^*ACT*^ *n* = 9, m = 34.71, ±5.271, *UAS‐D*; *UAS‐N* ^*ACT*^ *n* = 8, m = 29.5, ±2.931.F, GQuantification of % Slp^+^ or Tll^+^ cells in Mira‐positive cells within the *eyR16F10‐GAL4* expression domain in control, *UAS‐N* ^*ACT*^, and *UAS‐D; UAS‐N* ^*ACT*^ (calculated as % of Slp^+^ or Tll^+^ cellular volume that are also Mira^+^ within *eyR16F10‐GAL4* domain). Slp (Control *n* = 6, m = 13.62 ± 2.974, *UAS‐N* ^*ACT*^ *n* = 9, m = 13.62 ± 2.974, *UAS‐D*; *UAS‐N* ^*ACT*^ *n* = 8, m = 11.98 ± 1.741), Tll (Control *n* = 6, m = 5.363 ± 2.531, *UAS‐N* ^*ACT*^ *n* = 9, m = 1.316 ± 0.643, *UAS‐D*; *UAS‐N* ^*ACT*^ *n* = 8, m = 13.71 ± 1.955).HQuantification of % Tll^+^ cells within *eyR16F10‐GAL4* expression domain in control,*UAS‐N* ^*ACT*^, and *UAS‐D; UAS‐N* ^*ACT*^ (calculated as the ratio of Tll^+^ cellular volume as a percentage of total volume of *eyR16F10‐GAL4* domain). Control *n* = 6, m = 6.554 ± 2.018, *UAS‐N* ^*ACT*^ *n* = 9, m = 6.449 ± 2.099, *UAS‐D*; *UAS‐N* ^*ACT*^ *n* = 8, m = 14.77 ± 2.404.IQuantification of % Toy^+^ cells within *ok107‐GAL4* expression domain in control (*UAS‐luc*), *UAS‐N* ^*ACT*^ and *UAS‐ N* ^*ACT*^ *; UAS‐dpn* (calculated as the ratio of Toy^+^ cell volume as a percentage of total *ok107‐GAL4* domain volume). The column for Control uses the same data points as in Fig 3K. Control *n* = 6, m = 11.03 ± 1.169, *UAS‐N* ^*ACT*^ *n* = 3, m = 32.65 ± 6.176, *UAS‐N* ^*ACT*^ *; UAS‐dpn n* = 8, m = 7.178 ± 1.023).J–K′Representative images of the deep medulla neuronal layer in the larval optic lobe, in which *UAS‐N* ^*ACT*^ and *UAS‐N* ^*ACT*^ *; UAS‐dpn* are driven by *ok107‐GAL4* (marked by GFP and outlined) and stained with the stem cell marker, Mira (magenta), and mid‐temporal progeny marker, Toy‐GFP (grey). Fewer cells express Toy^+^ in *UAS‐N* ^*ACT*^ *; UAS‐dpn* compared to *UAS‐N* ^*ACT*^, quantified in (I). Data information: Data are represented as mean ± SEM. *P*‐values were obtained performing unpaired *t*‐test, and Welch's correction was applied in case of unequal variances. ****P < 0.0001, ***P < 0.001, **P < 0.005. Scale bars: 50 μm.

To determine whether dedifferentiated NBs commonly exhibit defects in their temporal identity, we next examined other models of neuronal dedifferentiation. Notch (N) lies upstream of Dpn (San‐Juán & Baonza, 2011) and is a Nerfin‐1 target gene. N activation phenocopied loss of Nerfin‐1, resulting in neuron to NB dedifferentiation (Vissers *et al*, 2018; Fig 8A–E). Furthermore, it was recently shown that the N pathway regulates Slp1 and Slp2 expression in medulla NBs through direct binding to Slp1/2 enhancers (preprint: Ray & Li, 2021). To test whether N activation, such as Dpn overexpression, stalled tTF progression, we assessed Slp expression in medulla regions where *UAS‐N* ^*ACT*^ was expressed under the control of *eyR16F10‐GAL4*. We found that the ectopic NBs induced via N^ACT^ exhibited significantly increased expression of the mid‐tTF Slp (Fig 8C–C‴ and F), and resulted in an increase in the number of Toy^+^ neurons (Fig 8I–J′). Similar to Dpn overexpression, dedifferentiated NBs caused by N activation failed to undergo timely terminal cell cycle exit. At 24 h APF, when all wild‐type NBs have exited the cell cycle, NBs generated via N^ACT^ continued to persist (Appendix Fig S2A and H). Next, we asked whether overexpression of D, known to repress Slp, could re‐initiate temporal progression, restore neuronal progeny composition and complete timely terminal differentiation in Notch‐mediated dedifferentiation. Overexpression of D did not significantly affect the rate of dedifferentiation (Fig 8E), but significantly increased the proportion of NBs that expressed the late‐tTF Tll and reduced the proportion of NBs that expressed the mid‐tTF Slp (Fig 8F and G). Consequently, this also resulted in an increase in the amount of Tll^+^ neurons (Fig 8D–D‴ and H) and a reduction in Toy^+^ progeny (Fig 8I–K′).

**Figure 9:**
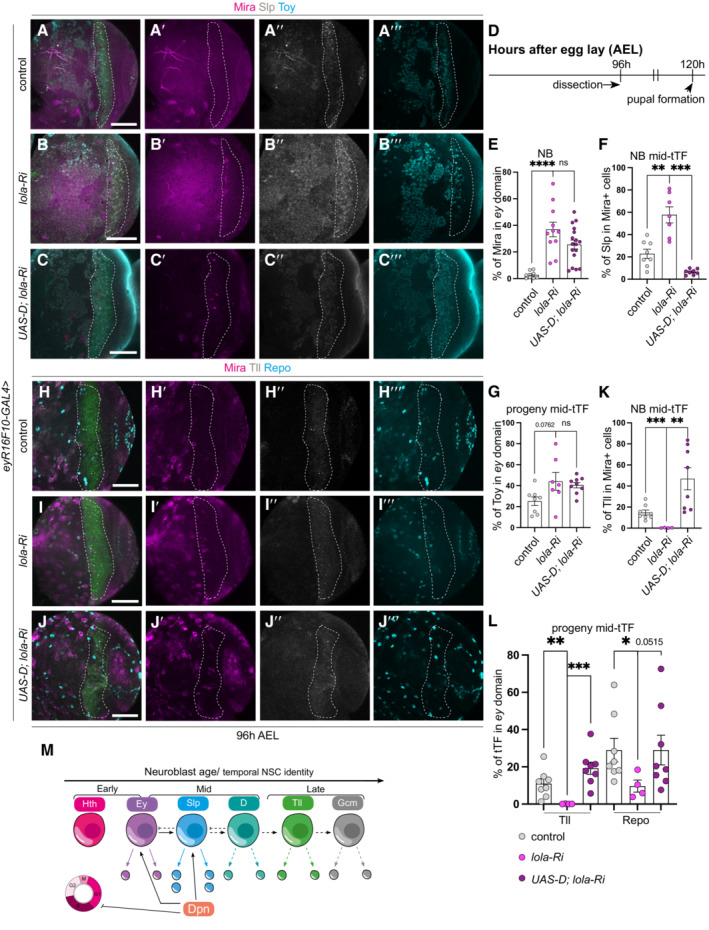
Dichaete re‐initiates the temporal series in dedifferentiated NBs caused by the loss of Lola A–C‴Representative images of the deep medulla neuronal layer in the larval optic lobe, in which control (*UAS‐lacz*), *lola‐Ri*, and *UAS‐D; lola‐Ri* are driven by *eyR16F10‐GAL4* (outlined). Miranda (Mira, magenta), Sloppy‐paired (Slp, grey), Twin of eyeless (Toy, cyan). *lola‐Ri* induces ectopic Mira^+^neuroblasts (NBs) in the deep medulla layers, compared to control, quantified in (E). Dedifferentiated *lola‐Ri* NBs express excess Slp, fewer Tll, and slightly more Toy^+^ neurons compared to control. *UAS‐D; lola‐Ri* NBs express less Slp and similar amount of Toy in *eyR16F10‐GAL4* domain compared to *lola‐Ri* alone, quantified in (F, G).DTimeline depicting the age of ectopic neuroblasts (NBs). Larvae are dissected at 96 h AEL.EQuantification of % Mira‐positive cells within *eyR16F10‐GAL4* expression domain in control, *lola‐Ri*, and *UAS‐D; lola‐Ri* (calculated as the ratio of Mira^+^ cell volume as a percentage of total volume of *eyR16F10‐GAL4* domain). Control *n* = 8, m = 2.717, ±0.9933, *lola‐Ri n* = 11, m = 36.95, ±5.447, *UAS‐D*; *lola‐Ri n* = 17, m = 25.57, ±3.303.FQuantification of % Slp^+^ cells in Mira‐positive cells within the *eyR16F10‐GAL4* expression domain in control, *lola‐Ri*, and *UAS‐D; lola‐Ri* (calculated as % of Slp^+^ cellular volume that are also Mira^+^ within *eyR16F10‐GAL4* domain). Control *n* = 8, m = 22.80 ± 4.065, *lola‐Ri n* = 7, m = 57.68 ± 7.234, *UAS‐D*; *lola‐Ri n* = 8, m = 6.482 ± 0.8840.GQuantification of % Toy^+^ cells within *eyR16F10‐GAL4* expression domain in control, *lola‐Ri*, and *UAS‐D; lola‐Ri* (calculated as the ratio of Toy^+^ cellular volume as a percentage of total volume of *eyR16F10‐GAL4* domain). Control *n* = 8, m = 25.14 ± 4.109, *lola‐Ri n* = 7, m = 44.16 ± 8.518, *UAS‐D*; *lola‐Ri n* = 9, m = 40.53 ± 2.633.H–J‴Representative images of the deep medulla neuronal layer in the larval optic lobe, in which control (*UAS‐lacz*), *lola‐Ri*, and *UAS‐D; lola‐Ri* are driven by *eyR16F10‐GAL4* (outlined). Miranda (Mira, magenta), Tailless (Tll, grey), Reversed polarity (Repo, cyan). *lola‐Ri* NBs express less Tll compared with control. There are fewer Tll^+^ and Repo^+^ progeny in the *eyR16F10‐GAL4* domain in *lola‐Ri* compared to control. *UAS‐D; lola‐Ri* NBs express more Tll as well as more Tll^+^ and Repo^+^ progeny in *eyR16F10‐GAL4* domain compared to *lola‐Ri* alone, quantified in (K, L).KQuantification of % Tll^+^ cells in Mira‐positive cells within the *eyR16F10‐GAL4* expression domain in control, *lola‐Ri*, and *UAS‐D; lola‐Ri* (calculated as % of Tll^+^ cellular volume that are also Mira^+^ within *eyR16F10‐GAL4* domain). Control *n* = 8, m = 14.82 ± 2.400, *lola‐Ri n* = 4, m = 0.2982 ± 0.1217, *UAS‐D*; *lola‐Ri n* = 8, m = 46.85 ± 10.46.LQuantification of % Tll^+^ and Repo cells within *eyR16F10‐GAL4* expression domain in control, *lola‐Ri*, and *UAS‐D; lola‐Ri* (calculated as the ratio of Tll^+^ and Repo^+^ cellular volume as a percentage of total volume of *eyR16F10‐GAL4* domain). Tll (Control *n* = 8, m = 10.94 ± 2.629, *lola‐Ri n* = 4, m = 0.1024 ± 0.05082, *UAS‐D*; *lola‐Ri n* = 8, m = 19.26 ± 3.264), Repo (Control *n* = 8, m = 28.95 ± 6.330, *lola‐Ri n* = 4, m = 9.648 ± 3.228, *UAS‐D*; *lola‐Ri n* = 8, m = 29.00 ± 7.984).MUpon Dpn overexpression, ectopic NBs were induced. These ectopic NBs expressed the mid‐temporal tTF, Slp, and did not express early tTFs or late tTFs. Consequently, an excess of progeny was produced from the Slp^+^ temporal window at the expense of the progeny produced from late tTF time windows. DamID analysis demonstrated that Dpn directly bind to Ey and Slp, but not to Hth or Tll. Therefore, Dpn may be directly responsible for the increased expression of mid‐temporal identity TFs in dedifferentiated NBs at the expense of early or late temporal TFs. Data information: Data are represented as mean ± SEM. *P*‐values were obtained performing unpaired *t*‐test, and Welch's correction was applied in case of unequal variances. ****P < 0.0001, ***P < 0.001, **P < 0.005, *P < 0.05. Scale bars: 50 μm.

We next asked whether dedifferentiation mediated by the downregulation of Lola also stalled the temporal series. As previously reported, the expression of *lola‐*RNAi caused neuron to NB dedifferentiation in the medulla (Fig 9A–E, Southall *et al*, 2014; Zhu *et al*, 2022). Furthermore, the dedifferentiated NBs expressed the mid‐temporal series marker Slp (Fig 9A–F), which produced an excess of Toy^+^ neurons (Fig 9A–C‴ and G), at the expense of Tll^+^ neurons and Repo^+^ glial cells (Fig 9H–J‴, K and L). Overexpression of D, known to repress Slp, was able to promote the re‐initiation of the temporal series, by reducing Slp expressing (Fig 9F), triggering the onset of Tll in the dedifferentiated NBs (Fig 9K), and the production of Tll^+^ neurons and Repo^+^ glial cells (Fig 9L), however, not Toy^+^ neurons (Fig 9G). Together, our data suggest that D overexpression can promote temporal progression stalled via Dpn overexpression, Notch activation or Lola knockdown.

**Figure EV5:**
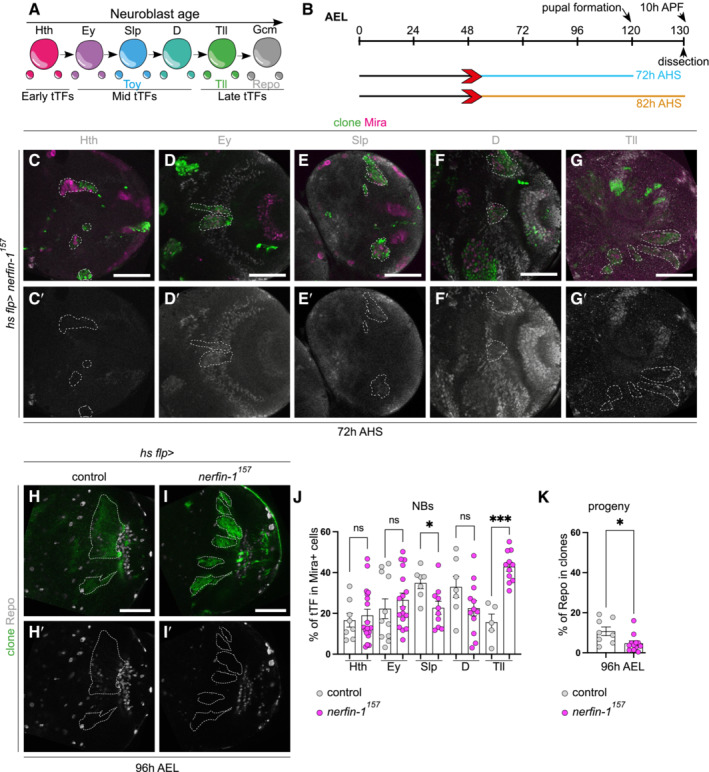
Ectopic NBs generated via *nerfin‐1* express excess Tll at the expense of Repo ASchematic representation of temporal series in neuroblasts (NBs). NBs express temporal transcription factors (tTFs) (Hth, Ey, Slp, D, Tll) as they age. These can be categorised into early, mid, and late tTFs.BSchematic depicting the heat shock regimes used in (C–G′, H–K′). Clones were induced via heat shock (red arrows) and dissected at 72 h (96 h AEL, blue) or 82 h (10 h APF, orange) after heat shock.C–I′(C–G′, H–I′) Representative images of the deep medulla neuronal layer in the larval and pupal optic lobe, in which *nerfin‐1* ^*157*^ is driven in clones by *hs flp* (marked by GFP and outlined) and stained with the stem cell marker, Miranda (Mira, magenta), and various temporal transcription factors (tTFs) (grey). (C–G′) At 72 h AHS, in deep sections of the clone induced via *nerfin‐1* ^*157*^, Mira^+^ NBs express all temporal series transcription factors. Quantified in (J). (H–I′) At 72 and 82 h AHS, there is less Repo^+^ (grey) progeny in *nerfin‐1* ^*157*^ mutant clones compared with the control. Quantified in (K).JQuantification of volume of cells that express a specific tTF as % of total Mira^+^ NB volume within a clone. Hth (Control, *n* = 8, m = 16.63 ± 3.335, *nerfin‐1* ^*157*^, *n* = 18, m = 18.96 ± 3.083), Ey (Control, *n* = 11, m = 22.28 ± 4.833, *nerfin‐1* ^*157*^, *n* = 17, m = 26.59 ± 3.32), Slp (Control, *n* = 7, m = 34.87 ± 2.753, *nerfin‐1* ^*157*^, *n* = 10, m = 22.71 ± 3.18), D (Control, *n* = 7, m = 32.97 ± 5.22, *nerfin‐1* ^*157*^, *n* = 12, m = 22.55 ± 3.768), Tll (Control, *n* = 5, m = 15.65 ± 4.036, *nerfin‐1* ^*157*^, *n* = 11, m = 42.87, ±2.25).KQuantification of % Repo^+^ cells within control and *nerfin‐1* ^*157*^ mutant clones (calculated as the ratio of Repo^+^ cell volume as a percentage of total clone volume). 72 h AHS (Control *n* = 8, m = 10.73 ± 2.148, *nerfin‐1* ^*157*^, *n* = 12, m = 4.741 ± 1.224), 82 h AHS (Control *n* = 6, m = 28.64 ± 6.967, *nerfin‐1* ^*157*^, *n* = 6, m = 8.246 ± 1.301). Data information: Data are represented as mean ± SEM. *P*‐values were obtained performing unpaired *t*‐test, and Welch's correction was applied in case of unequal variances. *P < 0.05 and ***P < 0.001. Scale bars: 50 μm.

Finally, we investigated whether *nerfin‐1* loss‐of‐function clones also exhibited stalled temporal identity. In contrast to Dpn, N overexpression and Lola knockdown, *nerfin‐1* mutant clones exhibited normal progression through the Hth → Ey → Slp → D temporal transcription factor widows (Fig EV5A–G′ and J). However, as previously reported (Zhu *et al*, 2022), the loss of *nerfin‐1* caused stalling of the temporal series at Tll (Fig EV5A–G′ and J), preventing the Tll^+^ neurogenesis to gliogenesis transition, as indicated by a deficit of Repo^+^ glial cells at 96‐h AEL (Fig EV5H–I′ and K).

## Discussion

In this study, our data argue that upon NB dedifferentiation in the OL, the proliferative potential of NBs and their ability to produce the correct neuronal subtypes is determined by cell cycle progression and correct expression of the temporal transcription factors (tTFs). When ectopic NBs are induced by Dpn overexpression, they adopt a mid‐temporal identity, leading to significantly increased numbers of mid‐temporal identity progeny. They also have delays in cell cycle progression and terminal cell cycle exit. Retriggering the temporal series via D overexpression or promoting the cell cycle was sufficient to reinstate neuronal diversity and timely NB termination.

Although ectopic NBs formed through Dpn overexpression were locked into a mid‐temporal fate, we found that temporal transitions in dedifferentiated NBs obey the feed forward activation and feedback repression that occurs in wild‐type medulla NBs (Fig EV3C–D‴). The mid‐tTFs sit within a mutually exclusive regulatory network in which only one tTF can be active at any stage (Li *et al*, 2013; Zhu *et al*, 2022), and consistent with this, we observed D to be expressed in a complementary pattern to that of Slp in ectopic NBs. The preference of Dpn binding at the mid‐tTFs in medulla NBs, together with the enrichment of the mid‐tTF fates, suggests a model in which Dpn overexpression activates the mid‐tTF network in ectopic NBs. Consistent with this, we observed enriched binding of Dpn at mid‐tTF loci in wild‐type medulla NBs, and reduced Dpn binding at early‐ and late‐tTF loci. Our data suggest that Dpn promotes the expression of mid‐tTFs such as Ey and Slp (Fig 9M). Although Dpn has been historically classed as a transcriptional repressor, a previous study profiling Dpn binding in larval NB hyperplasia via ChIP‐seq reported that half of the directly regulated targets of Dpn were upregulated in tumours, and half were downregulated, consistent with a dual activator/repressor role for this protein (Magadi *et al*, 2020).

The delay in temporal progression appears to be a general feature of dedifferentiation in the OL, as a delay in dedifferentiation was observed upon Dpn or Notch activation, as well as the downregulation of Lola and Nerfin‐1. The OL is an area of the brain where the differentiated state of the neurons appears to be less stable (Xu *et al*, 2017). However, we do not yet know whether dedifferentiation‐mediated tumours arising from other regions of the CNS would also exhibit delayed temporal progression, as different sets of temporal factors are used in the ventral nerve cord and Type II lineages (Maurange *et al*, 2008; Ren *et al*, 2017). It would be interesting to explore whether N or tumour suppressors such as Brain Tumour (Brat) similarly regulate the mid‐tTF genes, as it has previously been shown that Brat regulates both Dpn and Notch (Reichardt *et al*, 2018). However, it could be that N acts partially through Dpn, as Dpn has been shown to be a target of N in NBs (San‐Juán *et al*, 2012).

Our data support the possible links between cell cycle progression and the expression of temporal regulators controlling NB proliferation and cellular diversity. In the embryonic NBs, temporal transition can progress despite of experimentally induced cell cycle arrest (Grosskortenhaus *et al*, 2005). Whereas in Type I larval NBs, it was shown that G1/S‐cell cycle delay resulted in stalled temporal patterning and reduced neuronal diversity (van den Ameele & Brand, 2019). In dedifferentiated OL NBs, it appears that the temporal series also lies downstream of the cell cycle. However, in this context, we show that the cell cycle can direct and regulate the progression of the temporal series and is responsible for restoring neuronal diversity caused by temporal series delay. However, re‐initiation of the temporal series was not sufficient to rescue cycle cycle progression.

Perturbation of different neuronal factors can impact different points of the neurogenesis temporal series. It is so far unclear why *nerfin‐1* mutant clones differ from the other dedifferentiation factors, where they are stalled at the Tll^+^ window rather than Slp^+^ window. Future studies using ATAC‐seq to examine the chromatin accessibility of the temporal transcription factors for each of these dedifferentiation factors may shed light on the specificity. However, our work together with previous work using another dedifferentiation tumour model (*pros* loss of function) shows that specification of the correct temporal cascade is instrumental to tumour malignancy and is a key mechanism underlying tumourigenesis (Narbonne‐Reveau *et al*, 2016; Genovese *et al*, 2019; Maurange, 2020; Gaultier *et al*, 2022).

Recent studies suggest that a bidirectional conversion between tumour‐initiating stem cells and differentiated cells exist, where non‐cancer stem cells can dedifferentiate and acquire stem cell‐like properties under genetic and environmental stress (Friedmann‐Morvinski & Verma, 2014). Dedifferentiation of mature cells to induce their proliferation and re‐differentiation has also been shown to be a promising means of tissue repair and regeneration. Therefore, dedifferentiation may have either positive or negative consequences for tissue repair and tumorigenesis. Our work shows that appropriate temporal and cell cycle progression controls are key to regulating the fine balance between tumorigenesis and controlled dedifferentiation.

## Materials and Methods

### Fly husbandry

Fly stocks were reared on standard *Drosophila* media at 25°C. For larval dissection, brains were dissected at wandering L3 stages. For pupal dissection, white pupae were selected and then allowed to age at 29°C, dissections were made at 6, 10, 16 or 24 h after pupal formation (APF).

GAL4 lines *GMR31H08‐GAL4*, *ey* ^*OK107*^ *‐GAL4* and *eyR16F10‐GAL4* were used to induce dedifferentation. *GMR31H08‐Gal4* (Jenett *et al*, 2012) is a neuronal‐specific driver (Fig EV1E–F‴), this line together with a GAL80‐ts was used to demonstrate that mature neurons can undergo dedifferentiation (Fig EV1A–D). *ey* ^*OK107*^ *‐GAL4* (Morante *et al*, 2011) is expressed in both neurons and NBs during larval neurogenesis (Fig EV1I–J‴), *and eyR16F10‐GAL4* is expressed in both neurons and NBs (Fig EV1O–P‴). *eyR16F10‐GAL4* but not *ey* ^*OK107*^ *‐GAL4* was strongly expressed in the medulla neurons during pupal development (Fig EV1Q and R, and K and L); therefore, *eyR16F10‐GAL4* was utilised in all the pupal analysis. *ey* ^*OK107*^ *‐GAL4* was used to make compound stocks with *Toy‐GFP* and was restricted to larval analysis (Morante *et al*, 2011; Jenett *et al*, 2012). Knockdown or overexpression CNS clones were generated using the flpout system. *hsFLP; actc5 > CD2 > Gal4*, *UAS‐GFP/TM6b* (Vissers *et al*, 2018). Flpout clones were induced by heat shock at 16, 24, 48 h, 72 or 96 h after egg lay (AEL) for 6 min, shifted to 29°C and dissected at 120 h AEL. The fly strains used were as follows: *UAS‐deadpan* (A. Baonza), (#1776, Bloomington), *UAS‐Nerfin‐1 RNAi* (VDRC), *UAS‐mCherry RNAi* (#35785, Bloomington), *Toy‐GFP* (#83390, Bloomington), *UAS‐N* ^*ACT*^ (#52309, Bloomington), *UAS‐Cdk4; UAS‐CycD* (Helena Richardson), *UAS‐E2f1; UAS‐CycE* (Helena Richardson), *UAS‐D* (#8861, Bloomington), *UAS‐lola* RNAi (#35721, Bloomington). *Nerfin‐1* ^*157*^ mutant clones (Froldi *et al*, 2015) were generated using the MARCM system (w, tub‐Gal4, UAS‐nlsGFP::6xmyc::NLS, hs‐flp; FRT2A, tubP‐Gal80LL9/TM6B).

### Immunostaining

Larval and pupal brains were dissected out in phosphate buffered saline (PBS), fixed for 20 min in 4% formaldehyde in PBS and rinsed in 0.2% PBST (PBS + 0.2% TritonX‐100). For immunostaining, brains were incubated with primary antibodies overnight at 4°C, followed by an overnight secondary antibody incubation at 4°C. Samples were mounted in 80% glycerol in PBS for image acquisition. The primary antibodies used were mouse anti‐Mira (1:50; gift of Alex Gould), rat anti‐Mira (1:100, Abcam), rat anti‐pH3 (1:500; Abcam), chick anti‐GFP (1:2000; Abcam), rabbit anti‐RFP (1:100, Abcam), mouse anti‐Repo (1:50, DSHB), guineapig anti‐Hth (1:100, Claude Desplan), mouse anti‐Ey (1:50, DSHB), guineapig anti‐Slp (1:500, Kuniaki Saito), rabbit anti‐D (1:1,000, Steve Russell), guineapig anti‐Tll (1:100, Kuniaki Saito), rabbit anti‐Tll (1:100, Kuniaki Saito) and guineapig anti‐Toy (1:50, Uwe Walldorf). Secondary donkey antibodies conjugated to Alexa 555 and Alexa 647, and goat antibody conjugated to Alexa 405, 488, 555 and 647 (Molecular Probes) were used at 1:500.

### EdU pulse/chas

Control and *UAS‐d*pn clones were induced 48 h AEL. 48 h after clone induction, larvae were fed with 100 mg/ml EdU for 3 h. They were then transferred to standard medium for a 3h EdU‐free chase. Larvae were dissected, fixed and processed for antibody staining, followed by EdU detection with Click‐iT Plus EdU Cell Proliferation Kit for imaging, Alexa Fluor 647 dye (Invitrogen, #C10640) according to the manufacturer's instruction.

### Imaging and image processing

Images were collected on a Leica SP5/Olympus FV3000 confocal microscope and processed using Fiji (https://imagej.net/Fiji). Z stacks of optic lobes were imaged, and single sections were shown as the representative image in the figures. All images represent the medulla deep section unless specifically stated.

GFP^+^, Mira^+^ NBs, Toy^+^ neurons, *ey* ^*OK107*^ *‐GAL4* expressing domain and TF volume were measured using flurescence intensity from three‐dimensional reconstructions of 2‐μm spaced confocal Z‐stacks with Volocity software (Improvision) or Imaris (Bitplane). The rate of dedifferentiation was represented as the volume of Mira^+^ cells as a percentage of clone volume. The percentage of TFs was calculated as the volume of TF^+^ cells as a percentage of clone volume or domain volume. The percentage of Toy^+^ neurons was calculated as the volume of Toy^+^ neurons as a percentage of *ey* ^*OK107*^ *‐GAL4* expressing domain volume. *eyR16F10‐GAL4* domain was not labelled with a fluorescent marker, a Region of Interest (ROI) was manually selected in each of the slice across the Z sections, as guided by the outline of the overlaying superficial NBs.

### Statistical analysis

At least three animals per genotype were used for all experiments. Volume of clones or regions of interest was estimated from three‐dimensional reconstructions of 2‐mm spaced confocal Z stacks with the Volocity software (Improvision).

In all graphs and histograms, error bars represent the standard error of the mean (SEM) and *P*‐values are calculated by performing two‐tailed, unpaired Student's *t*‐test. The Welch's correction was applied in case of unequal variances.

### Targeted DamID

FlyORF‐TaDa‐Dpn flies were created from the FlyORF‐Dpn‐3xHA line (FlyORF stock# F000086) with the FlyORF‐TaDa system as previously described (Aughey *et al*, 2021). Targeted DamID for Dpn in 3rd instar larval neuroblasts was performed as previously described (Marshall & Brand, 2015; Marshall *et al*, 2016), crossing FlyORF‐TaDa‐Dpn flies with the neuroblast‐specific wor‐Gal4;tub‐GAL80ts driver. NanoDam (Tang *et al*, 2022) for Dpn in 3^rd^ instar larval medulla NBs was performed using *TaDaM‐traNLS‐vhhGFP* (preprint: Delandre *et al*, 2022) crossing *TaDaM‐traNLS‐vhhGFP*, *tub‐GAL80ts; eyR16F10‐GAL4* to either *Dpn‐GFP* (BL# 65295) or the *w* ^*1118*^ genetic background. In both cases, flies were allowed to lay on apple juice plates with yeast for 4 h at 25°C, before transferring plates to 18°C for 2 days. 100 larvae from each plate were transferred to food plates and grown at 18°C for a further 5 days, before shifting to the permissive temperature of 29°C. Two biological replicates were collected, of 30 dissected brains per replicate (Dpn TaDa) or 40 brains per replicate (Dpn NanoDam).

Samples were processed for DamID as previously described (Marshall *et al*, 2016). Briefly, genomic DNA was extracted, cut with DpnI and cut fragments isolated. DamID adaptors were ligated to the isolated DNA, fragments were digested with DpnII, and amplified via PCR. Following PCR, samples were sonicated in a Bioruptor Plus (Diagenode) to reduce the average DNA fragment size to 300 bp, and DamID adaptors were removed via overnight AlwI digestion. The resulting DNA was purified via magnetic bead cleanup and 500 ng of DNA was end‐repaired, A‐tailed, ligated to NGS adaptors and amplified via six PCR cycles. The final libraries, multiplexed to yield ~20 million mappable fragments per sample, were sequenced as paired‐end reads (MGI platform, BGI).

NGS reads in FASTQ format were aligned using damidseq_pipeline (Marshall & Brand, 2015) using default options, and replicates were averaged to give the final binding profile. Significantly enriched peaks were called for each replicate using find_peaks (Marshall *et al*, 2016) with parameters ‐‐min‐quant = 0.8 and ‐‐unified_peaks = min with otherwise default options; peaks were considered significant at FDR < 0.01. Peaks for all replicates and conditions were combined using the GenomicRanges R package, the per‐replicate binding occupancy across each region determined, and diferentially bound peaks were called using NOISeq (Tarazona *et al*, 2015) at *q* = 0.85. Peaks were associated with genes within ±1 kb of a peak. Datasets were visualised using pyGenomeTracks (Cubeñas‐Potts *et al*, 2017).

## Author contributions

**Kellie Veen:** Conceptualization; data curation; formal analysis; investigation; methodology; writing – original draft; writing – review and editing. **Phuong‐Khanh Nguyen:** Data curation; formal analysis; investigation; methodology. **Francesca Froldi:** Data curation; formal analysis; supervision; investigation; methodology; writing – original draft; writing – review and editing. **Qian Dong:** Data curation; formal analysis; investigation. **Edel Alvarez‐Ochoa:** Data curation; formal analysis; methodology. **Kieran F Harvey:** Writing – review and editing. **John PD McMullen:** Data curation; formal analysis; investigation; methodology. **Owen Marshall:** Conceptualization; formal analysis; investigation; methodology; writing – original draft; project administration; writing – review and editing. **Patricia R Jusuf:** Supervision. **Louise Y Cheng:** Conceptualization; data curation; supervision; funding acquisition; writing – original draft; project administration; writing – review and editing.

## Disclosure and competing interests statement

The authors declare that they have no conflict of interest.

## Supporting information



Appendix

Expanded View Figures PDF

PDF+

## Data Availability

NGS data have been deposited in the NCBI Gene Expression Omnibus (GEO) and are available under accession number GSE225409.
