# Supplementary material for: Dedifferentiation‐derived neural stem cells exhibit perturbed temporal progression
Source: EMBO Rep. 2023 Apr 11;24(6):e55837. doi: 10.15252/embr.202255837 (PMC10240205; doi:10.15252/embr.202255837)
Supplement: Supplementary file 1 — Appendix [file EMBR-24-e55837-s002.pdf]

Appendix File Table of Contents

Appendix figure S1 Dpn overexpression induces proliferative NBs and Dpn is epistatic to Nerfin-1 RNAi 2

Figure 2

Figure legend 3

Appendix figure S2 N^{ACT} ectopic NBs do not terminate at 24 hr APF and Cdk4+CycD promote temporal progression in dedifferentiated NBs induced via Dpn overexpression 4

Figure 4

Figure legend 5

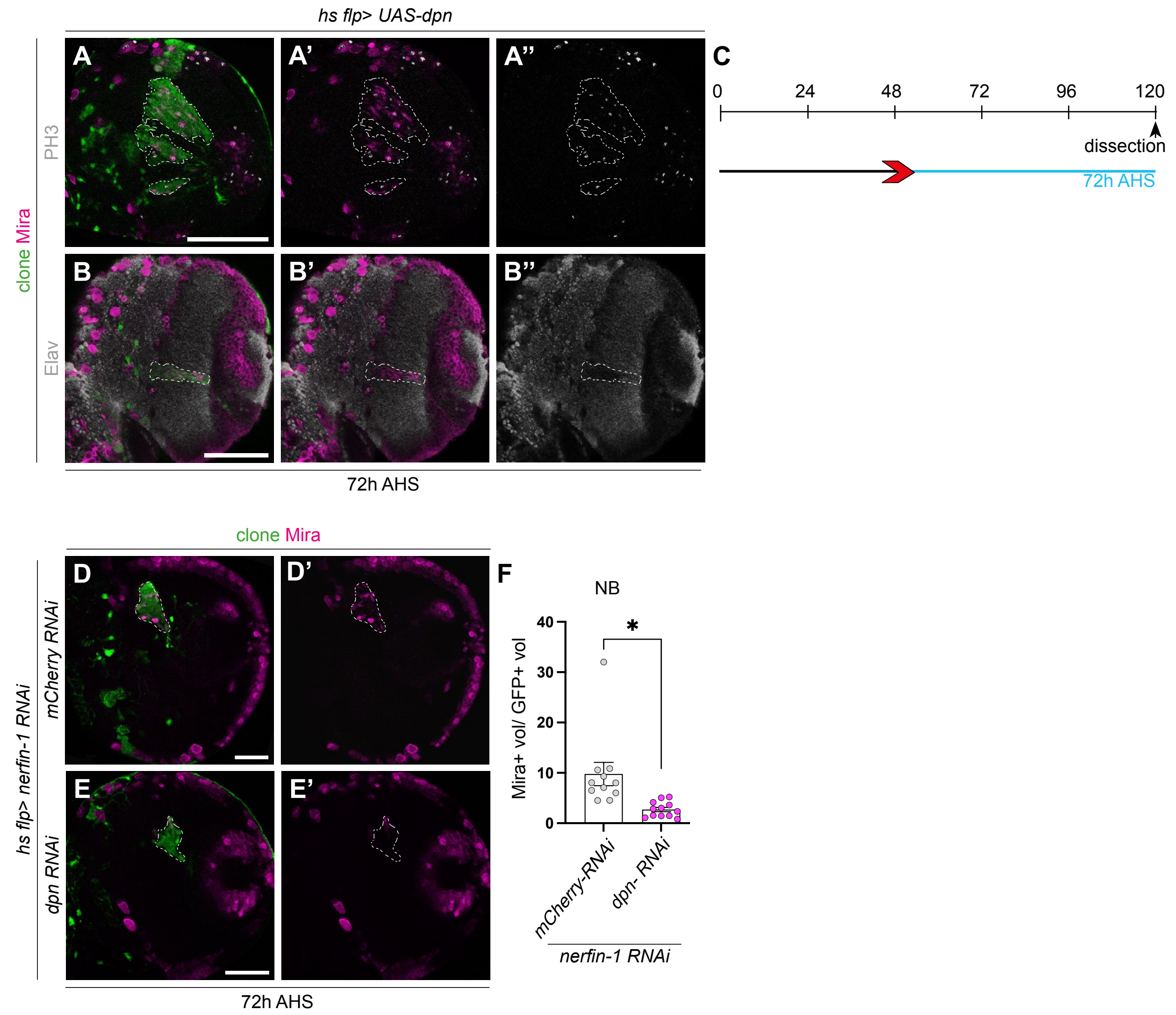
hs flp> UAS-dpn
A
A'
A''
B
B'
B''
clone Mira
PH3
Elav
72h AHS
C
0
24
48
72
96
120
dissection
72h AHS
clone Mira
D
D'
E
E'
hs flp> nerfin-1 RNAi
mCherry RNAi
dpn RNAi
72h AHS
F
NB
*
Mira+ vol/ GFP+ vol
40
30
20
10
0
mCherry-RNAi
dpn- RNAi
nerfin-1 RNAi

Appendix figure S1 Dpn overexpression induces proliferative NBs and Dpn is epistatic to Nerfin-1 RNAi

(A-B'') Representative images of the deep medulla neuronal layer in the larval optic lobe, in which *UAS-dpn* and control clones are generated via *hs flp*. At 72h after clone induction, Mira+ NBs (magenta) express M phase marker PH3 (grey) and do not express neuronal marker Elav (grey).

(C) Heat shock regime for (A-B' and E-F''). Clones were induced by heat shock (red) at 48 hr AEL and dissected 72 hr (blue) after heat shock.

(D-E') Representative images of the deep medulla neuronal layer in the larval optic lobe, in which *UAS-dpn*, *nerfin-1 RNAi*; *mCherry RNAi*, *nerfin-1 RNAi; dpn RNAi* are driven in clones by *hs flp* (marked by GFP and outlined) and stained with the stem cell marker, Miranda (Mira, magenta). Less Mira+ ectopic NBs are induced when driven by *nerfin-1 RNAi; dpn RNAi*, compared to *nerfin-1-Ri* alone, quantified in F.

(F) Quantification of % Mira+ cells in control *nerfin-1 RNAi; mCherry RNAi* and *nerfin-1 RNAi; dpn RNAi* clones (calculated as the ratio of Mira+ cell volume as a percentage of total clone volume). *nerfin-1 RNAi; mCherry RNAi* n=11, m=9.774 ±2.325, *nerfin-1 RNAi; dpn RNAi* n=12, m=2.722 ±0.441.

Data are represented as mean ± SEM. P-values were obtained performing unpaired t-test, and Welch's correction was applied in case of unequal variances. *$p < 0.05$.
Scale bars: 50 µm.

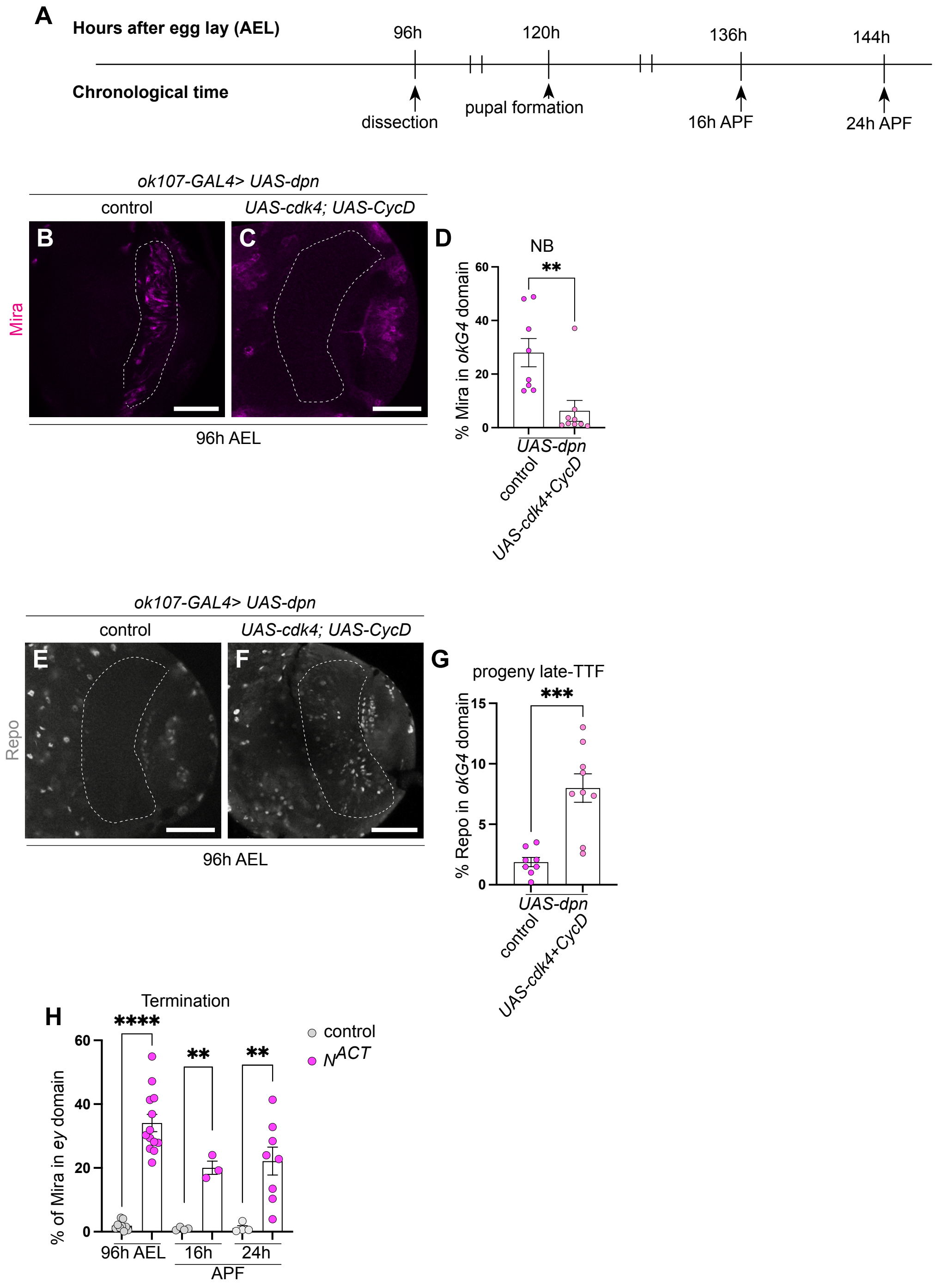
A
Hours after egg lay (AEL)
96h
120h
136h
144h
Chronological time
dissection
pupal formation
16h APF
24h APF
ok107-GAL4> UAS-dpn
control
UAS-cdk4; UAS-CycD
B
C
Mira
96h AEL
D
NB
**
% Mira in okG4 domain
60
40
20
0
UAS-dpn
control
UAS-cdk4+CycD
ok107-GAL4> UAS-dpn
control
UAS-cdk4; UAS-CycD
E
F
Repo
96h AEL
G
progeny late-TTF
***
% Repo in okG4 domain
15
10
5
0
UAS-dpn
control
UAS-cdk4+CycD
H
Termination
****
**
**
control
N^ACT
% of Mira in ey domain
60
40
20
0
96h AEL
16h
24h
APF

Appendix figure S2 N^{ACT} ectopic NBs do not terminate at 24 hr APF and Cdk4+CycD promote temporal progression in dedifferentiated NBs induced via Dpn overexpression

(A) Time line depicting age of ectopic neuroblasts (NBs). Larvae are dissected at 96 hr. Pupal formation occurs at 120 hr AEL; pupae are dissected at 136 hr (16 hr APF) and 144 hr AEL (24 hr APF).

(B-C) Representative images of the deep medulla neuronal layer in the larval optic lobe, where *UAS-cdk4; UAS-CycD*; *UAS-dpn* or *UAS-dpn; UAS luc* are expressed in the *ok107-GAL4* domain (outlined), and stained with the stem cell marker, Mira (magenta). Quantified in D.

(D) Quantification of % Mira-positive cells within *ok107-GAL4* expression domain in *UAS-dpn* control and *UAS-cdk4; UAS-CycD*; *UAS-dpn* (calculated as the ratio of Mira+ cell volume as a percentage of total *ok107-GAL4* domain volume). *UAS-dpn* control n= 8, m= 27.99, ±5.288, *UAS-cdk4; UAS-CycD*; *UAS-dpn*, n= 9, m= 6.275, ±3.906.

(E-F) Representative images of the deep medulla neuronal layer in the larval optic lobe, where *UAS-dpn* control or *UAS-cdk4; UAS-CycD; UAS-dpn* are expressed in the *ok107-GAL4* domain. Glial cells are labelled with Repo (grey) within the *ok107-GAL4* domain (outlined). Quantified in G.

(G) Quantification of % Repo-positive cells within *ok107-GAL4* expression domain in *UAS-dpn* and *UAS-cdk4; UAS-CycD; UAS-dpn* (calculated as the ratio of Repo+ cell volume as a percentage of total *ok107-GAL4* domain volume). *UAS-dpn* n= 8, m= 1.875, ±0.3837, *UAS-cdk4; UAS-CycD; UAS-dpn* n= 9, m= 7.997, ±1.173.

(H) Quantification of % Mira+ cells within *ok107-GAL4* expression domain in *UAS-*N^{ACT} and control (calculated as the ratio of Mira+ cell volume as a percentage of total *ok107-GAL4* domain volume). 96 hr (Control n= 9, m= 1.874 ±0.4897, *UAS-*N^{ACT} n= 13, m= 34.06, ±2.7). 136 hr (Control n= 4, m= 0.8897, ±0.239, *UAS-*N^{ACT} n= 3, m= 20.04 ±2.091). 144 hr (Control n= 4, m= 1.174 ±0.7226, *UAS-*N^{ACT} n= 8 m= 22.14, ±4.382).

Data are represented as mean ± SEM. P-values were obtained performing unpaired t-test, and Welch correction was applied in case of unequal variances. ****$p<0.0001$, ***$p<0.001$, **$p<0.005$, *$p<0.05$. Scale bars: 50 μm.
