# Supplementary material for: Dedifferentiation‐derived neural stem cells exhibit perturbed temporal progression
Source: EMBO Rep. 2023 Apr 11;24(6):e55837. doi: 10.15252/embr.202255837 (PMC10240205; doi:10.15252/embr.202255837)
Supplement: Supplementary file 2 — Expanded View Figures PDF [file EMBR-24-e55837-s003.pdf]

Expanded View Figures

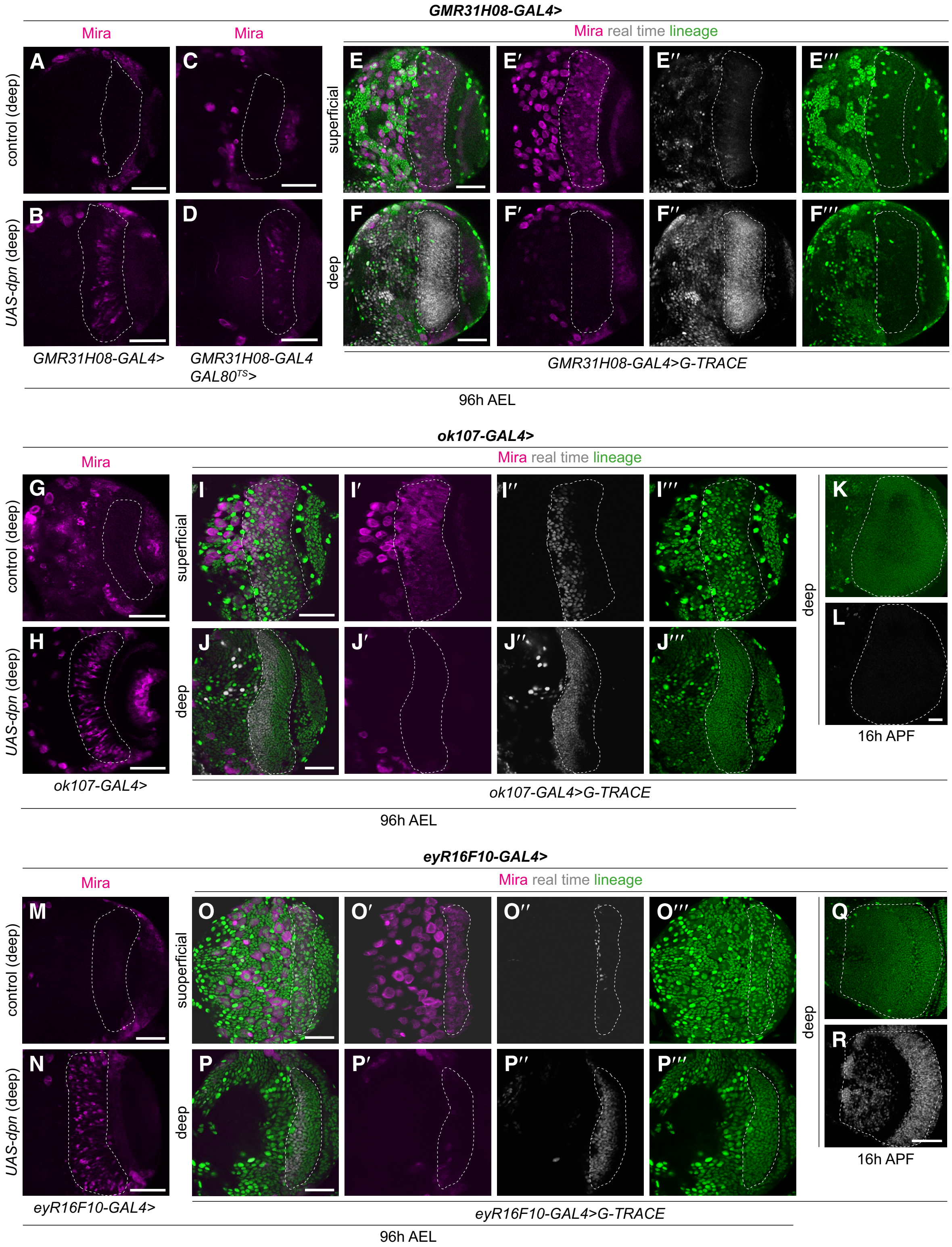

Figure EV1.

 © 2023 The Authors

Figure EV1. Lineage analysis of drivers utilised to induce dedifferentiation.

A–R Representative images of the deep medulla neuronal layer or neuroblast (NB) superficial layer in the larval optic lobe, in which *UAS-dpn* and control are expressed using *GMR31H08-GAL4*, *ok107-GAL4*, and *eyR16F10-GAL4* (outlined) and stained with the neuroblast (NB) marker, Miranda (Mira, magenta). (A–D) *GMR31H08-GAL4* and *GMR31H08-GAL4 GAL80^{TS}* driving *UAS-dpn* induces ectopic Mira^{+} cells in deep medulla layers compared to control. (E–F‴) *GMR31H08-GAL4* is expressed in real time (grey) mainly in medulla neurons within the deep layer compared to NBs in the superficial layer. (G, H) *ok107-GAL4* driving *UAS-dpn* induces ectopic Mira^{+} cells in deep medulla layers compared to control. (I–L) *ok107-GAL4* is expressed in real time (grey) in medulla neurons within the deep layer and NBs in the superficial layer 96 h AEL, however, its expression is not maintained at 16 h APF, therefore, this driver is unsuitable for examination of NB termination. (M, N) *eyR16F10-GAL4* driving *UAS-dpn* induces ectopic Mira^{+} cells in deep medulla layers compared to control. (O–R) *eyR16F10-GAL4* is expressed in real time (grey) in medulla neurons within the deep layer and few NBs in the superficial layer 96 h AEL and is expressed at 16 h APF and is therefore a suitable driver for examination of NB termination. Scale bars: 50 μm.

© 2023 The Authors

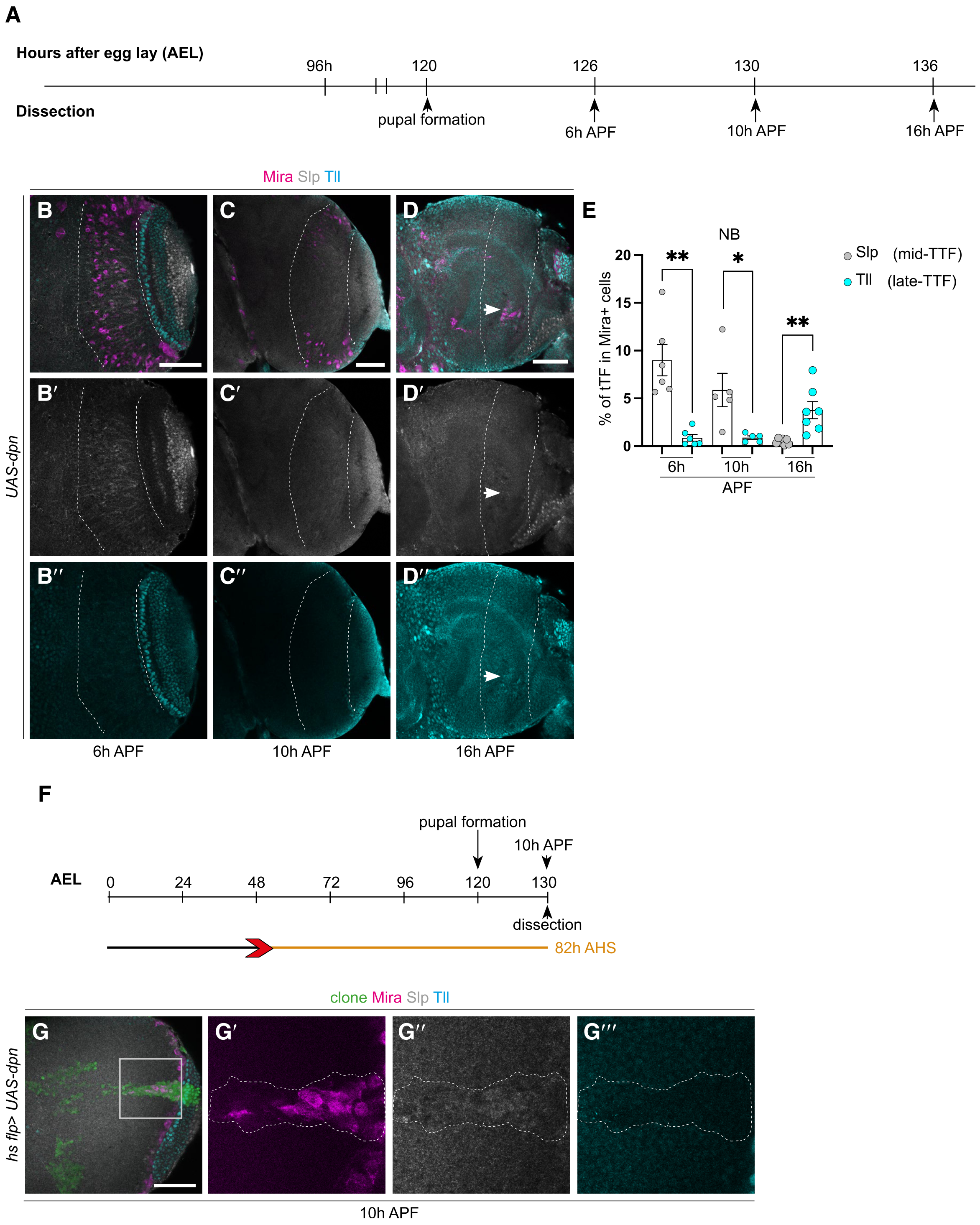

Figure EV2.

© 2023 The Authors

◀ **Figure EV2. Slp and Tll expression during terminal differentiation in ectopic NBs generated via Dpn overexpression.**

A Time-line depicting age of ectopic neuroblasts (NBs). Pupal formation occurs at 120 h AEL; pupae are dissected at 126 h (6 h APF), 130 h (10 h APF), and 136 h (16 h APF).

B–D″ Representative images of the deep medulla neuronal layer in the pupal optic lobe, in which *UAS-dpn* is driven by *eyR16F10-GAL4* (outlined) and stained with the stem cell marker, Miranda (Mira, magenta), mid-tTF, Sloppy-paired (Slp, grey), and late tTF, Tailless (Tll, cyan). Dedifferentiated NBs express Slp and do not express Tll at 6 h APF and 10 h APF; however, they by 16 h APF they do not express Slp and instead express Tll. Quantified in (E).

E Quantification of % Slp^+ or Tll^+ cells in Mira-positive cells within the *eyR16F10-GAL4* expression domain in *UAS-dpn* (calculated as the ratio of Slp/Tll^+ cell volume and $Mira^+$ volume within *eyR16F10-GAL4* domain). Six hours APF (Slp n = 6, m = 8.999 ± 1.64, Tll n = 6, m = 0.8718, ±0.3451). Ten hours APF (Slp n = 5, m = 5.876, ±1.752, Tll n = 5, m = 0.89 ± 0.1915). Sixteen hours APF (Slp n = 7, m = 0.4699, ±0.116, Tll n = 7, m = 3.767 ± 0.8893).

F Heat shock regime for (G–G‴). Clones were induced by heat shock (red) at 48 h AEL and dissected at 126 h AEL (6 h APF), corresponding to 82 h (blue) hours after heat shock.

G′–G‴ Are magnified images of (G). At 82 h after clone induction (10 h APF), $Mira^+$ NBs express Slp and not Tll.

Data information: Data are represented as mean ± SEM. *P*-values were obtained performing unpaired *t*-test, and Welch's correction was applied in case of unequal variances. $^{**}P < 0.005$, $^{*}P < 0.05$. Scale bars: 50 μm.

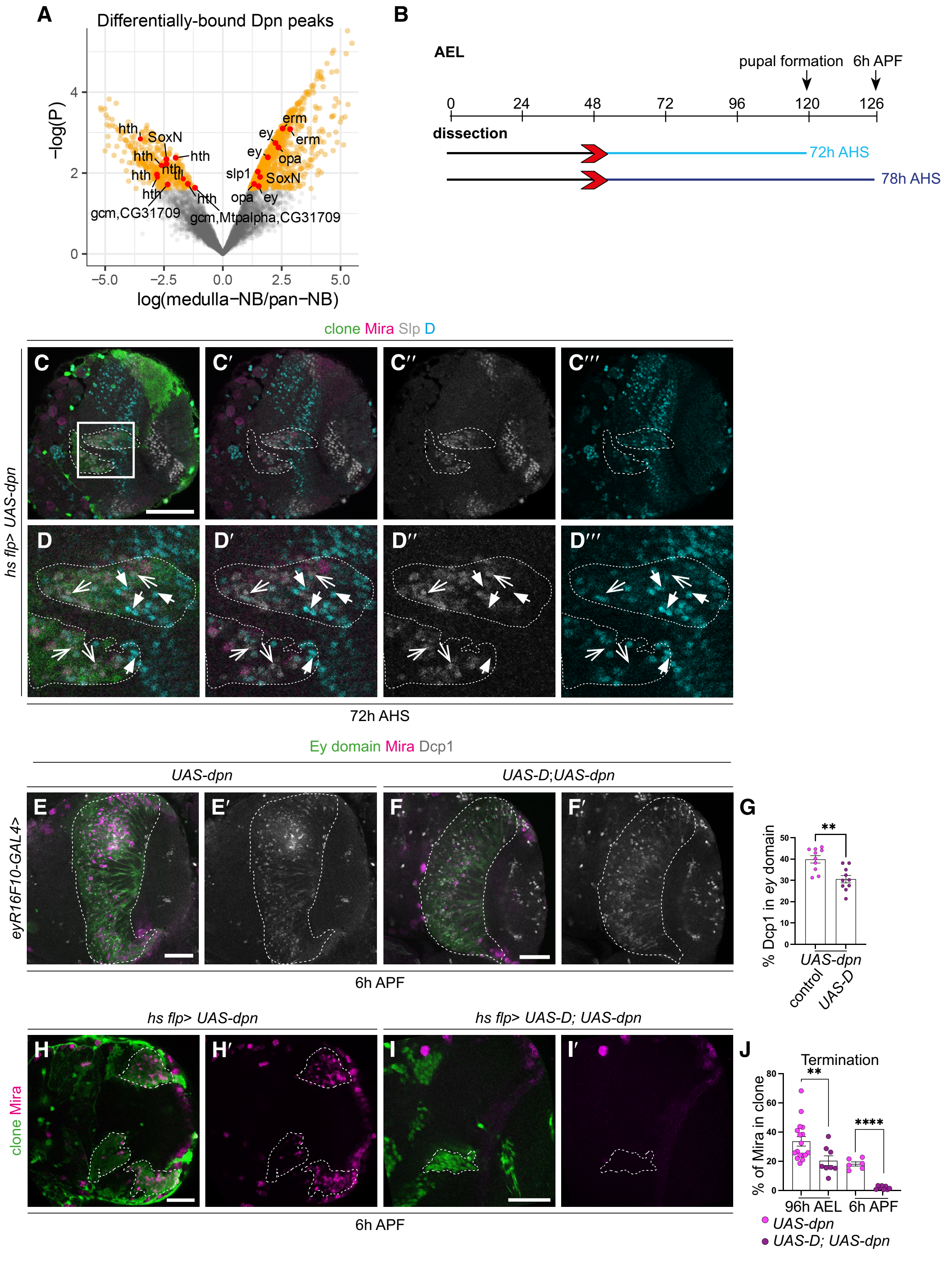

Figure EV3.

 © 2023 The Authors

Figure EV3. Slp and D are expressed in complementary patterns and *UAS-D* promotes premature termination of *UAS-dpn* ectopic NBs.

- A Volcano plot of differentially bound by Dpn in the medulla. The temporal transcription factors (tTFs) depicted here include the more comprehensive network of transcription factors from Zhu *et al* (2022).
- B Schematic depicting the heat shock regimes used in (C–D‴ and H–I′). Clones were induced via heat shock (red arrows) at 48 h and dissected 72 h (light blue) or 78 h (6 h APF, dark blue) after heat shock.
- C–D‴ Representative images of the deep medulla neuronal layer in the larval optic lobe, where *UAS-dpn* generated by *hs flp*, express complementary patterns of Slp and D expression. (D–D‴) are magnified of (C–C‴). At 72 h after clone induction, Mira$^+$neuroblasts (NBs) express Slp or D. Open arrow heads represent NBs that express Slp and not D. Closed arrow heads represent NBs that express D and not Slp. Miranda (Mira, magenta), D (cyan), Slp (grey).
- E–F′ Representative images of the deep medulla neuronal layer in the larval optic lobe, where less cell death as detected by Death caspase 1 (Dcp1, grey) is found in ectopic NBs (Mira (magenta)) induced via *UAS-D*; *UAS-dpn* compared to *UAS-dpn* (driven by *eyR16F10-GAL4*). Quantified in (G).
- G Quantification of % Dcp1$^+$ cells within the *eyR16F10-GAL4* expression domain in the deep section of *UAS-dpn* or *UAS-D*; *UAS-dpn* (calculated as the ratio of Dcp1$^+$ cell volume within *eyR16F10-GAL4* domain). *UAS-dpn* $n = 10$, $m = 39.87 \pm 1.720$, *UAS-D*; *UAS-dpn* $n = 10$, $m = 30.59 \pm 1.715$.
- H–I′ Representative images of the deep medulla neuronal layer in the larval optic lobe, where *UAS-dpn* or *UAS-D; UAS-dpn* clones are induced via *hs flp*. NBs are marked by stem cell marker, Mira (magenta). At 96 h AEL and 6 h APF, NBs underwent premature terminal differentiation upon overexpression of (D), quantified in (J).
- J Quantification of % Mira$^+$ cells in control *UAS-dpn* and *UAS-D; UAS-dpn* clones (calculated as the ratio of Mira$^+$ cell volume as a percentage of total clone volume). 72 h (*UAS-dpn* $n = 17$, $m = 33.62 \pm 3.243$, *UAS-D; UAS-dpn* $n = 8$, $m = 20.38 \pm 3.307$). 78 h (*UAS-dpn* $n = 6$, $m = 18.3 \pm 1.46$, *UAS-D; UAS-dpn* $n = 8$, $m = 1.859 \pm 0.4215$).

Data information: Data are represented as mean ± SEM. *P*-values were obtained performing unpaired *t*-test, and Welch correction was applied in case of unequal variances. ****$P < 0.0001$, **$P < 0.005$. Scale bars: 50 µm.

© 2023 The Authors

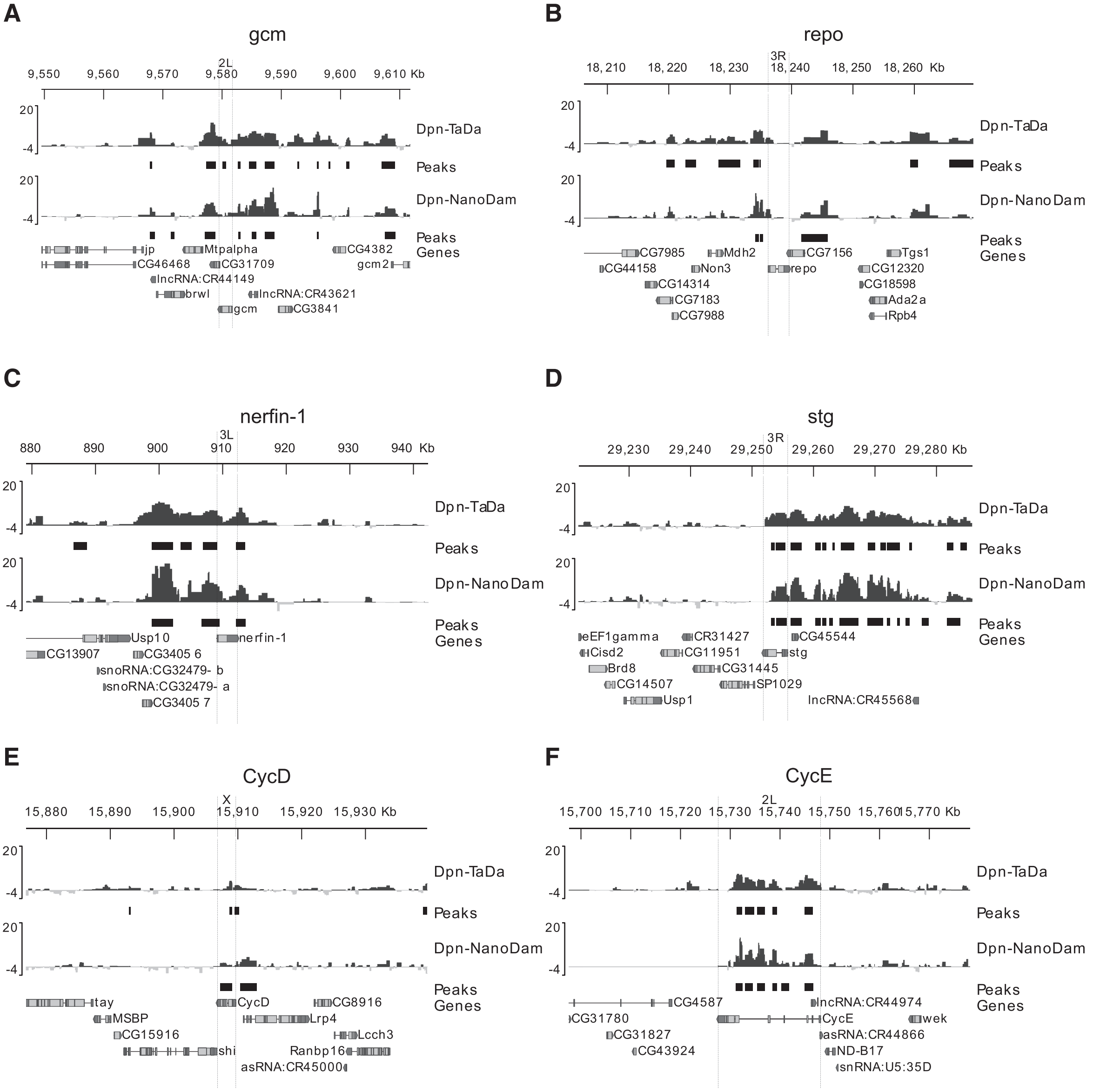

Figure EV4. Dpn binds to tTFs and cell cycle loci.

A–F Mean Targeted DamID and Dpn-NanoDam Dpn binding profiles (log2(Dam-fusion/Dam)) are shown for the optic lobe temporal TFs (A) gcm, (B) repo, (C) nerfin-1; and for the cell cycle genes (D) stg, (E) CycD and (F) CycE. peaks with FDR < 0.01 are shown.

 © 2023 The Authors

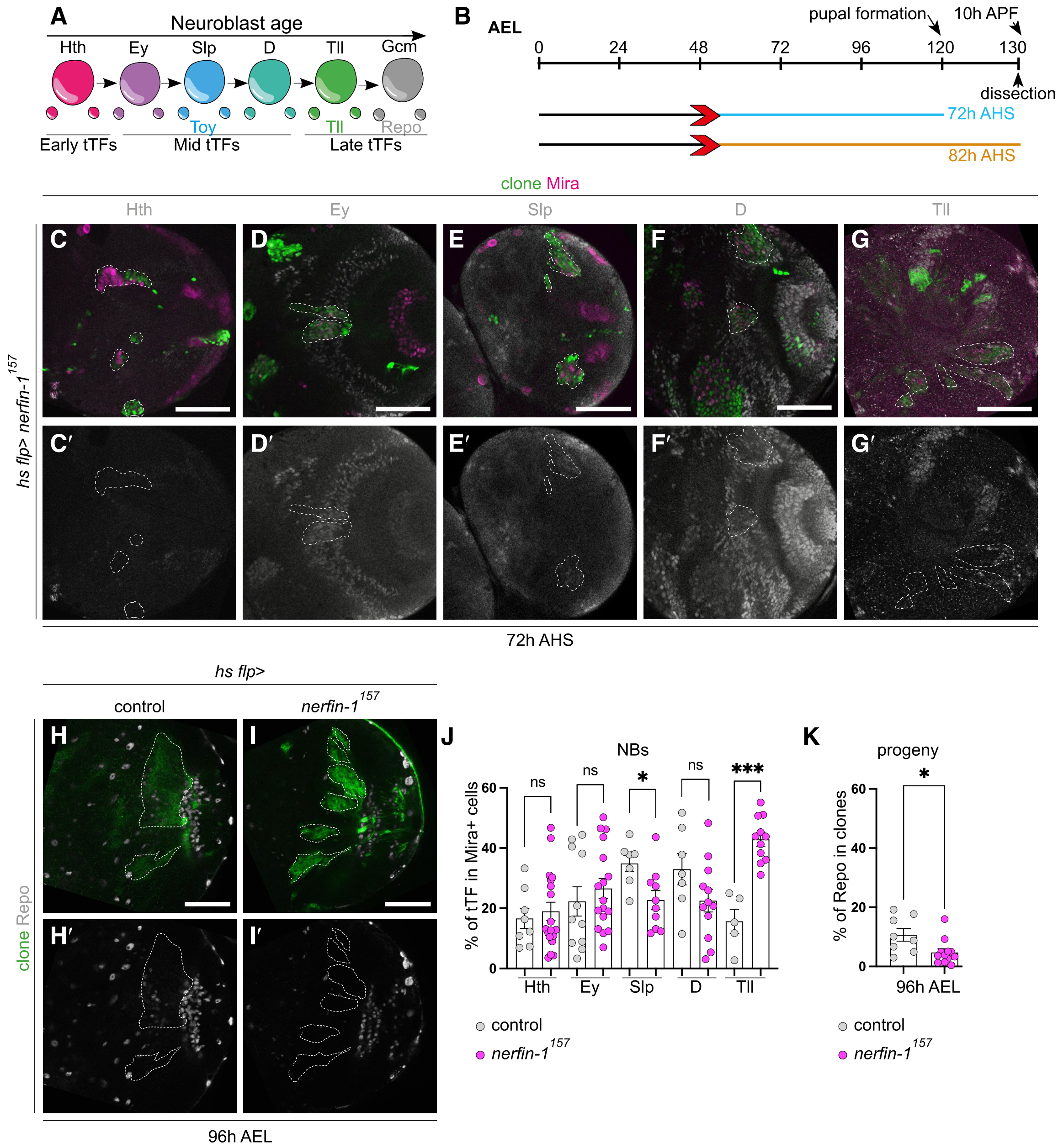
A
Neuroblast age
Hth Ey Slp D Tll Gcm
Toy
Tll Repo
Early tTFs
Mid tTFs
Late tTFs
B
AEL
0 24 48 72 96 120 130
pupal formation
10h APF
dissection
72h AHS
82h AHS
clone Mira
Hth Ey Slp D Tll
hs flp> nerfin-1 157
72h AHS
hs flp>
control
nerfin-1 157
clone Repo
96h AEL
J
NBs
% of tTF in Mira+ cells
ns ns * ns ***
Hth Ey Slp D Tll
control
nerfin-1 157
K
progeny
% of Repo in clones
96h AEL
control
nerfin-1 157

Figure EV5.

Figure EV5. Ectopic NBs generated via *nerfin-1* express excess Tll at the expense of Repo.

A Schematic representation of temporal series in neuroblasts (NBs). NBs express temporal transcription factors (tTFs) (Hth, Ey, Slp, D, Tll) as they age. These can be categorised into early, mid, and late tTFs.

B Schematic depicting the heat shock regimes used in (C–G′, H–K′). Clones were induced via heat shock (red arrows) and dissected at 72 h (96 h AEL, blue) or 82 h (10 h APF, orange) after heat shock.

C–I′ (C–G′, H–I′) Representative images of the deep medulla neuronal layer in the larval and pupal optic lobe, in which *nerfin-1*^{157} is driven in clones by *hs flp* (marked by GFP and outlined) and stained with the stem cell marker, Miranda (Mira, magenta), and various temporal transcription factors (tTFs) (grey). (C–G′) At 72 h AHS, in deep sections of the clone induced via *nerfin-1*^{157}, Mira$^+$ NBs express all temporal series transcription factors. Quantified in (J). (H–I′) At 72 and 82 h AHS, there is less Repo$^+$ (grey) progeny in *nerfin-1*^{157} mutant clones compared with the control. Quantified in (K).

J Quantification of volume of cells that express a specific tTF as % of total Mira$^+$ NB volume within a clone. Hth (Control, n = 8, m = 16.63 ± 3.335, *nerfin-1*^{157}, n = 18, m = 18.96 ± 3.083), Ey (Control, n = 11, m = 22.28 ± 4.833, *nerfin-1*^{157}, n = 17, m = 26.59 ± 3.32), Slp (Control, n = 7, m = 34.87 ± 2.753, *nerfin-1*^{157}, n = 10, m = 22.71 ± 3.18), D (Control, n = 7, m = 32.97 ± 5.22, *nerfin-1*^{157}, n = 12, m = 22.55 ± 3.768), Tll (Control, n = 5, m = 15.65 ± 4.036, *nerfin-1*^{157}, n = 11, m = 42.87, ±2.25).

K Quantification of % Repo$^+$ cells within control and *nerfin-1*^{157} mutant clones (calculated as the ratio of Repo$^+$ cell volume as a percentage of total clone volume). 72 h AHS (Control n = 8, m = 10.73 ± 2.148, *nerfin-1*^{157}, n = 12, m = 4.741 ± 1.224), 82 h AHS (Control n = 6, m = 28.64 ± 6.967, *nerfin-1*^{157}, n = 6, m = 8.246 ± 1.301).

Data information: Data are represented as mean ± SEM. P-values were obtained performing unpaired t-test, and Welch's correction was applied in case of unequal variances. *P < 0.05 and ***P < 0.001. Scale bars: 50 μm.

 © 2023 The Authors
