# Supplementary material for: Dedifferentiation‐derived neural stem cells exhibit perturbed temporal progression
Source: EMBO Rep. 2023 Apr 11;24(6):e55837. doi: 10.15252/embr.202255837 (PMC10240205; doi:10.15252/embr.202255837)
Supplement: Supplementary file 4 [file EMBR-24-e55837-s001.pdf]

EMBO reports

Dedifferentiation-derived neural stem cells exhibit perturbed temporal progression

Kellie Veen, Phuong-Khanh Nguyen, Francesca Froldi, Qian Dong, Edel Alvarez-Ochoa, Kieran Harvey, John McMullen, Owen Marshall, Patricia Jusuf, and Louise Cheng
DOI: 10.15252/embr.202255837

Corresponding author(s): Louise Cheng (louise.cheng@petermac.org)

Review Timeline:

Transfer from Review Commons:	22nd Jul 22
Editorial Decision:	29th Jul 22
Revision Received:	25th Jan 23
Editorial Decision:	2nd Mar 23
Revision Received:	6th Mar 23
Accepted:	20th Mar 23

Editor: Esther Schnapp

Transaction Report:

Review COMMONS

This manuscript was transferred to EMBO Reports following peer review at Review Commons.

(Note: With the exception of the correction of typographical or spelling errors that could be a source of ambiguity, letters and reports are not edited. Depending on transfer agreements, referee reports obtained elsewhere may or may not be included in this compilation. Referee reports are anonymous unless the Referee chooses to sign their reports.)

Review #1

1. Evidence, reproducibility and clarity:

Evidence, reproducibility and clarity (Required)

Summary

Authors show that overexpression of bHLH transcription factor Dpn in the medullary neurons of the Drosophila optic lobe results in the dedifferentiation of these neurons back into the NBs. These dedifferentiated NBs acquire and maintain mid-temporal identity, express Ey and Slp, and show delayed onset of tTF Tailless (Tll), leading to an excess of neurons of mid-temporal fate at the expense of late temporal fate neurons and glial cells. The dedifferentiated NBs are stalled in the cell cycle and fail to undergo terminal differentiation. Over expression of tTF Dicheate (D) or promoting G1/S transition pushed these NBs to late stages of the temporal series, partly rescuing the neuronal diversity and causing their terminal differentiation. They also show that the dedifferentiation of NBs by Notch hyper-activation also exhibited stalled temporal progression, which is restored by D overexpression.
Authors suggest that cell cycle regulation and tTF are primary to the proliferation and termination profile of dedifferentiated NBs.
Using these conclusions, the authors emphasize the need to recreate the right temporal profile and ensure appropriate cell cycle progression to use dedifferentiated NSC for regenerative purposes or prevent tumorigenesis originating from differentiated cell types.

Major comments:

- Are the key conclusions convincing?

Most conclusions are convincing; however, some issues are pointed out below.
- Should the authors qualify some of their claims as preliminary or speculative, or remove them altogether?

The authors have overexpressed Dpn and shown that medulla neurons dedifferentiate to NBs, similar to the loss of function phenotype seen for the Nerfin-1 of which Dpn is a target. They also show that temporal series progression defect is also seen in the case of dedifferentiated NB generated by Notch over-activation.
Using these two examples, the authors suggest that for dedifferentiated NSC, which are to be used for the regenerative purpose, one needs to recreate the right temporal

profile and ensure cell cycle progression occurs appropriately. Authors also claim that to prevent tumorigenesis originating from differentiated cell types, one needs to recreate the right temporal profile and ensure cell cycle progression occurs appropriately.

While I agree with this, I think this is an overreaching conclusion based on just these two examples. If they could show the same for one more method of dedifferentiation (For, e.g. Lola) happening in medulla neurons which happens by a mechanism independent of Nerfin-1, Dpn, Notch axis, the argument will become more convincing and broad.
Also when authors mention N mediated dedifferentiation, they need to inform that Dpn is a direct target of Notch in NBs (Doi. 10.1016/j.ydbio.2011.01.019), they do so in the discussion, but mentioning it here gives a broader context to the reader.

Another important point that needs a mentioned here is that conclusions are based on dedifferentiation happening in the medulla neurons, which are considered less stable since they lack Prospero. Therefore whether this conclusion can be generalized for all the tumors arising from dedifferentiation in the CNS (eg, those arising from NICD activation in the central brain or thoracic region of the VNC) is another concern. Maybe authors can consider making a more conservative claim.
Generalizing this conclusion to Prospero expressing NBs lies outside the scope of the current study and cannot be addressed here because central brain Type-I NBs use a different set of tTFs.
- Would additional experiments be essential to support the claims of the paper? Request additional experiments only where necessary for the paper as it is, and do not ask authors to open new lines of experimentation.

Experiments with Lola knockdown/mutants in medulla neurons can be done quickly, in my opinion, and will substantiate this claim.
Another obvious question that comes to mind is if medulla neurons dedifferentiate on overexpression of Dpn, does the same happen in nerfin-1 mutant clones as well? And if yes, why has the author not done similar experiments for nerfin-1 mutants.
Please show Ey staining in Fig-2 if possible, it will also help to add a line on why Slp was used as marker for mid tTFs instead of Ey.
In Model shown in last figure Dpn is shown to repress D and activate Slp. Can authors show that Dpn overexpression represses D and activate Slp either by antibody staining or by RT PCR.
- Are the suggested experiments realistic in terms of time and resources? It would help if you could add an estimated cost and time investment for substantial experiments.

Experiments with Lola and nerfin-1 mutants can be done in a few months. I cannot

comment on the cost involved.
- Are the data and the methods presented in such a way that they can be reproduced?

Yes
- Are the experiments adequately replicated and statistical analysis adequate?

Replication and statistical analysis are fine. The activated Notch experiments show only three data points in all the experiments. It will be good to increase this number.

Minor comments:

- Specific experimental issues that are easily addressable.

There is a problem with Fig-5F (both 5E and 5F have % EdU in clone/ % Mira in the clone as y-axis), I do not understand how the Fig-5F let them conclude that D overexpression increases the rate of neuronal production.

In one place, the authors conclude, "Together, this data suggests that it is likely that cell cycle progression lies upstream of the temporal series, to promote the generation of neurons". Authors should consider adding "medulla NBs" at the end of the sentence since cell cycle progression being upstream of temporal series is already known in Type-I NBs, as pointed out by authors as well (Ameele and Brand 2019).

In the discussion authors says that "Our data support the possible links between cell cycle progression and the expression of temporal regulators controlling NB proliferation and cellular diversity". This is new information, as the 2019 study did not show how cell diversity changes with a changed tTF profile. I think the authors should elaborate on this point to highlight how this is different from what is already known from the 2019 study (done in the context of Type-I NBs).
Maybe they need to highlight that the cell cycle directs/regulates the progression of temporal series compared to the earlier observation where temporal series was shown to be downstream of the cell cycle.

In fig-3J in clones even after 24 AHS, Dpn continues to be overexpressed but these cells undergo terminal differentiation, can authors comment why is it so?
In one place authors say, "To better assess the cumulative effect of the neurons made throughout development, EyOK107-GAL4 was used to drive the expression of Dpn" maybe some background on why use this specific GAL4.
Also a line about why GMR31HI08-GAL4 eyOK107-GAL4 and and eyR16F10-GAL4 were used.
- Are prior studies referenced appropriately ?

Yes, but in a few places, some references can be added.
An important point that needs to be mentioned for the context is the medulla neurons do not use Prospero for terminal differentiation and are thus considered less stable (DOI: 10.1242/dev.141341).
In discussion, the authors say that "It would be interesting to explore whether N similarly acts on these target genes to specify cell fate and proliferation profiles of dedifferentiated NBs." There is a study looking at Notch targets in NB hyperplasia (DOI: 10.1242/dev.126326); whether that study shows if any of the cell cycle genes are downstream of activated Notch, needs a mention here.
Also, when authors mention N mediated dedifferentiation, they need to inform that Dpn is a direct target of Notch in NBs (Doi. 10.1016/j.ydbio.2011.01.019). They do so in the discussion, but mentioning it in the introduction or results will give a broader context to the reader.
Another gene that needs a mention is "Brat", which regulates both Dpn and Notch, and causes dedifferentiation and tumors in CNS, I think this gene and its interaction with Dpn and Nerfin and Notch needs to be discussed either in the introduction or discussion.
- Are the text and figures clear and accurate?

The main figures are not labeled. Therefore, it was very annoying to deduce the specific figure numbers.
There are 1 or 2 places where figure calling is wrong in the text.
The Image Fig-5I shows cycD and CDK4 at the G2-M transition; while the text says it supports G1/S, which is indeed the case, the figure needs modification.
- Do you have suggestions that would help the authors improve the presentation of their data and conclusions?

The presentation is okay, in my opinion.

2. Significance:

Significance (Required)

- Describe the nature and significance of the advance (e.g. conceptual, technical, clinical) for the field.

The factors leading to dedifferentiation of the neurons have been identified previously by groups of Chris Doe (mldc, DOI: 10.1242/dev.093781), Andrea brand (10.1016/j.devcel.2014.01.030.) as well as the authors of this paper (10.1101/gad.250282.114, 10.1016/j.celrep.2018.10.038.). However, many questions

remained unaddressed regarding such NB generated from neuronal dedifferentiation. For example, whether these cells contribute to native cell diversity of the CNS, undergo timely differentiation or their progeny cells incorporated into appropriate circuits is not well understood. Successful execution of these phenomena is critical for generating functional CNS and such insights are crucial for understanding the origin of tumorigenesis in CNS or employing dedifferentiated NSC for regenerative purposes.

This study is an overexpression-based study, however, some of the results give significant conceptual insights into the tumors arising out of the dedifferentiation of the neurons. It also gives insights into the fact that the dedifferentiated cells need to be carefully examined for the temporal factor profile before they can be employed for regeneration or any therapy targeting them.
However, in my opinion, they need to test this idea at least in one more system of neuronal dedifferentiation, preferably independent of the nerfin-1/Notch/Dpn axis to generalize this claim.
- Place the work in the context of the existing literature (provide references, where appropriate).

Cerdic Maurange's group had looked at the role of temporal factors and identified the early phase of malignant susceptibility in Drosophila in 2016 (doi: 10.7554/eLife.13463). Andrea Brand's group has shown in a 2019 paper that cell cycle progression is essential for temporal transition in NBs (doi: 10.7554/eLife.47887). Both these studies were in the context of Type-I NBs, which express Prospero, which is crucial for the differentiation of the neurons.
Previously the authors have studied type-I NBs and shown by Targeted DamID that Dpn is Nerfin-1 target. They also show that Nerfin-1 mutants show dedifferentiation of neurons. They follow up on this observation in medulla neurons, where they find that Dpn overexpression results in their dedifferentiation into medulla NBs. Medulla NBs differ from Type-I NBs in using a separate set of tTFs. Also, Type-I NB and neurons arising from them use Prospero for terminal differentiation, while medulla neurons do not express Prospero and are therefore considered less stable (DOI: 10.1242/dev.141341).

The importance of the study lies in the results that show that the NB arising out of dedifferentiation of medulla neurons takes up mid-temporal fate. These NBs are stalled in Slp expressing mid-temporal stage unless the cell cycle is promoted by overexpression of cell cycle genes regulating G1/S transition.
Authors also show that overexpression of D promotes the progression of temporal series in these dedifferentiated NBs, which could partly rescue neuronal diversity and result in terminal differentiation. Thus D plays an important role in determining the

type of neurons these NBs generated. This suggests that knowing the tTF profile of these types of dedifferentiated NBs is vital if these cells were to be used for regenerative purposes. Authors further claimed that cell cycle regulation and tTFs are critical determinants of the proliferation and termination profile of dedifferentiated NBs.
- State what audience might be interested in and influenced by the reported findings.

The study will be of broader interest to researchers interested in central nervous system patterning, regeneration, and cancer biology.
- Define your field of expertise with a few keywords to help the authors contextualize your point of view. Indicate if there are any parts of the paper that you do not have sufficient expertise to evaluate.

Drosophila, central nervous system patterning and cell fate determination of neural stem cells.

3. How much time do you estimate the authors will need to complete the suggested revisions:

Estimated time to Complete Revisions (Required)

(Decision Recommendation)

Between 3 and 6 months

4. Review Commons values the work of reviewers and encourages them to get credit for their work. Select 'Yes' below to register your reviewing activity at Publons; note that the content of your review will not be visible on Publons.

Reviewer Publons

Yes

Review #2

1. Evidence, reproducibility and clarity:

Evidence, reproducibility and clarity (Required)

Stem cells can divide asymmetrically to self-renew the stem cell while generating differentiating sibling cells. To restrict the number and type of differentiating sibling cells, stem cells often undergo terminal differentiation. Terminally differentiated cells can dedifferentiate and revert to a stem cell like fate. However, the underlying molecular mechanisms are incompletely understood in vivo.
Here, Veen et al., use Drosophila neural stem cells (called neuroblasts) to investigate how terminal differentiation is regulated. Neuroblasts faithfully produce the correct number and type of neuronal cells through temporal patterning and regulated terminal differentiation. The authors show that misexpression of the bHLH transcription factor Deadpan (Dpn) induces ectopic neuroblasts, which predominantly express mid-temporal transcription factors at the expense of late-temporal transcription factors. As a consequence, these ectopic neuroblasts also fail to produce Repo positive glial cells and are stalled in their cell cycle progression. The authors provide evidence that promoting cell cycle progression and overexpression of the transcription factor Dichaete (D) is sufficient to restore the temporal transcription factor series, neuronal diversity and timely neuroblast differentiation.

This is an interesting study that will be of interest to the stem cell field. However, I encourage the authors to consider the following critiques:

1. Explain the rationale for the three different neuronal/NB drivers (GMR31HI08-GAL4, eyOK107-GAL4, eyR16F10-GAL4. How are they expressed?
2. The rationale for the Edu experiment (Figure S1I) is not clear. Why is this a measure for the production of neuronal progeny? For the correct interpretation of these results, the authors should also provide control clones or Edu experiments of regular neuroblasts.
3. How was % of Mira (Figure 1K and below) or the % of tTFs (Figure 2H onward) quantified? For instance, Figure 2C-G often shows clonal signal that is not highlighted with the dashed lines and the corresponding tTF intensity does not match the intensity in the outlined clone (eg. Figure 2D-D'; a large optic lobe clone is negative for Ey. Figure 2E-E'; an unmarked clone is negative for Slp).
Similarly, the Hth signal is very weak to begin with so it is unclear how this was quantified. How was determined what constitutes real signal vs. background noise? Additional explanations in the methods section is needed to assess the robustness of

the data.
4. This sentence should be rephrased: 'As the tumour cell-of-origin can define the competence of tumour NBs to undergo malignancy (Farnsworth et al., 2015; Narbonne-Reveau et al., 2016), we next tested whether the temporal identity of the dedifferentiated NBs were conferred by the age of the neurons they were derived from.'
The connection between tumorigenicity and temporal identity is not really clear and should be briefly reintroduced for this paragraph.
5. Figure 2I-N: The experimental outline in I and J should be grouped with the corresponding images to clarify what is compared. Also, there are no images for the control clones, which make a comparison difficult. The images are also too small. I cannot really see the Hth, or Slp signal in the small clones shown in Figure 2K-L".
6. Figure 3H: It is not clear why there are only a small group of Nbs that are positive for Mira. Please explain.
7. Figure 3K-M: Please explain how the Toy signal was measured and quantified.
8. The TaDa data set is very interesting but the following might be an overstatement: "We found that Dpn directly binds to slp1 as well as the Sox-family TF dichaete (D) which is expressed in medulla NBs after slp1 (Li et al., 2013) (Figure S6 A-B)." More direct binding assays might be needed to show that Dpn directly binds to slp1 and D. If this is already shown, clarify the sentence to indicate what is published and what is extracted from the data shown here.
Also, what is the rationale for this statement: "Consistent with the model that D represses Slp-1..."?
9. This might be an overinterpretation: D overexpression in UAS-Dpn NBs promoted their pre-mature cell cycle exit at 6 hrs APF using eyR16F10-GAL4. The data shows loss of Mira signal, which could occur through different mechanisms.

2. Significance:

Significance (Required)

These appear to be novel and significant findings that will enhance our understanding of the temporal progression and terminal differentiation program of neural stem cells in vivo.
I think the findings will be of interest to cell, developmental cell and stem cell biologists.

My primary expertise is in the cell biology of fly neural stem cells and asymmetric cell division of neuroblasts. Although I am not intimately familiar with the differentiation and differentiation literature, I consider the findings reported here relevant and impactful.

3. How much time do you estimate the authors will need to complete the suggested revisions:

Estimated time to Complete Revisions (Required)

(Decision Recommendation)

Between 1 and 3 months

4. Review Commons values the work of reviewers and encourages them to get credit for their work. Select 'Yes' below to register your reviewing activity at Publons; note that the content of your review will not be visible on Publons.

Reviewer Publons

Yes

Review #3

1. Evidence, reproducibility and clarity:

Evidence, reproducibility and clarity (Required)

The discoveries that the author describe in this manuscript are very specific to dedifferentiated neuroblasts created by UAS-dpn transgene overexpression. Dpn is endogenously expressed in optic lobe neuroblast throughout larval stage, which makes understanding how Dpn regulates gene expression based on the authors results (suppression of cell-cycle genes, and promotion of a specific temporal state) confusing. Therefore, this manuscript does not advance our understanding of regulation of temporal identity and cell cycle progression in optic lobe neuroblasts during normal neurogenesis.

The author's state:

"However, beyond the fact that misexpression of these factors and pathways caused the formation of ectopic NBs, whether these dedifferentiated NBs faithfully produce the correct number and types of neurons or glial cells, or undergo timely terminal differentiation, has not been assessed. These characteristics are key determinants of overall CNS size and function, thus are important parameters when considering whether dedifferentiation leads to tumourigenesis or can be appropriately utilized for regenerative purposes."
at the end of introduction. If this is a true primary goal of this study, the authors should describe it in abstract. Otherwise, readers will lose enthusiasm to read this manuscript in abstract and no longer read the following sections.

Results

1. The authors should describe the expression pattern of all three of the Gal4 drivers used. While there are dotted outlines in the supplemental figure, there should be a description in the main text for the expression pattern of these lines which described with temporal state of NBs these lines are expressed in, and whether they are also expressed in the neurons or not.
2. The authors claim that overexpression of Dpn in the medulla region causes "dedifferentiation." The data provided however is not sufficient to conclude that dedifferentiation is occurring. The GAL4s used all drive in the NBs, and so it is unclear if the ectopic NBs ever became mature neurons. In addition, the lack of ectopic NBs in the clonal analysis 16hrs AHS does not prove that ectopic NBs at 24hrs AHS must have come from "mature neurons." To demonstrate dedifferentiation, the authors should use a driver system that is specific to mature neurons, and then overexpress dpn and look for mira+ cells. Currently, the authors data does not prove that mature neurons dedifferentiatiate into ectopic NBs upon Dpn OE.
3. What is a conclusion of fig 2C-H?
4. "As the tumor cell-of-origin can define the competence of tumor NBs to undergo malignancy identity of the dedifferentiated NBs were conferred by the age of the neurons they were derived from". This sentence is confusing. What are the authors investigating in the following experiment? Do they want to see ectopic NBs keep their early identity like Chinmo in ventral cord tumor NB? Or tll-positive NB's progenies can dedifferentiate to ectopic NB, but this ectopic neuroblast is not able to keep proliferation in pupal stage? It is hard to understand the connection of this sentence and the following experiment.
5. The DamID experiment described used wor-gal4 as a driver, which means the Dpn binding profile generated is coming from not only optic lobe NBs, but central brain NBs and VNC NBs as well. In Magadi et al. (2020), the authors profiled Dpn binding in CNS hyperplasia, and found that dpn strongly bound Nerfin-1 and gcm. However, it does not bind cell cycle genes in this context. How do the authors know that the

region that they claim are bound by dpn are bound in medulla NBs? The authors should also include tracks to show dpn binding at Nerfin-1, as well as the other tTFs (hth, ey, tll, and gcm). Providing this data will help to understand if Dpn binding is specific to the mid-temporal genes, as Dpn expression is known to be expressed in all medulla NBs regardless of temporal state.
6. Currently, the DamID data does not help to interpret the Dpn overexpression phenotype at all. Inside of flip-out clone, some cells show Slp-1 expression while others showed D expression. The authors explain that Slp-1 and D suppress their expression to each other. But the DamID data indicate that both Slp-1 and D are Dpn target genes. If this is true, why did they observe the mosaic expression pattern inside of the same clone.
7. The authors hypothesized if Dpn activated Slp-1directly. Does this mean that Dpn directly activate transcription of Slp-1? It is well known that Dpn is transcriptional repressor. Hes family proteins form a homodimer or heterodimer with another Hes protein and interacts Gro, which recruits a Histon deacetylase protein. The author's claim does not fit to the model what we currently believe. In addition, the authors claimed that Dpn inhibits cell cycle gene transcription directly. This is inconsistent to their claim that Dpn directly activate Slp-1 expression. If the authors want to claim that Dpn has two different functions in this context, the authors must demonstrate it by experimental results.
8. Related to the above question, I wondered if the authors guess Dpn activate or repress D transcription by binding to D promoter region because they claimed that Dpn activate Slp-1, while suppress cell cycle genes.
9. I am confused to the claim that Dpn suppress cell cycle genes expression. Dpn overexpression induces dedifferentiation of neuron into NB and re-entry into the cell cycle. If Dpn suppress cell cycle genes how can the dedifferentiated cell re-enter into the cell cycle?
10. Figure 6 looked redundant because we know Dpn is a direct target of Notch. It is obvious that an upstream factor overexpression can induce the identical phenotype to the phenotype induced by overexpression of a downstream factor.

Minor comments:

1. Typo in main text: "GMR31HI08-GAL4" should be "GMR31H08-GAL4"
2. In figure 1E-H the dotted line regions indicated the clones are not shown in the merge image. Please include
3. Typo in discussion paragraph 2: "temporal series was no sufficient to rescue cycle cycle progression"

2. Significance:

Significance (Required)

Insights into the developmental capacity of dedifferentiated stem cells will likely lead to novel strategy to replenish cells lost due to aging, injury and diseases in regenerative medicine.

3. How much time do you estimate the authors will need to complete the suggested revisions:

Estimated time to Complete Revisions (Required)

(Decision Recommendation)

More than 6 months

4. Review Commons values the work of reviewers and encourages them to get credit for their work. Select 'Yes' below to register your reviewing activity at Publons; note that the content of your review will not be visible on Publons.

Reviewer Publons

Yes

Revision Plan

Manuscript number: #RC-2022-01506
Corresponding author(s): Louise Cheng

[The "revision plan" should delineate the revisions that authors intend to carry out in response to the points raised by the referees. It also provides the authors with the opportunity to explain their view of the paper and of the referee reports.

The document is important for the editors of affiliate journals when they make a first decision on the transferred manuscript. It will also be useful to readers of the reprint and help them to obtain a balanced view of the paper.

If you wish to submit a full revision, please use our "Full Revision" template. ***It is important to use the appropriate template to clearly inform the editors of your intentions.****]*

1. General Statements [optional]

The goal of the paper is to establish whether dedifferentiated neural stem cells derived from overexpression of Hes family transcription factor Deadpan and Notch (as well as other transcription factors such as Lola and Nerfin-1) can faithfully differentiate, produce correct types/diversity of neurons, and undergo appropriate terminal differentiation, important characteristics of neural stem cells that can be utilised for regenerative purposes.

2. Description of the planned revisions

Insert here a point-by-point reply that explains what revisions, additional experimentations and analyses are planned to address the points raised by the referees.

Reviewer #1

While I agree with this … If they could show the same for one more method of dedifferentiation (For, e.g. Lola) happening in medulla neurons which happens by a mechanism independent of Nerfin-1, Dpn, Notch axis, the argument will become more convincing and broad. We will characterise the temporal identity, termination and cellular identity of Lola-Ri induced ectopic neuroblasts. If these parameters are disrupted, we will overexpress D to assess whether this can trigger the progression of the temporal series.

Also when authors mention N mediated dedifferentiation, they need to inform that Dpn is a direct target of Notch in NBs (Doi. 10.1016/j.ydbio.2011.01.019), they do so in the discussion, but mentioning it here gives a broader context to the reader.
We will include that Dpn is a target of Notch when first mentioned.

Another important point that needs a mentioned here is that conclusions are based on dedifferentiation happening in the medulla neurons, which are considered less stable since they lack Prospero. Therefore whether this conclusion can be generalized for all the tumors arising from dedifferentiation in the CNS (eg, those arising from NICD activation in the central brain or thoracic region of the VNC) is another concern. Maybe authors can consider making a more conservative claim.
Generalizing this conclusion to Prospero expressing NBs lies outside the scope of the current study and cannot be addressed here because central brain Type-I NBs use a different set of tTFs.
We will make a more conservative claim and clarify all of our conclusion are medulla neuron-specific.

Another obvious question that comes to mind is if medulla neurons dedifferentiate on overexpression of Dpn, does the same happen in nerfin-1 mutant clones as well? And if yes, why has the author not done similar experiments for nerfin-1 mutants.
We will assess the temporal identity of neuroblasts in nerfin-1 mutant clones.

Please show Ey staining in Fig-2 if possible, it will also help to add a line on why Slp was used as marker for mid tTFs instead of Ey.
Ey is shown in Fig-2 (D-D'') already. Slp is used as a marker of mid tTFs as Ey is expressed also in neurons thus would also be present in deep sections of control clones, whereas Slp is not expressed in neurons. We therefore used Slp as a proxy for mid-temporal identity throughout our study. We will include this text in our revision.

In Model shown in last figure Dpn is shown to repress D and activate Slp. Can authors show that Dpn overexpression represses D and activate Slp either by antibody staining or by RT PCR.
In Figure 2H, we have shown in clones that overexpression of Dpn induced a significant increase of Slp. In Figure S3B-B'', we have shown that Dpn overexpression causes an upregulation of Slp at 6 hr APF. We can think we have pretty convincingly shown that Dpn overexpression activates Slp.

For Dichaete, our existing data shows that Dpn overexpression did not significantly alter D expression. To assess if using a stronger driver might allow us to see some changes, we will induced dedifferentiation via Dpn overexpression using the Eyeless-Gal4 driver. In this experiment, we will quantify the amount of D upon Dpn overexpression. Depending on this result, we will revise our conclusion on whether Dpn overexpression represses D.

The activated Notch experiments show only three data points in all the experiments. It will be good to increase this number.
We will repeat Notch experiments to increase the n number for these experiments.

Minor comments:
- Specific experimental issues that are easily addressable.
There is a problem with Fig-5F (both 5E and 5F have % EdU in clone/ % Mira in the clone as y-axis), I do not understand how the Fig-5F let them conclude that D overexpression increases the rate of neuronal production.
In the text we said: "We found that D overexpression **did not** significantly increase neuronal production, suggesting that it is likely that cell cycle progression lies upstream or in parallel to the temporal series, to promote the generation of neurons."

In one place, the authors conclude, "Together, this data suggests that it is likely that cell cycle progression lies upstream of the temporal series, to promote the generation of neurons". Authors should consider adding "medulla NBs" at the end of the sentence since cell cycle progression being upstream of temporal series is already known in Type-I NBs, as pointed out by authors as well (Ameele and Brand 2019).
We will add "medulla NBs" to the end of this sentence.

In the discussion authors says that "Our data support the possible links between cell cycle progression and the expression of temporal regulators controlling NB proliferation and cellular diversity". This is new information, as the 2019 study did not show how cell diversity changes with a changed tTF profile. I think the authors should elaborate on this point to highlight how this is different from what is already known from the 2019 study (done in the context of Type-I NBs).
Maybe they need to highlight that the cell cycle directs/regulates the progression of temporal series compared to the earlier observation where temporal series was shown to be downstream of the cell cycle.
We will expand in discussion to discuss the link between cell cycle/tTFs.

In fig-3J in clones even after 24 AHS, Dpn continues to be overexpressed but these cells undergo terminal differentiation, can authors comment why is it so?
While Dpn is overexpressed, it progresses through the temporal series at a slower pace due to a delay in cell cycle progression, as well as delayed onset of D, these NBs still eventually reach the terminal temporal identity, and are thus about to undergo terminal differentiation. We will include an additional piece of data that shows NBs induced by Dpn overexpression do eventually turn on Tll.

In one place authors say, "To better assess the cumulative effect of the neurons made throughout development, EyOK107-GAL4 was used to drive the expression of Dpn" maybe some background on why use this specific GAL4.

Also a line about why GMR31HI08-GAL4 eyOK107-GAL4 and and eyR16F10-GAL4 were used.
We will include an expression analysis of EyOK107-GAL4 and eyR16F10-GAL4. GMR31HI08-GAL4 expression analysis was previously published (Vissers et al., 2018). We will explain in the text the benefits of each driver.

- Are prior studies referenced appropriately ?
Yes, but in a few places, some references can be added.
An important point that needs to be mentioned for the context is the medulla neurons do not use Prospero for terminal differentiation and are thus considered less stable (DOI: 10.1242/dev.141341).
We beg to disagree with the reviewer in terms of Pros is not required for terminal differentiation of medulla neuroblasts. Li et al., 2013 shows that nuclear Pros is found in the oldest NBs. We do agree that differentiated state of medulla neurons is less stable, possibly owing to absence of Pros, and we will include that in our discussion.

In discussion, the authors say that "It would be interesting to explore whether N similarly acts on these target genes to specify cell fate and proliferation profiles of dedifferentiated NBs." There is a study looking at Notch targets in NB hyperplasia (DOI: 10.1242/dev.126326); whether that study shows if any of the cell cycle genes are downstream of activated Notch, needs a mention here.
We will discuss the study looking at N targets in NB hyperplasia in the discussion of the revised manuscript.

Also, when authors mention N mediated dedifferentiation, they need to inform that Dpn is a direct target of Notch in NBs (Doi. 10.1016/j.ydbio.2011.01.019). They do so in the discussion, but mentioning it in the introduction or results will give a broader context to the reader.
We will mention that Dpn is a target of Notch in the results section.

Another gene that needs a mention is "Brat", which regulates both Dpn and Notch, and causes dedifferentiation and tumors in CNS, I think this gene and its interaction with Dpn and Nerfin and Notch needs to be discussed either in the introduction or discussion.
We will comment on Brat in the discussion.

- Are the text and figures clear and accurate?
The main figures are not labeled. Therefore, it was very annoying to deduce the specific figure numbers.
We will label the figures.

There are 1 or 2 places where figure calling is wrong in the text.

We will check and correct all call-outs.

The Image Fig-5I shows cycD and CDK4 at the G2-M transition; while the text says it supports G1/S, which is indeed the case, the figure needs modification.
We will modify the cell cycle figure and cdk4/cycd to g1/s.

Reviewer #2 (Evidence, reproducibility and clarity (Required)):

1. Explain the rationale for the three different neuronal/NB drivers (GMR31HI08-GAL4, eyOK107-GAL4, eyR16F10-GAL4. How are they expressed?
We will include an expression analysis of EyOK107-GAL4 and eyR16F10-GAL4. GMR31HI08-GAL4 expression analysis was previously published (Vissers et al., 2018). We will explain in the text the benefits of each driver.

2. The rationale for the Edu experiment (Figure S1I) is not clear. Why is this a measure for the production of neuronal progeny? For the correct interpretation of these results, the authors should also provide control clones or Edu experiments of regular neuroblasts.
We will repeat this experiment and mark the progeny with the neuronal marker Elav, to demonstrate that they are neurons. Additionally, we will add the control to this figure.

3. How was % of Mira (Figure 1K and below) or the % of tTFs (Figure 2H onward) quantified? For instance, Figure 2C-G often shows clonal signal that is not highlighted with the dashed lines and the corresponding tTF intensity does not match the intensity in the outlined clone (eg. Figure 2D-D'; a large optic lobe clone is negative for Ey. Figure 2E-E'; an unmarked clone is negative for Slp).
Similarly, the Hth signal is very weak to begin with so it is unclear how this was quantified. How was determined what constitutes real signal vs. background noise?
Additional explanations in the methods section is needed to assess the robustness of the data.
We will expand the methods section and mention that we used similar thresholding in antibody staining between control and uas dpn in all instances, so even if the antibody is weaker (eg hth) it is consistently quantified. Additionally, we can increase the intensity of Ey in Figure 2D-2D’, as it is expressed at low levels.

4. This sentence should be rephrased: 'As the tumour cell-of-origin can define the competence of tumour NBs to undergo malignancy (Farnsworth et al., 2015; Narbonne-Reveau et al., 2016), we next tested whether the temporal identity of the dedifferentiated NBs were conferred by the age of the neurons they were derived from.'
The connection between tumorigenicity and temporal identity is not really clear and should be briefly reintroduced for this paragraph.

We will rephrase this sentence and further introduce this concept when talking about tumour cell of origin and competence.

5. Figure 2I-N: The experimental outline in I and J should be grouped with the corresponding images to clarify what is compared. Also, there are no images for the control clones, which make a comparison difficult. The images are also too small. I cannot really see the Hth, or Slp signal in the small clones shown in Figure 2K-L".
We will split figure 2 into two images. The first image including A-H and the control data. And the second including I-Q and the control data. This will increase the size of the images. Additionally, we will group I and J with corresponding data.

6. Figure 3H: It is not clear why there are only a small group of Nbs that are positive for Mira. Please explain.
Most NBs have terminated by this time point, we will explain this within the text.

7. Figure 3K-M: Please explain how the Toy signal was measured and quantified.
We will expand the methods section and explain how Toy quantification is made.

8. The TaDa data set is very interesting but the following might be an overstatement: "We found that Dpn directly binds to slp1 as well as the Sox-family TF dichaete (D) which is expressed in medulla NBs after slp1 (Li et al., 2013) (Figure S6 A-B)."
More direct binding assays might be needed to show that Dpn directly binds to slp1 and D. If this is already shown, clarify the sentence to indicate what is published and what is extracted from the data shown here.
The DamID data do actually show that Dpn binds (i.e. there is a statistically significant peak at FDR<0.01) directly at these loci (see the TaDa supp fig A & B). Whether it's doing anything functional or not, we can't say, but our data shows that Dpn directly binds to slp1 and D. We will clarify the sentence to indicate this in our revision.

Also, what is the rationale for this statement: "Consistent with the model that D represses Slp-1..."?
We will include an explanation and references of studies that have produced this model.

9. This might be an overinterpretation: D overexpression in UAS-Dpn NBs promoted their pre-mature cell cycle exit at 6 hrs APF using eyR16F10-GAL4. The data shows loss of Mira signal, which could occur through different mechanisms.
Our data already shows that these NBs express Tll, the terminal temporal transcription factor (Figure 4F). In addition, we show that there is an increase in Tll+ and Repo+ progeny (Figure 4K, L). Together, this suggests that D overexpression promotes the progression of the temporal

series. However, it is possible that Mira+ cells can disappear via cell death. We will assess this possibility by staining for cell death marker Dcp1 at 6hr APF.

Reviewer #3 (Evidence, reproducibility and clarity (Required)):

The discoveries that the author describe in this manuscript are very specific to dedifferentiated neuroblasts created by UAS-dpn transgene overexpression. Dpn is endogenously expressed in optic lobe neuroblast throughout larval stage, which makes understanding how Dpn regulates gene expression based on the authors results (suppression of cell-cycle genes, and promotion of a specific temporal state) confusing.

Our data relate specifically to gene regulation by Dpn in a dedifferentiated context, and do not seek to understand Dpn regulation in wt neuroblasts. The reviewer is assuming our scope is greater here: we're not trying to claim that we know what Dpn is doing in wt NBs, and it's not surprising that ectopic effects in neurons may be different to wt NBs.

To assess whether the mechanisms described apply to more than Dpn overexpression, we will also assess whether the temporal series progression is affected in Lola RNAi and Nerfin-1 mutant.

Therefore, this manuscript does not advance our understanding of regulation of temporal identity and cell cycle progression in optic lobe neuroblasts during normal neurogenesis.
The author's state:
"However, beyond the fact that misexpression of these factors and pathways caused the formation of ectopic NBs, whether these dedifferentiated NBs faithfully produce the correct number and types of neurons or glial cells, or undergo timely terminal differentiation, has not been assessed. These characteristics are key determinants of overall CNS size and function, thus are important parameters when considering whether dedifferentiation leads to tumourigenesis or can be appropriately utilized for regenerative purposes."
at the end of introduction. If this is a true primary goal of this study, the authors should describe it in abstract
We will add this to the abstract.

Results
1. The authors should describe the expression pattern of all three of the Gal4 drivers used. While there are dotted outlines in the supplemental figure, there should be a description in the main text for the expression pattern of these lines which described with temporal state of NBs these lines are expressed in, and whether they are also expressed in the neurons or not.

We will include expression analysis of all three drivers in a supplementary figure and explain in the text the benefit of each driver.

2. The authors claim that overexpression of Dpn in the medulla region causes "dedifferentiation." The data provided however is not sufficient to conclude that dedifferentiation is occurring. The GAL4s used all drive in the NBs, and so it is unclear if the ectopic NBs ever became mature neurons. In addition, the lack of ectopic NBs in the clonal analysis 16hrs AHS does not prove that ectopic NBs at 24hrs AHS must have come from "mature neurons." To demonstrate dedifferentiation, the authors should use a driver system that is specific to mature neurons, and then overexpress dpn and look for mira+ cells. Currently, the authors data does not prove that mature neurons dedifferentiatiate into ectopic NBs upon Dpn OE.
We have conducted lineage tracing (G-Trace) analysis of the medulla neuron driver GMR31H08-GAL4 which we utilise in our study, this driver is predominantly expressed within the medulla neurons (real time) except for a few GMCs present in the lineage. Therefore, the Mira positive cells induced via Dpn overexpression are most likely from dedifferentiation (We will include this data in a supplemental figure in our revised manuscript).
To further support this, we will use GMR31H08-GAL4 with a Gal80ts, to restrict the timing to dedifferentiation induction to 3rd instar, so that the driver is restricted to neurons. Similar strategy to induce dedifferentiation was utilised in DOI: 10.1242/dev.141341 and DOI: 10.1016/j.devcel.2014.01.030.

3. What is a conclusion of fig 2C-H?
Fig 2C-H assess the expression of tTFs in UAS-dpn induced ectopic NBs. We will make these conclusions clearer in the text.

4. "As the tumor cell-of-origin can define the competence of tumor NBs to undergo malignancy identity of the dedifferentiated NBs were conferred by the age of the neurons they were derived from". This sentence is confusing. What are the authors investigating in the following experiment? Do they want to see ectopic NBs keep their early identity like Chinmo in ventral cord tumor NB? Or tll-positive NB's progenies can dedifferentiate to ectopic NB, but this ectopic neuroblast is not able to keep proliferation in pupal stage? It is hard to understand the connection of this sentence and the following experiment.
We will rephrase this sentence and further introduce this concept when talking about tumour cell of origin and competence. Additionally, we will make the connection to the experiments which follow it clearer.

5. The DamID experiment described used wor-gal4 as a driver, which means the Dpn binding profile generated is coming from not only optic lobe NBs, but central brain NBs and VNC NBs as well. In Magadi et al. (2020), the authors profiled Dpn binding in CNS hyperplasia, and found that dpn strongly bound Nerfin-1 and gcm. However, it does not bind cell cycle genes in this

context. How do the authors know that the region that they claim are bound by dpn are bound in medulla NBs?

We agree with the reviewer that the profile is not specific to medulla NBs. To assess Dpn binding profiles specifically in the medulla NBs, we will use the recently-published NanoDam technique (https://doi.org/10.1016/j.devcel.2022.04.008) for profiling GFP-fusion proteins, with a medulla specific driver (eyR16F10-GAL4) and Dpn-GFP (recombineered locus under endogenous control). This should inform us whether the target genes we have identified are relevant in the medulla.

The authors should also include tracks to show dpn binding at Nerfin-1, as well as the other tTFs (hth, ey, tll, and gcm). Providing this data will help to understand if Dpn binding is specific to the mid-temporal genes, as Dpn expression is known to be expressed in all medulla NBs regardless of temporal state.

We will include the tracks of the other transcription factors.

6. Currently, the DamID data does not help to interpret the Dpn overexpression phenotype at all. Inside of flip-out clone, some cells show Slp-1 expression while others showed D expression. The authors explain that Slp-1 and D suppress their expression to each other. But the DamID data indicate that both Slp-1 and D are Dpn target genes. If this is true, why did they observe the mosaic expression pattern inside of the same clone.

We observed that high levels of Slp-1 is correlated with low levels of D. This suggest to us that the initial stochastic differences accounts for where Slp-1 is high is where D is low, and vice versa.

7. The authors hypothesized if Dpn activated Slp-1directly. Does this mean that Dpn directly activate transcription of Slp-1? It is well known that Dpn is transcriptional repressor. Hes family proteins form a homodimer or heterodimer with another Hes protein and interacts Gro, which recruits a Histon deacetylase protein. The author's claim does not fit to the model what we currently believe. In addition, the authors claimed that Dpn inhibits cell cycle gene transcription directly. This is inconsistent to their claim that Dpn directly activate Slp-1 expression. If the authors want to claim that Dpn has two different functions in this context, the authors must demonstrate it by experimental results.

We will discuss these models in the Discussion, and make our claims more conservative, as we do not have direct experimental evidence to prove or disprove the model that Dpn is acting as an activator in this context.

8. Related to the above question, I wondered if the authors guess Dpn activate or repress D transcription by binding to D promoter region because they claimed that Dpn activate Slp-1, while suppress cell cycle genes.

We will make our claims more conservative, and discuss this point further in the Discussion.

9. I am confused to the claim that Dpn suppress cell cycle genes expression. Dpn overexpression induces dedifferentiation of neuron into NB and re-entry into the cell cycle. If Dpn suppress cell cycle genes how can the dedifferentiated cell re-enter into the cell cycle?
The data points towards that Dpn overexpression has two separate roles in regulating the cell cycle. Ofcourse dedifferentiation requires a commitment of neurons into the cell cycle (this we think is still happening), however, we think once these cells have turned on NB markers, they have limited ability to progress through the cell cycle. We will discuss this point in the Discussion.

10. Figure 6 looked redundant because we know Dpn is a direct target of Notch. It is obvious that an upstream factor overexpression can induce the identical phenotype to the phenotype induced by overexpression of a downstream factor.
A direct target does not necessarily infer the same phenotype. To assess whether the mechanisms apply to other dedifferentiation models, we will add Lola-RNAi and Nerfin-1 data to our revised manuscript.

Minor comments:
1. Typo in main text: "GMR31HI08-GAL4" should be "GMR31H08-GAL4"
We will adjust this in the text.

2. In figure 1E-H the dotted line regions indicated the clones are not shown in the merge image. Please include
We will include this.

3. Typo in discussion paragraph 2: "temporal series was no sufficient to rescue cycle cycle progression"
We will fix this.

3. Description of the revisions that have already been incorporated in the transferred manuscript

Please insert a point-by-point reply describing the revisions that were already carried out and included in the transferred manuscript. If no revisions have been carried out yet, please leave this section empty.

Revision Plan

4. Description of analyses that authors prefer not to carry out

Please include a point-by-point response explaining why some of the requested data or additional analyses might not be necessary or cannot be provided within the scope of a revision. This can be due to time or resource limitations or in case of disagreement about the necessity of such additional data given the scope of the study. Please leave empty if not applicable.

1st Editorial Decision **29th Jul 2022**

Dear Dr. Cheng,

Thank you for the submission of your manuscript plus your revision plan to EMBO reports. We have now read the referee comments and discussed your proposed revisions, and we agree with your plan, and that the revised study overall could be a good fit for EMBO reports.

I would thus like to invite you to revise your manuscript with the understanding that the referee concerns must be fully addressed and their suggestions taken on board. Please address all referee concerns in a complete point-by-point response. Acceptance of the manuscript will depend on a positive outcome of a second round of review. It is EMBO reports policy to allow a single round of major revision only and acceptance or rejection of the manuscript will therefore depend on the completeness of your responses included in the next, final version of the manuscript.

We realize that it is difficult to revise to a specific deadline. In the interest of protecting the conceptual advance provided by the work, we recommend a revision within 3 months (29th Oct 2022). Please discuss the revision progress ahead of this time with the editor if you require more time to complete the revisions.

You can either publish the study as a short report or as a full article. For short reports, the revised manuscript should not exceed 27,000 characters (including spaces but excluding materials & methods and references) and 5 main plus 5 expanded view figures. The results and discussion sections must further be combined, which will help to shorten the manuscript text by eliminating some redundancy that is inevitable when discussing the same experiments twice. For a normal article there are no length limitations, but it should have more than 5 main figures and the results and discussion sections must be separate. In both cases, the entire materials and methods must be included in the main manuscript file.

IMPORTANT NOTE: we perform an initial quality control of all revised manuscripts before re-review. Your manuscript will FAIL this control and the handling will be DELAYED if the following APPLIES:
1) A data availability section providing access to data deposited in public databases is missing. If you have not deposited any data, please add a sentence to the data availability section that explains that.
2) Your manuscript contains statistics and error bars based on n=2. Please use scatter blots in these cases. No statistics should be calculated if n=2.

When submitting your revised manuscript, please carefully review the instructions that follow below. Failure to include requested items will delay the evaluation of your revision.

1) a .docx formatted version of the manuscript text (including legends for main figures, EV figures and tables). Please make sure that the changes are highlighted to be clearly visible.

2) individual production quality figure files as .eps, .tif, .jpg (one file per figure). See https://wol-prod-cdn.literatumonline.com/pb-assets/embo-site/EMBOPress_Figure_Guidelines_061115-1561436025777.pdf for more info on how to prepare your figures.

3) We replaced Supplementary Information with Expanded View (EV) Figures and Tables that are collapsible/expandable online. A maximum of 5 EV Figures can be typeset. EV Figures should be cited as 'Figure EV1, Figure EV2" etc... in the text and their respective legends should be included in the main text after the legends of regular figures.

- For the figures that you do NOT wish to display as Expanded View figures, they should be bundled together with their legends in a single PDF file called *Appendix*, which should start with a short Table of Content. Appendix figures should be referred to in the main text as: "Appendix Figure S1, Appendix Figure S2" etc. See detailed instructions regarding expanded view here:

- Additional Tables/Datasets should be labeled and referred to as Table EV1, Dataset EV1, etc. Legends have to be provided in a separate tab in case of .xls files. Alternatively, the legend can be supplied as a separate text file (README) and zipped together with the Table/Dataset file.

4) a .docx formatted letter INCLUDING the reviewers' reports and your detailed point-by-point responses to their comments. As part of the EMBO Press transparent editorial process, the point-by-point response is part of the Review Process File (RPF), which will be published alongside your paper.

5) a complete author checklist, which you can download from our author guidelines . Please insert information in the checklist that is also reflected in the manuscript. The completed author checklist will also be part of the RPF.

6) Please note that all corresponding authors are required to supply an ORCID ID for their name upon submission of a revised manuscript (). Please find instructions on how to link your ORCID ID to your account in our manuscript tracking system in our Author guidelines

7) Before submitting your revision, primary datasets produced in this study need to be deposited in an appropriate public database (see https://www.embopress.org/page/journal/14693178/authorguide#datadeposition). Please remember to provide a reviewer password if the datasets are not yet public. The accession numbers and database should be listed in a formal "Data Availability" section placed after Materials & Method (see also https://www.embopress.org/page/journal/14693178/authorguide#datadeposition). Please note that the Data Availability Section is restricted to new primary data that are part of this study. * Note - All links should resolve to a page where the data can be accessed. *
If your study has not produced novel datasets, please mention this fact in the Data Availability Section.

8) We would also encourage you to include the source data for figure panels that show essential data. Numerical data should be provided as individual .xls or .csv files (including a tab describing the data). For blots or microscopy, uncropped images should be submitted (using a zip archive if multiple images need to be supplied for one panel). Additional information on source data and instruction on how to label the files are available at .

9) Our journal also encourages inclusion of *data citations in the reference list* to directly cite datasets that were re-used and obtained from public databases. Data citations in the article text are distinct from normal bibliographical citations and should directly link to the database records from which the data can be accessed. In the main text, data citations are formatted as follows: "Data ref: Smith et al, 2001" or "Data ref: NCBI Sequence Read Archive PRJNA342805, 2017". In the Reference list, data citations must be labeled with "[DATASET]". A data reference must provide the database name, accession number/identifiers and a resolvable link to the landing page from which the data can be accessed at the end of the reference. Further instructions are available at https://www.embopress.org/page/journal/14693178/authorguide#referencesformat

10) Regarding data quantification (see Figure Legends: https://www.embopress.org/page/journal/14693178/authorguide#figureformat)

The following points must be specified in each figure legend:

- the name of the statistical test used to generate error bars and P values,

- the number (n) of independent experiments (please specify technical or biological replicates) underlying each data point,

- the nature of the bars and error bars (s.d., s.e.m.),

- If the data are obtained from n {less than or equal to} 2, use scatter blots showing the individual data points.

Discussion of statistical methodology can be reported in the materials and methods section, but figure legends should contain a basic description of n, P and the test applied.

- Please also include scale bars in all microscopy images.

We would also welcome the submission of cover suggestions, or motifs to be used by our Graphics Illustrator in designing a cover.

As part of the EMBO publication's Transparent Editorial Process, EMBO reports publishes online a Review Process File (RPF) to accompany accepted manuscripts. This File will be published in conjunction with your paper and will include the referee reports, your point-by-point response and all pertinent correspondence relating to the manuscript.

You are able to opt out of this by letting the editorial office know (emboreports@embo.org). If you do opt out, the Review Process File link will point to the following statement: "No Review Process File is available with this article, as the authors have chosen not to make the review process public in this case."

I look forward to seeing a revised form of your manuscript when it is ready. Please use this link to submit your revision: https://embor.msubmit.net/cgi-bin/main.plex

Yours sincerely,

Esther Schnapp, PhD
Senior Editor
EMBO reports

Full Revision

Manuscript number: RC-2 22-
Corresponding author(s): Louise Cheng

[Please use this template only if the submitted manuscript should be considered by the affiliate journal as a full revision in response to the points raised by the reviewers.

If you wish to submit a preliminary revision with a revision plan, please use our "Revision Plan" template. ***It is important to use the appropriate template to clearly inform the editors of your intentions.****]*

. eneral Statements optional

We have added the following significant changes to improve the manuscript.

- We have performed new nano-Dam expression profile experiment to identify Dpn target genes specifically in the medulla. We have found that Dpn preferentially bind to mid-tT s.
- We have performed additional experiments to show that dedifferentiation mediated by the loss of Nerfin-   and Lola also exhibit stalled progression of the temporal series.

2. oint-by-point description of the revisions

Reviewer 1

Reviewer   (Evidence, reproducibility and clarity (Re uired)):

Summary
Authors show that overexpression of bHLH transcription factor Dpn in the medullary neurons of the Drosophila optic lobe results in the dedifferentiation of these neurons back into the NBs. These dedifferentiated NBs ac uire and maintain mid-temporal identity, express Ey and Slp, and show delayed onset of tT   Tailless (Tll), leading to an excess of neurons of mid-temporal fate at the expense of late temporal fate neurons and glial cells. The dedifferentiated NBs are stalled in the cell cycle and fail to undergo terminal differentiation. Over expression of tT Dicheate (D) or promoting   S transition pushed these NBs to late stages of the temporal series, partly rescuing the neuronal diversity and causing their terminal differentiation. They also show that the dedifferentiation of NBs by Notch hyper-activation also exhibited stalled temporal progression, which is restored by D overexpression.
Authors suggest that cell cycle regulation and tT   are primary to the proliferation and termination profile of dedifferentiated NBs.
Using these conclusions, the authors emphasize the need to recreate the right temporal profile and ensure appropriate cell cycle progression to use dedifferentiated NSC for regenerative purposes or prevent tumorigenesis originating from differentiated cell types.

Major comments:
- Are the key conclusions convincing?
Most conclusions are convincing however, some issues are pointed out below.

- Should the authors ualify some of their claims as preliminary or speculative, or remove them altogether?

The authors have overexpressed Dpn and shown that medulla neurons dedifferentiate to NBs, similar to the loss of function phenotype seen for the Nerfin- of which Dpn is a target. They also show that temporal series progression defect is also seen in the case of dedifferentiated NB generated by Notch over-activation.
Using these two examples, the authors suggest that for dedifferentiated NSC, which are to be used for the regenerative purpose, one needs to recreate the right temporal profile and ensure cell cycle progression occurs appropriately. Authors also claim that to prevent tumorigenesis originating from differentiated cell types, one needs to recreate the right temporal profile and ensure cell cycle progression occurs appropriately.

While agree with this, think this is an overreaching conclusion based on just these two examples. f they could show the same for one more method of dedifferentiation (or, e.g. Lola) happening in medulla neurons which happens by a mechanism independent of Nerfin- , Dpn, Notch axis, the argument will become more convincing and broad.
We have characterized the temporal identity and progeny of Lola-Ri induced ectopic neuroblasts (NBs) in igure and Nerfin- in igure E . We found that ectopic NBs generated via Lola-Ri express Slp at the expense of Tll. We further show that overexpression of D in Lola-Ri induced ectopic NBs, promote the progression of the temporal series, resulting in reduced proportion of Slp NBs and increased proportion of Tll NBs. or Nerfin- mutant clones, we found the ectopic NBs were stalled in the Tll temporal window, and the onset of Repo expression was delayed.

Also when authors mention N mediated dedifferentiation, they need to inform that Dpn is a direct target of Notch in NBs (Doi. . j.ydbio.2 . .), they do so in the discussion, but mentioning it here gives a broader context to the reader.
We have included that Dpn is a target of Notch when first mentioned.

Another important point that needs a mentioned here is that conclusions are based on dedifferentiation happening in the medulla neurons, which are considered less stable since they lack rospero. Therefore whether this conclusion can be generalized for all the tumors arising from dedifferentiation in the CNS (eg, those arising from N CD activation in the central brain or thoracic region of the NC) is another concern. Maybe authors can consider making a more conservative claim.

eneralizing this conclusion to rospero expressing NBs lies outside the scope of the current study and cannot be addressed here because central brain Type- NBs use a different set of tT s.
n this revision, we have modified our claim and clarified that all of our conclusion are medulla neuron-specific.

- Would additional experiments be essential to support the claims of the paper? Re uest additional experiments only where necessary for the paper as it is, and do not ask authors to open new lines of experimentation.
Experiments with Lola knockdown mutants in medulla neurons can be done uickly, in my opinion, and will substantiate this claim.
Another obvious uestion that comes to mind is if medulla neurons dedifferentiate on overexpression of Dpn, does the same happen in nerfin- mutant clones as well? And if yes, why has the author not done similar experiments for nerfin- mutants.
We have assessed the temporal identity of ectopic NBs produced by nerfin- mutant clones (igure E). We found that the ectopic NBs are stalled in the Tll temporal window, and the expression of Repo is delayed.

lease show Ey staining in ig-2 if possible, it will also help to add a line on why Slp was used as marker for mid tT s instead of Ey.
Ey has been tuned up, and is now in ig 2 D-D and - . Slp was used as a marker of mid tT s as Ey is expressed also in neurons thus would also be present in deep sections of control clones, whereas Slp is not expressed in neurons. We therefore used Slp as a proxy for mid-temporal identity throughout our study. We have included this explanation in the text.

n Model shown in last figure Dpn is shown to repress D and activate Slp. Can authors show that Dpn overexpression represses D and activate Slp either by antibody staining or by RT CR.
n igure E C-D , we show in clones that overexpress Dpn, that high levels of Slp (open arrows) corresponds with low levels of D. eedback inhibition exists, so that high levels of D (closed arrows) also correspond with low levels of Slp.
We have shown that Dpn overexpression activates Slp: igure 2 and shows that Dpn overexpression causes Slp to be upregulated.

- Are the suggested experiments realistic in terms of time and resources? t would help if you could add an estimated cost and time investment for substantial experiments.
Experiments with Lola and nerfin- mutants can be done in a few months. cannot comment on the cost involved.
- Are the data and the methods presented in such a way that they can be reproduced?
es

- Are the experiments ade uately replicated and statistical analysis ade uate?
Replication and statistical analysis are fine.

The activated Notch experiments show only three data points in all the experiments. t will be good to increase this number.
We have added additional data points in igure .

Minor comments:
- Specific experimental issues that are easily addressable.
There is a problem with ig- (both E and have EdU in clone Mira in the clone as y-axis), do not understand how the ig- let them conclude that D overexpression increases the rate of neuronal production.
We think the reviewer is mistaken here. n the text we said: We found that D overexpression <u>did not</u> significantly increase neuronal production, suggesting that it is likely that cell cycle progression lies upstream or in parallel to the temporal series, to promote the generation of neurons.

n one place, the authors conclude, Together, this data suggests that it is likely that cell cycle progression lies upstream of the temporal series, to promote the generation of neurons . Authors should consider adding medulla NBs at the end of the sentence since cell cycle progression being upstream of temporal series is already known in Type- NBs, as pointed out by authors as well (Ameele and Brand 2).
We have added in the OL, to this sentence.

n the discussion authors says that Our data support the possible links between cell cycle progression and the expression of temporal regulators controlling NB proliferation and cellular diversity . This is new information, as the 2 study did not show how cell diversity changes with a changed tT profile. think the authors should elaborate on this point to highlight how this is different from what is already known from the 2 study (done in the context of Type- NBs). Maybe they need to highlight that the cell cycle directs regulates the progression of temporal series compared to the earlier observation where temporal series was shown to be downstream of the cell cycle.
We have expanded the link between cell cycle tT s in our revised discussion.

n fig- J in clones even after 2 AHS, Dpn continues to be overexpressed but these cells undergo terminal differentiation, can authors comment why is it so?
The ig J is now igure J. While Dpn is overexpressed, it progresses through the temporal series at a slower pace due to a delay in cell cycle progression, as well as delayed onset of D, However, these NBs can eventually reach terminal temporal identity, and are thus able to undergo terminal differentiation. We have now included an additional piece of data that shows NBs induced by Dpn overexpression do eventually turn on Tll (igure E 2 A-).

n one place authors say, To better assess the cumulative effect of the neurons made throughout development, EyO - AL was used to drive the expression of Dpn maybe some background on why use this specific AL .
Also a line about why MR H - AL eyO - AL and and eyR - AL were used.

We have included in igure E the expression analysis of MR H - AL , eyO - AL and eyR - AL . We have also added a section regarding why these al s were used in the methods section.
.

- Are prior studies referenced appropriately?
es, but in a few places, some references can be added.
An important point that needs to be mentioned for the context is the medulla neurons do not use rospero for terminal differentiation and are thus considered less stable (DO : . 2 2 dev.).
We beg to disagree with the reviewer in terms of ros is not re uired for terminal differentiation of medulla neuroblasts. Li et al., 2 shows that nuclear ros is found in the oldest NBs prior to their terminal differentiation. We do agree that differentiated state of medulla neurons is less stable, possibly owing to absence of ros, and we have included this in our discussion.

n discussion, the authors say that t would be interesting to explore whether N similarly acts on these target genes to specify cell fate and proliferation profiles of dedifferentiated NBs. There is a study looking at Notch targets in NB hyperplasia (DO : . 2 2 dev. 2 2) whether that study shows if any of the cell cycle genes are downstream of activated Notch, needs a mention here.
We thank the reviewer for this suggestion. None of the cell-cycle genes bound by Dpn were N targets in hyperplasia.

Also, when authors mention N mediated dedifferentiation, they need to inform that Dpn is a direct target of Notch in NBs (Doi. . j.ydbio.2 . .). They do so in the discussion, but mentioning it in the introduction or results will give a broader context to the reader.
We have now mentioned that Dpn is a target of Notch in the intro and results section.

Another gene that needs a mention is Brat , which regulates both Dpn and Notch, and causes dedifferentiation and tumors in CNS, think this gene and its interaction with Dpn and Nerfin and Notch needs to be discussed either in the introduction or discussion.
We have added a sentence on Brat in the discussion.

- Are the text and figures clear and accurate?
The main figures are not labeled. Therefore, it was very annoying to deduce the specific figure numbers.
This is a uirk of the way figures were uploaded in review commons.

There are or 2 places where figure calling is wrong in the text.
We have check and correct all call-outs.

The mage ig- shows cycD and CD at the 2-M transition while the text says it supports S, which is indeed the case, the figure needs modification.

Full Revision

We have modified the cell cycle figure and cdk cycD to g s.

- Do you have suggestions that would help the authors improve the presentation of their data and conclusions?
The presentation is okay, in my opinion.

Reviewer (Significance (Re uired)):

- Describe the nature and significance of the advance (e.g. conceptual, technical, clinical) for the field.

The factors leading to dedifferentiation of the neurons have been identified previously by groups of Chris Doe (mldc, DO : . 2 2 dev.), Andrea brand (. j.devcel.2 . . .) as well as the authors of this paper (. gad.2 2 2. , . j.celrep.2 . . .). However, many uestions remained unaddressed regarding such NB generated from neuronal dedifferentiation. or example, whether these cells contribute to native cell diversity of the CNS, undergo timely differentiation or their progeny cells incorporated into appropriate circuits is not well understood. Successful execution of these phenomena is critical for generating functional CNS and such insights are crucial for understanding the origin of tumorigenesis in CNS or employing dedifferentiated NSC for regenerative purposes.

This study is an overexpression-based study, however, some of the results give significant conceptual insights into the tumors arising out of the dedifferentiation of the neurons. t also gives insights into the fact that the dedifferentiated cells need to be carefully examined for the temporal factor profile before they can be employed for regeneration or any therapy targeting them.
However, in my opinion, they need to test this idea at least in one more system of neuronal dedifferentiation, preferably independent of the nerfin- Notch Dpn axis to generalize this claim.

- lace the work in the context of the existing literature (provide references, where appropriate).
Cerdic Maurange s group had looked at the role of temporal factors and identified the early phase of malignant susceptibility in Drosophila in 2 (doi: . eLife.). Andrea Brand s group has shown in a 2 paper that cell cycle progression is essential for temporal transition in NBs (doi: . eLife.). Both these studies were in the context of Type- NBs, which express rospero, which is crucial for the differentiation of the neurons.
reviously the authors have studied type- NBs and shown by Targeted Dam D that Dpn is Nerfin- target. They also show that Nerfin- mutants show dedifferentiation of neurons. They follow up on this observation in medulla neurons, where they find that Dpn overexpression results in their dedifferentiation into medulla NBs. Medulla NBs differ from Type- NBs in using a separate set of tT s. Also, Type- NB and neurons arising from them use rospero for terminal differentiation, while medulla neurons do not express rospero and are therefore considered less stable (DO : . 2 2 dev.).

The importance of the study lies in the results that show that the NB arising out of dedifferentiation of medulla neurons takes up mid-temporal fate. These NBs are stalled in Slp expressing mid-temporal stage unless the cell cycle is promoted by overexpression of cell cycle genes regulating S transition.
Authors also show that overexpression of D promotes the progression of temporal series in these dedifferentiated NBs, which could partly rescue neuronal diversity and result in terminal differentiation. Thus D plays an important role in determining the type of neurons these NBs generated. This suggests that knowing the tT profile of these types of dedifferentiated NBs is vital if these cells were to be used for regenerative purposes. Authors further claimed that cell cycle regulation and tT s are critical determinants of the proliferation and termination profile of dedifferentiated NBs.

- State what audience might be interested in and influenced by the reported findings.
The study will be of broader interest to researchers interested in central nervous system patterning, regeneration, and cancer biology.

- Define your field of expertise with a few keywords to help the authors contextualize your point of view. ndicate if there are any parts of the paper that you do not have sufficient expertise to evaluate.
Drosophila, central nervous system patterning and cell fate determination of neural stem cells.

Reviewer 2 (Evidence, reproducibility and clarity (Re uired)):

Stem cells can divide asymmetrically to self-renew the stem cell while generating differentiating sibling cells. To restrict the number and type of differentiating sibling cells, stem cells often undergo terminal differentiation. Terminally differentiated cells can dedifferentiate and revert to a stem cell like fate. However, the underlying molecular mechanisms are incompletely understood in vivo.
Here, een et al., use Drosophila neural stem cells (called neuroblasts) to investigate how terminal differentiation is regulated. Neuroblasts faithfully produce the correct number and type of neuronal cells through temporal patterning and regulated terminal differentiation. The authors show that misexpression of the bHLH transcription factor Deadpan (Dpn) induces ectopic neuroblasts, which predominantly express mid-temporal transcription factors at the expense of late-temporal transcription factors. As a conse uence, these ectopic neuroblasts also fail to produce Repo positive glial cells and are stalled in their cell cycle progression. The authors provide evidence that promoting cell cycle progression and overexpression of the transcription factor Dichaete (D) is sufficient to restore the temporal transcription factor series, neuronal diversity and timely neuroblast differentiation.

This is an interesting study that will be of interest to the stem cell field. However, encourage the authors to consider the following criti ues:

. Explain the rationale for the three different neuronal NB drivers (MR H - AL , eyO - AL , eyR - AL . How are they expressed?
We have included in igure E the expression analysis of MR H - AL , eyO - AL and eyR - AL . We have also added a section regarding why these al s were used in the methods section.

2. The rationale for the Edu experiment (igure S) is not clear. Why is this a measure for the production of neuronal progeny? or the correct interpretation of these results, the authors should also provide control clones or Edu experiments of regular neuroblasts.
We intended to show that ectopic NBs produce differentiated neurons using the EdU assay. We have repeated this experiment and the conclusions was still not clear. As we show that dedifferentiated NBs make Toy progeny, we have therefore withdrawn the EdU data from the original S out of the ms.

. How was of Mira (igure and below) or the of tT s (igure 2H onward) uantified?
We have added more detailed explanation to how Mira and T uantified to the methods section.

or instance, igure 2C- often shows clonal signal that is not highlighted with the dashed lines and the corresponding tT intensity does not match the intensity in the outlined clone (eg. igure 2D-D a large optic lobe clone is negative for Ey. igure 2E-E an unmarked clone is negative for Slp).
This is now igure 2 and 2J. n both of these cases, the clones that were not outlined (negative for Ey and Slp) are in the C and not the O C, therefore were not considered in our analysis.

Similarly, the Hth signal is very weak to begin with so it is unclear how this was uantified. How was determined what constitutes real signal vs. background noise?
Additional explanations in the methods section is needed to assess the robustness of the data.
We agree that Hth signal is very weak. However, there is a signal outside of the clone in igure 2H. We have withdrawn the data that was originally in igure 2 -L. These clones were very small, Hth staining was poor (despite our several attempts to boost the signal noise ratio).
We have expanded the methods section to explain how these uantifications were done.

. This sentence should be rephrased: As the tumour cell-of-origin can define the competence of tumour NBs to undergo malignancy (arnsworth et al., 2 Narbonne-Reveau et al., 2), we next tested whether the temporal identity of the dedifferentiated NBs were conferred by the age of the neurons they were derived from.
The connection between tumorigenicity and temporal identity is not really clear and should be briefly reintroduced for this paragraph.
We have deleted this sentence.

. igure 2 -N: The experimental outline in and J should be grouped with the corresponding images to clarify what is compared. Also, there are no images for the control clones, which make a comparison difficult. The images are also too small. cannot really see the Hth, or Slp signal in the small clones shown in igure 2 -L .

We have split igure 2 into two igures. We have included the control clones that are wildtype clones where the T s are expressed in the superficial NBs.

We have enlarged the images.

We agree that Hth staining is hard to see in the small clones. We have withdrawn this data.

We have shown Slp staining at various time points after heatshock, and this is included as igure . We have magnified the small clone in igure C-C in C , so that Slp staining in small clones can be better visualised.

. igure H: t is not clear why there are only a small group of Nbs that are positive for Mira. lease explain.

This is now igure H. Most NBs have terminated by this time point, we have explained this better within the text.

. igure -M: lease explain how the Toy signal was measured and uantified.

We have explained Toy volume uantification in the methods section.

. The TaDa data set is very interesting but the following might be an overstatement: We found that Dpn directly binds to slp as well as the Sox-family T dichaete (D) which is expressed in medulla NBs after slp (Li et al., 2) (igure S A-B).

More direct binding assays might be needed to show that Dpn directly binds to slp and D. f this is already shown, clarify the sentence to indicate what is published and what is extracted from the data shown here.

The Dam D (both Targeted Dam D and nano-Dam) data show that Dpn binds (i.e. there is a statistically significant peak at DR .) directly at these loci (igure S and new igure).

We have clarified the sentence to indicate this in our revision.

Also, what is the rationale for this statement: Consistent with the model that D represses Slp- ... ?

We have expanded on this in the text and added a reference.

This might be an overinterpretation: D overexpression in UAS-Dpn NBs promoted their pre-mature cell cycle exit at hrs A using eyR - AL . The data shows loss of Mira signal, which could occur through different mechanisms.

Our data shows that the NBs that express D express high levels of Tll, the terminal temporal transcription factor (igure). n addition, we show that there is an increase in Tll and Repo progeny (igure -H , J-). Together, this suggests that D overexpression promotes the progression of the temporal series and the precocious transition from NB to glial cell fate. However, it is possible that Mira cells can disappear via cell death. We have assessed this

possibility by staining for cell death marker Dcp at hr A (igure E E-), where we found that there was in fact less cell death. Therefore, it is unlikely that Mira NBs disappeared via cell death.

Reviewer 2 (Significance (Re uired)):

These appear to be novel and significant findings that will enhance our understanding of the temporal progression and terminal differentiation program of neural stem cells in vivo.
think the findings will be of interest to cell, developmental cell and stem cell biologists.

My primary expertise is in the cell biology of fly neural stem cells and asymmetric cell division of neuroblasts. Although am not intimately familiar with the differentiation and differentiation literature, consider the findings reported here relevant and impactful.

Reviewer (Evidence, reproducibility and clarity (Re uired)):

The discoveries that the author describe in this manuscript are very specific to dedifferentiated neuroblasts created by UAS-dpn transgene overexpression. Dpn is endogenously expressed in optic lobe neuroblast throughout larval stage, which makes understanding how Dpn regulates gene expression based on the authors results (suppression of cell-cycle genes, and promotion of a specific temporal state) confusing.

Our data relate specifically to gene regulation by Dpn in a dedifferentiated context, and do not seek to understand Dpn regulation in wt neuroblasts. We are not trying to claim that we know what Dpn is doing in WT NBs.

Therefore, this manuscript does not advance our understanding of regulation of temporal identity and cell cycle progression in optic lobe neuroblasts during normal neurogenesis.
The author s state:
However, beyond the fact that misexpression of these factors and pathways caused the formation of ectopic NBs, whether these dedifferentiated NBs faithfully produce the correct number and types of neurons or glial cells, or undergo timely terminal differentiation, has not been assessed. These characteristics are key determinants of overall CNS size and function, thus are important parameters when considering whether dedifferentiation leads to tumourigenesis or can be appropriately utilized for regenerative purposes.
at the end of introduction. f this is a true primary goal of this study, the authors should describe it in abstract. Otherwise, readers will lose enthusiasm to read this manuscript in abstract and no longer read the following sections.
We have discussed this concept extensively in the discussion, and have mentioned this idea in the abstract.

Results

. The authors should describe the expression pattern of all three of the al drivers used. While there are dotted outlines in the supplemental figure, there should be a description in the main text for the expression pattern of these lines which described with temporal state of NBs these lines are expressed in, and whether they are also expressed in the neurons or not.

We have included in igure E the expression analysis of MR H - AL , eyO - AL and eyR - AL . We have added in the text a section regarding why these al s were used. We have used -trace to characterize the real time and lineage specific expression of these drivers. We have also characterized whether these drivers are expressed in the NBs, and whether they continue to drive the expression of transgenes during pupal development. More importantly, we have shown that these drivers are expressed in the neurons, and that overexpression of Dpn causes dedifferentiation of neurons using all three drivers.

2. The authors claim that overexpression of Dpn in the medulla region causes dedifferentiation. The data provided however is not sufficient to conclude that dedifferentiation is occurring. The AL s used all drive in the NBs, and so it is unclear if the ectopic NBs ever became mature neurons. n addition, the lack of ectopic NBs in the clonal analysis hrs AHS does not prove that ectopic NBs at 2 hrs AHS must have come from mature neurons. To demonstrate dedifferentiation, the authors should use a driver system that is specific to mature neurons, and then overexpress dpn and look for mira cells. Currently, the authors data does not prove that mature neurons dedifferentiatiate into ectopic NBs upon Dpn OE.

n igure E , we have used lineage tracing (-Trace) to demonstrate that the MR H - AL is exclusively expressed within the medulla neurons (real time) and was expressed in some MCs in the past. We have now utilised MR H - AL with a al ts (igure E A-D), to restrict the timing of Dpn induction to rd instar (degrees for days, 2 degrees for 2 days), so that the driver expression is restricted to neurons. Similar strategies to induce dedifferentiation was utilized in DO : . 2 2 dev. and DO : . j.devcel.2 . . , and has similarly concluded that mature neurons dedifferentiate into ectopic NBs upon Nerfin- and Lola knockdown.

. What is the conclusion of fig 2C-H?

ig 2C-H assessed the expression of tT s in UAS-dpn induced ectopic NBs, and found that Slp is expressed in these NBs.

. As the tumor cell-of-origin can define the competence of tumor NBs to undergo malignancy identity of the dedifferentiated NBs were conferred by the age of the neurons they were derived from . This sentence is confusing. What are the authors investigating in the following experiment? Do they want to see ectopic NBs keep their early identity like Chinmo in ventral cord tumor NB? Or tll-positive NB s progenies can dedifferentiate to ectopic NB, but this ectopic neuroblast is not able to keep proliferation in pupal stage? t is hard to understand the connection of this sentence and the following experiment.

We have deleted this sentence, and have clarified why we are doing the experiments.

. The Dam D experiment described used wor-gal as a driver, which means the Dpn binding profile generated is coming from not only optic lobe NBs, but central brain NBs and NC NBs as well. n Magadi et al. (2 2), the authors profiled Dpn binding in CNS hyperplasia, and found that dpn strongly bound Nerfin- and gcm. However, it does not bind cell cycle genes in this context. How do the authors know that the region that they claim are bound by dpn are bound in medulla NBs?
We agree with the reviewer that the profile is not specific to medulla NBs. To assess Dpn binding profiles specifically in the medulla NBs, we have now used the NanoDam techni ue (https: doi.org . j.devcel.2 22. .) for profiling -fusion protein of Dpn (recombineered locus under endogenous control), with a medulla specific driver (eyR - AL), this data is in igure , igure E and E .

The authors should also include tracks to show dpn binding at Nerfin- , as well as the other tT s (hth, ey, tll, and gcm). roviding this data will help to understand if Dpn binding is specific to the mid-temporal genes, as Dpn expression is known to be expressed in all medulla NBs regardless of temporal state.
We have included the tracks of the other transcription factors (igure and igure Ev).

. Currently, the Dam D data does not help to interpret the Dpn overexpression phenotype at all. nside of flip-out clone, some cells show Slp- expression while others showed D expression. The authors explain that Slp- and D suppress their expression to each other. But the Dam D data indicate that both Slp- and D are Dpn target genes. f this is true, why did they observe the mosaic expression pattern inside of the same clone.
We observed that high levels of Slp- is correlated with low levels of D. This suggest to us that the initial stochastic differences accounts for where Slp- is high is where D is low, and vice versa.

. The authors hypothesized if Dpn activated Slp- directly. Does this mean that Dpn directly activate transcription of Slp- ? t is well known that Dpn is transcriptional repressor. Hes family proteins form a homodimer or heterodimer with another Hes protein and interacts ro, which recruits a Histon deacetylase protein. The author s claim does not fit to the model what we currently believe. n addition, the authors claimed that Dpn inhibits cell cycle gene transcription directly. This is inconsistent to their claim that Dpn directly activate Slp- expression. f the authors want to claim that Dpn has two different functions in this context, the authors must demonstrate it by experimental results.
We have discussed these models in the Discussion, and make our claims more conservative, as we do not have direct experimental evidence to prove or disprove the model that Dpn is acting as an activator in this context. However, we note that the model proposed by the reviewer that Dpn is a transcriptional repressor that interacts with ro and recruits HDAC Rpd is not based on genome-wide binding data but on studies of specific loci. There is no genome-wide evidence that we are aware of that Dpn always interacts with ro in all

contexts and the downstream recruitment of HDAC and transcriptional repression by ro has been uestioned in the literature following Ch -se studies (aul et al., LoS enet, 2). urther, a study that profiled the genome-wide binding of a related Hes protein, Hairy, and ro, via Dam D found very little overlap between the two proteins (Bianchi- ries et al, LoS Biol, 2), suggesting that Hes proteins do not recruit ro to all, or even most, bound loci. inally, the only other study to look at genome-wide binding of Dpn (Magadi et al., 2 2) found that around half of the direct targets of Dpn in NB hyperplasia identified in that study through Ch -se were upregulated, not repressed, following Dpn overexpression prima facie evidence that Dpn can play both an activating and repressing role in NBs. This is also the most parsimonius explanation of our own findings in this study, as we observe Dpn binding at active T genes in medulla NBs through NanoDam.

. Related to the above uestion, wondered if the authors guess Dpn activate or repress D transcription by binding to D promoter region because they claimed that Dpn activate Slp- , while suppress cell cycle genes.
We have discussed the regulation of the tT genes by Dpn further in the Discussion.

. am confused to the claim that Dpn suppress cell cycle genes expression. Dpn overexpression induces dedifferentiation of neuron into NB and re-entry into the cell cycle. f Dpn suppress cell cycle genes how can the dedifferentiated cell re-enter into the cell cycle?
The data points towards that Dpn overexpression has two separate roles in regulating the cell cycle. Of course dedifferentiation re uires a commitment of neurons into the cell cycle (this we think is still happening), however, we think once these cells have turned on NB markers, they have limited ability to progress through the cell cycle. We have discussed this point in the Discussion.

. igure looked redundant because we know Dpn is a direct target of Notch. t is obvious that an upstream factor overexpression can induce the identical phenotype to the phenotype induced by overexpression of a downstream factor.
A direct target does not necessarily infer the same phenotype. To assess whether the mechanisms apply to other dedifferentiation models, we have added Lola-RNAi and Nerfin- data to our revised manuscript.

Minor comments:
. Typo in main text: MR H - AL should be MR H - AL
We have fixed this.

2. n figure E-H the dotted line regions indicated the clones are not shown in the merge image. lease include
We have fixed this.

. Typo in discussion paragraph 2: temporal series was no sufficient to rescue cycle cycle progression

We have fixed this.

Reviewer (Significance (Re uired)):

nsights into the developmental capacity of dedifferentiated stem cells will likely lead to novel strategy to replenish cells lost due to aging, injury and diseases in regenerative medicine.

1st Revision - Editorial Decision 2nd Mar 2023

Dear Dr. Cheng,

Thank you for the submission of your revised manuscript. We have now received the enclosed reports from the referees. All referees still have a few more minor suggestions that I would like you to address and incorporate before we can proceed with the official acceptance of your manuscript.

Referee 2 is not correct in that a ms cannot have both EV figures and an Appendix. Both is possible. If you prefer, you could move all EV figures into an Appendix file, but this is not a must.

Some editorial requests also need to be addressed:

- Please add up to 5 keywords to the ms file.

- Please update the conflict of interest subheading to "Disclosure and Competing Interest Statement".

- The author credits should be removed from the ms. We now use CRediT to specify the contributions of each author in the journal submission system. CRediT replaces the author contribution section. Please use the free text box to provide more detailed descriptions, if you wish. See also guide to authors https://www.embopress.org/page/journal/14693178/authorguide#authorshipguidelines.

- In the REFERENCE list, Li et al (Nature) has no issue/page #. Please add.

- The FUNDING INFO in our online submission system and the ms don't match, the ARC grant #s are not in the ms, please add.

- FIGURE CALLOUTS: The panels are not alphabetically called out, please correct.
Figs 4A-C, 6C-F, I-K, 7D-F, J-K, 8H, 9M, EV1I-L, O-P, EV2F-G, EV3A-B, EV4A-C,F, callouts are missing. Fig EV5M is called out, but there is no 'M' panel.

- IF you keep the Appendix file, a table of content must be added that includes page numbers.

- The sub-headings 'Figure Legends' and 'Expanded View Figure Legends' need to be added to the ms file.

- I attach to this email a related ms file with comments by our data editors. Please address all comments in the final ms.

I would like to suggest some minor changes to the abstract that needs to be written in present tense. The meaning of the first sentence in the abstract is not clear, please re-write or delete. The ms title is not correct, I think. How does the cell cycle regulate dedifferentiation-derived NSCs? It would be good to think about a new title along the lines that dedifferentiation-derived NSCs behave differently from normal NSC.

Please let me know whether you agree with the following abstract:

Dedifferentiation is the reversion of mature cells to a stem cell-like fate, whereby gene expression programs are altered and genes associated with multipotency are (re)expressed. Misexpression of multipotency factors and pathways causes the formation of ectopic NSCs. Whether dedifferentiated NSCs faithfully produce the correct number and types of neurons and glial cells, or undergo timely terminal differentiation, has not been assessed. Here we show that ectopic NSCs induced via bHLH transcription factor Deadpan (Dpn) expression in the optic lobes of the developing Drosophila CNS fail to undergo appropriate temporal progression. Ectopic NSCs express mid-tTF Sloppy-paired 1 (Slp-1) at the expense of late-tTF Tailless (Tll); consequently generating an excess of Twin of eyeless (Toy) positive neurons and fewer Reversed polarity (Repo) positive glial cells. Dpn overexpression also stalls the progression through the cell cycle, and impairs timely terminal differentiation.

EMBO press papers are accompanied online by A) a short (1-2 sentences) summary of the findings and their significance, B) 2-3 bullet points highlighting key results and C) a synopsis image that is exactly 550 pixels wide and 200-600 pixels high (the height is variable). You can either show a model or key data in the synopsis image. Please note that text needs to be readable at the final size. Please send us this information along with the final manuscript.

I look forward to seeing a final version of your manuscript as soon as possible. Please use this link to submit your revision: https://embor.msubmit.net/cgi-bin/main.plex

Best regards,
Esther

Esther Schnapp, PhD
Senior Editor
EMBO reports

Referee #1:

The reviewer appreciates the authors adequately addressed the suggestions, and would like to suggest one minor revision prior to publication of this study.

line L350-361 on page 11, the authors should acknowledge and incorporate findings from studies below in this discussion paragraph
1. doi: 10.7554/eLife.13463
2. doi: 10.7554/eLife.50375
3. doi: 10.1242/dmm.044883
4. doi: 10.1126/sciadv.abi4529

Referee #2:

Stem cells can divide asymmetrically to self-renew the stem cell while generating differentiating sibling cells. To restrict the number and type of differentiating sibling cells, stem cells often undergo terminal differentiation. Terminally differentiated cells can dedifferentiate and revert to a stem cell like fate. However, the underlying molecular mechanisms are incompletely understood in vivo.
Here, Veen et al., use Drosophila neural stem cells (called neuroblasts) to investigate how terminal differentiation is regulated. Neuroblasts faithfully produce the correct number and type of neuronal cells through temporal patterning and regulated terminal differentiation. The authors show that misexpression of the bHLH transcription factor Deadpan (Dpn) induces ectopic neuroblasts, which predominantly express mid-temporal transcription factors at the expense of late-temporal transcription factors. As a consequence, these ectopic neuroblasts also fail to produce Repo positive glial cells and are stalled in their cell cycle progression. The authors provide evidence that promoting cell cycle progression and overexpression of the transcription factor Dichaete (D) is sufficient to restore the temporal transcription factor series, neuronal diversity and timely neuroblast differentiation.

The authors addressed my previous critiques to my satisfaction. Also, I would like to commend the authors for the the amount of data they have presented. This is clearly a lot of work and I do not wish for the authors to spend more time on time consuming experiments. Please consider the following critiques and modify with text changes.

(1) In Figure 2, the authors claim that "...the ectopic NBs in UAS-dpn clones mostly expressed the mid-tTFs Ey, Slp, and D (Figure 2 I-K', M)...)". However, D expression does not appear to be significant. Please change the wording accordingly.

(2) I find it difficult to see how the expression levels of Tll in Figure 9H"-J" is altered given the quality of the stainings. The authors might consider removing that data, or provide higher quality images.

(3) The authors claim that "...nerfin-1 mutant clones exhibited normal progression through the Hth→ Ey→ Slp→ D temporal transcription factor widows (Figure EV5 A-G', L)".
I assume the authors refer to panel J (not L), which shows a significant reduction in Slp expression in nerfin mutant clones. Please change the wording accordingly.

(4) The manuscript might benefit from a more logical figure labeling structure: supplemental data should either be all presented in the 'Appendix' or as EVs but not both. Two different formats for supplemental figures makes little sense in my opinion.

Referee #3:

All the concerns raised earlier by us have been addressed satisfactorily. Even though it is an overexpression study with its limitation and merits (which has been pointed out by other reviewers as well), it conveys an important message (detailed at the end). Therefore we favor accepting this work with minor revisions given below.

In Fig.2. levels of Hth in control and UAS-Dpn are compared and shown to be significantly different. But the representative image used does not do justice. The levels of Hth in control itself are very low.

If possible, replace with a different image and also use of an arrow to show the NB in the superficial plane. Also, authors can show separate channel for Mira as well.

Authors may consider indicating in all the figures the panels which show deep and superficial sections, respectively. This is shown in some figures but not all.

In fig. 9M shows that Dpn is regulating Ey and Slp. Tada/nanoDam data show binding of Dpn on D as well. Any specific reason that is not indicated in the figure.

We could not find FigS4 and S6 in the Supplemental information of this version of the manuscript.

In lines 335-337 and 339-340 of the discussion, it will be a good idea to refer to specific figures establishing these facts.

Suzuki et al., 2013; this reference appears twice in the list.

In fig. 5, when showing the Dpn binding on Dicheate, it's written as Dn instead of D.

FigEV3 C-D, the arrowhead calling is mixed up.

Pg-9 line 271 (Fig6L should be 7L).

Importance :
This study is overexpression-based. However, some of the results give significant conceptual insights into the tumors arising out of the dedifferentiation of the neurons. It also offers insights into the fact that the dedifferentiated cells need to be carefully examined for the
temporal factor profile before they can be employed for regeneration or any other therapy.
It emphasizes the need to recreate the right temporal profile for regenerative purposes and ensure cell cycle progression occurs appropriately. These insights are essential to prevent any tumorigenesis that may arise from using dedifferentiated cell types for regenerative purposes.

The authors addressed the remaining minor issues.

2nd Revision - Editorial Decision **20th Mar 2023**

Dr. Louise Cheng
Peter MacCallum Cancer Centre
305 Grattan St
Parkville, Victoria 3000
Australia

Dear Dr. Cheng,

I am very pleased to accept your manuscript for publication in the next available issue of EMBO reports. Thank you for your contribution to our journal.

At the end of this email I include important information about how to proceed. Please ensure that you take the time to read the information and complete and return the necessary forms to allow us to publish your manuscript as quickly as possible.

As part of the EMBO publication's Transparent Editorial Process, EMBO reports publishes online a Review Process File to accompany accepted manuscripts. As you are aware, this File will be published in conjunction with your paper and will include the referee reports, your point-by-point response and all pertinent correspondence relating to the manuscript.

If you do NOT want this File to be published, please inform the editorial office within 2 days, if you have not done so already, otherwise the File will be published by default [contact: emboreports@embo.org]. If you do opt out, the Review Process File link will point to the following statement: "No Review Process File is available with this article, as the authors have chosen not to make the review process public in this case." Please note that the author checklist will still be published even if you opt out of the transparent process.

Thank you again for your contribution to EMBO reports and congratulations on a successful publication. Please consider us again in the future for your most exciting work.

Yours sincerely,

Esther Schnapp, PhD
Senior Editor
EMBO reports

****

THINGS TO DO NOW:

Please note that you will be contacted by Wiley Author Services to complete licensing and payment information. The required 'Page Charges Authorization Form' is available here: https://www.embopress.org/pb-assets/embo-site/er_apc.pdf - please download and complete the form and return to embopressproduction@wiley.com

EMBO Press participates in many Publish and Read agreements that allow authors to publish Open Access with reduced/no publication charges. Check your eligibility: https://authorservices.wiley.com/author-resources/Journal-Authors/open-access/affiliation-policies-payments/index.html

You will receive proofs by e-mail approximately 2-3 weeks after all relevant files have been sent to our Production Office; you should return your corrections within 2 days of receiving the proofs.

Please inform us if there is likely to be any difficulty in reaching you at the above address at that time. Failure to meet our deadlines may result in a delay of publication, or publication without your corrections.

All further communications concerning your paper should quote reference number EMBOR-2022-55837V3 and be addressed to emboreports@wiley.com.

Should you be planning a Press Release on your article, please get in contact with emboreports@wiley.com as early as possible, in order to coordinate publication and release dates.

EMBO Press Author Checklist

Corresponding Author Name: Lousie Cheng	
Journal Submitted to: EMBO Reports	
Manuscript Number: EMBOR-2022-55837V2	

USEFUL LINKS FOR COMPLETING THIS FORM
The EMBO Journal - Author Guidelines
EMBO Reports - Author Guidelines
Molecular Systems Biology - Author Guidelines
EMBO Molecular Medicine - Author Guidelines

Reporting Checklist for Life Science Articles (updated January
This checklist is adapted from Materials Design Analysis Reporting (MDAR) Checklist for Authors. MDAR establishes a minimum set of requirements in transparent reporting in the life sciences (see Statement of Task: 10.31222/osf.io/9sm4x). Please follow the journal's guidelines in preparing your
Please note that a copy of this checklist will be published alongside your article.

Abridged guidelines for figures
1. Data
The data shown in figures should satisfy the following conditions:
- the data were obtained and processed according to the field's best practice and are presented to reflect the results of the experiments in an accurate and unbiased manner.
- ideally, figure panels should include only measurements that are directly comparable to each other and obtained with the same assay.
- plots include clearly labeled error bars for independent experiments and sample sizes. Unless justified, error bars should not be shown for technical
- if n<5, the individual data points from each experiment should be plotted. Any statistical test employed should be justified.
- Source Data should be included to report the data underlying figures according to the guidelines set out in the authorship guidelines on Data

2. Captions
Each figure caption should contain the following information, for each panel where they are relevant:
- a specification of the experimental system investigated (eg cell line, species name).
- the assay(s) and method(s) used to carry out the reported observations and measurements.
- an explicit mention of the biological and chemical entity(ies) that are being measured.
- an explicit mention of the biological and chemical entity(ies) that are altered/varied/perturbed in a controlled manner.
- the exact sample size (n) for each experimental group/condition, given as a number, not a range;
- a description of the sample collection allowing the reader to understand whether the samples represent technical or biological replicates (including how many animals, litters, cultures, etc.).
- a statement of how many times the experiment shown was independently replicated in the laboratory.
- definitions of statistical methods and measures:
  - common tests, such as t-test (please specify whether paired vs. unpaired), simple χ2 tests, Wilcoxon and Mann-Whitney tests, can be unambiguously identified by name only, but more complex techniques should be described in the methods section;
  - are tests one-sided or two-sided?
  - are there adjustments for multiple comparisons?
  - exact statistical test results, e.g., P values = x but not P values < x;
  - definition of 'center values' as median or average;
  - definition of error bars as s.d. or s.e.m.

Please complete ALL of the questions below.
Select "Not Applicable" only when the requested information is not relevant for your study.

Materials

Newly Created Materials	Information included in the manuscript?	In which section is the information available? (Reagents and Tools Table, Materials and Methods, Figures, Data Availability Section)
New materials and reagents need to be available; do any restrictions apply?	Yes	Data availability section

Antibodies	Information included in the manuscript?	In which section is the information available? (Reagents and Tools Table, Materials and Methods, Figures, Data Availability Section)
For **antibodies** provide the following information:<br>- Commercial antibodies: RRID (if possible) or supplier name, catalogue number and or/clone number<br>- Non-commercial: RRID or citation	Yes	Methods

DNA and RNA sequences	Information included in the manuscript?	In which section is the information available? (Reagents and Tools Table, Materials and Methods, Figures, Data Availability Section)
**Short novel DNA or RNA including primers, probes**: provide the sequences.	Not Applicable	

Cell materials	Information included in the manuscript?	In which section is the information available? (Reagents and Tools Table, Materials and Methods, Figures, Data Availability Section)
**Cell lines:** Provide species information, strain. Provide accession number in repository **OR** supplier name, catalog number, clone number, and/**OR** RRID.	Not Applicable	
**Primary cultures:** Provide species, strain, sex of origin, genetic modification status.	Not Applicable	
Report if the cell lines were recently **authenticated** (e.g., by STR profiling) and tested for mycoplasma contamination.	Not Applicable	

Experimental animals	Information included in the manuscript?	In which section is the information available? (Reagents and Tools Table, Materials and Methods, Figures, Data Availability Section)
**Laboratory animals or Model organisms:** Provide species, strain, sex, age, genetic modification status. Provide accession number in repository **OR** supplier name, catalog number, clone number, **OR** RRID.	Yes	methods
**Animal observed in or captured from the field:** Provide species, sex, and age where possible.	Not Applicable	
Please detail **housing and husbandry conditions**.	Yes	methods

Plants and microbes	Information included in the manuscript?	In which section is the information available? (Reagents and Tools Table, Materials and Methods, Figures, Data Availability Section)
**Plants:** provide species and strain, ecotype and cultivar where relevant, unique accession number if available, and source (including location for collected wild specimens).	Not Applicable	
**Microbes:** provide species and strain, unique accession number if available, and source.	Not Applicable	

Human research participants	Information included in the manuscript?	In which section is the information available? (Reagents and Tools Table, Materials and Methods, Figures, Data Availability Section)
If collected and within the bounds of privacy constraints report on age, sex and gender or ethnicity for all study participants.	Not Applicable	

Core facilities	Information included in the manuscript?	In which section is the information available? (Reagents and Tools Table, Materials and Methods, Figures, Data Availability Section)
If your work benefited from core facilities, was their service mentioned in the acknowledgments section?	Yes	acknowledgement

Design

Study protocol	Information included in the manuscript?	In which section is the information available? (Reagents and Tools Table, Materials and Methods, Figures, Data Availability Section)

If study protocol has been **pre-registered, provide DOI in the manuscript**. For clinical trials, provide the trial registration number **OR** cite DOI.	Not Applicable	
Report the **clinical trial registration number** (at ClinicalTrials.gov or equivalent), where applicable.	Not Applicable	

Laboratory protocol	Information included in the manuscript?	In which section is the information available? (Reagents and Tools Table, Materials and Methods, Figures, Data Availability Section)
Provide DOI OR other citation details if **external detailed step-by-step protocols** are available.	Not Applicable	

Experimental study design and statistics	Information included in the manuscript?	In which section is the information available? (Reagents and Tools Table, Materials and Methods, Figures, Data Availability Section)
Include a statement about **sample size** estimate even if no statistical methods were used.	Yes	Figure legends
Were any steps taken to minimize the effects of subjective bias when allocating animals/samples to treatment (e.g. **randomization procedure**)? If yes, have they been described?	Not Applicable	
Include a statement about **blinding** even if no blinding was done.	Not Applicable	
Describe **inclusion/exclusion criteria** if samples or animals were excluded from the analysis. Were the criteria pre-established?<br><br>If sample or data points were omitted from analysis, report if this was due to attrition or intentional exclusion and provide justification.	Not Applicable	
For every figure, are **statistical tests** justified as appropriate? Do the data meet the assumptions of the tests (e.g., normal distribution)? Describe any methods used to assess it. Is there an estimate of variation within each group of data? Is the variance similar between the groups that are being statistically compared?	Yes	methods

Sample definition and in-laboratory replication	Information included in the manuscript?	In which section is the information available? (Reagents and Tools Table, Materials and Methods, Figures, Data Availability Section)
In the figure legends: state number of times the experiment was **replicated** in laboratory.	Yes	Figure legend
In the figure legends: define whether data describe **technical or biological replicates.**	Yes	Figure legend, methods

Ethics

Ethics	Information included in the manuscript?	In which section is the information available? (Reagents and Tools Table, Materials and Methods, Figures, Data Availability Section)
Studies involving **human participants**: State details of **authority granting ethics approval** (IRB or equivalent committee(s), provide reference number for approval.	Not Applicable	
Studies involving **human participants**: Include a statement confirming that **informed consent** was obtained from all subjects and that the experiments conformed to the principles set out in the WMA Declaration of Helsinki and the Department of Health and Human Services Belmont Report.	Not Applicable	
Studies involving **human participants:** For publication of **patient photos**, include a statement confirming that consent to publish was obtained.	Not Applicable	
Studies involving experimental **animals**: State details of **authority granting ethics approval** (IRB or equivalent committee(s), provide reference number for approval. Include a statement of compliance with ethical regulations.	Not Applicable	
Studies involving **specimen and field samples:** State if relevant **permits** obtained, provide details of authority approving study; if none were required, explain why.	Not Applicable	

Dual Use Research of Concern (DURC)	Information included in the manuscript?	In which section is the information available? (Reagents and Tools Table, Materials and Methods, Figures, Data Availability Section)
Could your study fall under dual use research restrictions? Please check biosecurity documents and list of **select agents and toxins** (CDC): https://www.selectagents.gov/sat/list.htm	Not Applicable	
If you used a select agent, is the security level of the lab appropriate and reported in the manuscript?	Not Applicable	
If a study is subject to dual use research of concern regulations, is the name of the **authority granting approval and reference number** for the regulatory approval provided in the manuscript?	Not Applicable	

Reporting

The MDAR framework recommends adoption of discipline-specific guidelines, established and endorsed through community initiatives. Journals have their own policy about requiring specific guidelines and recommendations to complement MDAR.

Adherence to community standards	Information included in the manuscript?	In which section is the information available? (Reagents and Tools Table, Materials and Methods, Figures, Data Availability Section)
State if relevant guidelines or checklists (e.g., **ICMJE, MIBBI, ARRIVE, PRISMA**) have been followed or provided.	Not Applicable	
For **tumor marker prognostic studies,** we recommend that you follow the **REMARK** reporting guidelines (see link list at top right). See author guidelines, under 'Reporting Guidelines'. Please confirm you have followed these guidelines.	Not Applicable	
For **phase II and III randomized controlled trials**, please refer to the **CONSORT** flow diagram (see link list at top right) and submit the CONSORT checklist (see link list at top right) with your submission. See author guidelines, under 'Reporting Guidelines'. Please confirm you have submitted this list.	Not Applicable	

Data Availability

Data availability	Information included in the manuscript?	In which section is the information available? (Reagents and Tools Table, Materials and Methods, Figures, Data Availability Section)
Have **primary datasets** been deposited according to the journal's guidelines (see 'Data Deposition' section) and the respective accession numbers provided in the Data Availability Section?	Yes	Data availability section. accession number not yet available
Were **human clinical and genomic datasets** deposited in a public access-controlled repository in accordance to ethical obligations to the patients and to the applicable consent agreement?	Not Applicable	
Are **computational models** that are central and integral to a study available without restrictions in a machine-readable form? Were the relevant accession numbers or links provided?	Not Applicable	
If publicly available data were reused, provide the respective **data citations in the reference list**.	Not Applicable	
